# Hagfish from the Cretaceous Tethys Sea and a reconciliation of the morphological–molecular conflict in early vertebrate phylogeny

**DOI:** 10.1073/pnas.1814794116

**Published:** 2019-01-22

**Authors:** Tetsuto Miyashita, Michael I. Coates, Robert Farrar, Peter Larson, Phillip L. Manning, Roy A. Wogelius, Nicholas P. Edwards, Jennifer Anné, Uwe Bergmann, A. Richard Palmer, Philip J. Currie

**Affiliations:** ^a^Department of Organismal Biology and Anatomy, University of Chicago, Chicago, IL 60637;; ^b^Department of Biological Sciences, University of Alberta, Edmonton T6G 2E9, Canada;; ^c^Black Hills Institute of Geological Research, Hill City, SD 57745;; ^d^School of Earth and Environmental Sciences, University of Manchester, M13 9PL Manchester, United Kingdom;; ^e^Stanford Synchrotron Radiation Lightsource, SLAC National Accelerator Laboratory, Menlo Park, CA 94025;; ^f^The Children’s Museum of Indianapolis, Indianapolis, IN 46208

**Keywords:** Myxinoidea, cyclostome, monophyly, synchrotron, soft tissue

## Abstract

Hagfish depart so much from other fishes anatomically that they were sometimes considered not fully vertebrate. They may represent: (*i*) an anatomically primitive outgroup of vertebrates (the morphology-based craniate hypothesis); or (*ii*) an anatomically degenerate vertebrate lineage sister to lampreys (the molecular-based cyclostome hypothesis). This systematic conundrum has become a prominent case of conflict between morphology- and molecular-based phylogenies. To date, the fossil record has offered few insights to this long-branch problem or the evolutionary history of hagfish in general, because unequivocal fossil members of the group are unknown. Here, we report an unequivocal fossil hagfish from the early Late Cretaceous of Lebanon. The soft tissue anatomy includes key attributes of living hagfish: cartilages of barbels, postcranial position of branchial apparatus, and chemical traces of slime glands. This indicates that the suite of characters unique to living hagfish appeared well before Cretaceous times. This new hagfish prompted a reevaluation of morphological characters for interrelationships among jawless vertebrates. By addressing nonindependence of characters, our phylogenetic analyses recovered hagfish and lampreys in a clade of cyclostomes (congruent with the cyclostome hypothesis) using only morphological data. This new phylogeny places the fossil taxon within the hagfish crown group, and resolved other putative fossil cyclostomes to the stem of either hagfish or lamprey crown groups. These results potentially resolve the morphological–molecular conflict at the base of the Vertebrata. Thus, assessment of character nonindependence may help reconcile morphological and molecular inferences for other major discords in animal phylogeny.

## Significance

Jawless, boneless, and virtually without fossil record, hagfish have long escaped systematists’ grip on their place among other fish. Yet their systematic resolution is critical to define vertebrates as a clade. Here we report an unequivocal fossil hagfish from the Cretaceous Mediterranean. Using this fossil to calibrate the evolutionary history of the group, our analysis supports hagfish and lampreys as sister groups, which likely diverged from one another in early Paleozoic times. As a result, vertebrates have a deep dichotomy, where some fossil jawless vertebrates sit closer to hagfish and lampreys than to jawed vertebrates. We showed that morphology-based analysis converged onto molecular inferences when characters are coded nonindependently, providing a case study for morphological–molecular conflicts in animal phylogeny.

Hagfish and lampreys (cyclostomes) represent the only living lineages of jawless vertebrates (or agnathans) (1). Cyclostomes are generally considered primitive morphologically with respect to jawed vertebrates (gnathostomes) (1, 2). However, consensus is still lacking about cyclostome relationships. None of the traits proposed to unite hagfish and lampreys sets them apart clearly from extinct lineages of jawless vertebrates (3, 4). Furthermore, characters distribute asymmetrically. Hagfish have fewer traits than lampreys in common with gnathostomes, and are characterized by rudimentary state or absence of otherwise pan-vertebrate traits such as electroreceptors or taste buds (5, 6). Thus, resolving the cyclostome relationships holds direct implications for defining the vertebrate crown group, and for describing early character evolution in that clade. Motivated by this prospect, a number of analyses have set out to clarify positions of hagfish and lampreys with respect to gnathostomes.

**Fig. 1.**
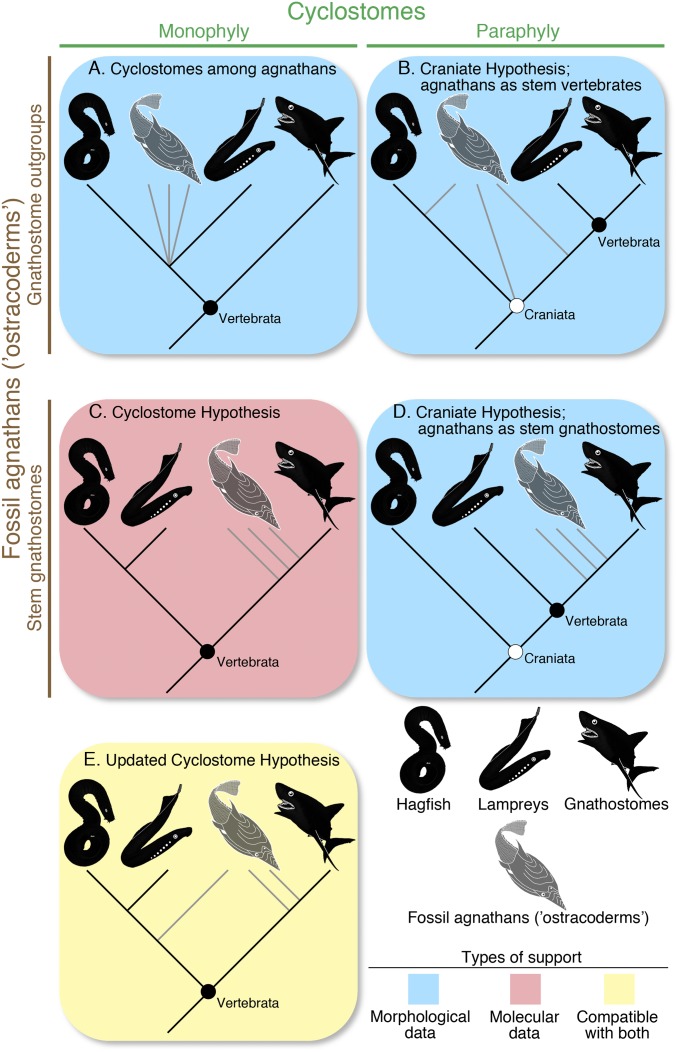
A summary of hypotheses for cyclostome relationships. The hypotheses differ in cyclostome monophyly or paraphyly (columns), and about relationships of fossil “agnathans” with respect to the cyclostome and gnathostome crowns (rows). Blue box = support from morphological data; red box = support from molecular data. Yellow box = compatible with both data types. (*A*) Noncladistic classification schemes based on morphological data with cyclostomes nested among agnathans. (*B*) Craniate hypothesis in early cladistic analyses based on morphological data, where hagfish and fossil agnathans become nested outside the crown vertebrate node. (*C*) Cyclostome hypothesis as supported by molecular data. Fossil agnathans are assumed on the gnathostome stem. (*D*) Craniate hypothesis in recent analyses of morphological data, where hagfish remain nested outside the crown vertebrate node. Fossil agnathans are placed on the gnathostome stem. (*E*) Updated cyclostome hypothesis supported by the analyses of morphological data presented in this paper. Hagfish and lampreys form a clade of cyclostomes as supported by the molecular inferences. However, some fossil agnathans are closer to the cyclostome crown than to the gnathostome crown.

The endeavor has generated variants of four main topological schemes (Fig. 1 *A–D*). Early classifications split vertebrates between agnathans and gnathostomes, with cyclostomes nested within the former (Fig. 1*A*) (7). With the advent of cladistics, this dichotomy was challenged: hagfish fell outside the vertebrate node, rendering cyclostomes paraphyletic (craniate hypothesis; Fig. 1*B*) (8). More recent analyses of morphological data continue to support the paraphyly, but differ from the earlier analyses in placing fossil agnathans on the gnathostome stem (Fig. 1*D*) (9–16). Meanwhile, cyclostome paraphyly has been contested by molecular phylogenetics, which nearly unanimously favor cyclostomes as a clade (cyclostome hypothesis; Fig. 1*C*) (17, 18). Thus, the controversy has been locked in a stalemate between the molecular inferences for cyclostome monophyly (Fig. 1*C*) and the morphologically supported cyclostome paraphyly (Fig. 1*D*).

Despite the ever more complete molecular datasets, consensus has not been achieved. This is because (*i*) monophyly is an anticipated outcome of long-branch attraction in molecular data; and (*ii*) fossil taxa that potentially break up the long branches are only accessible via morphological data. Interpretations of character polarities are radically different between the two topologies. The morphology-supported paraphyly implies rudimentary state or absence of traits in hagfish as primitive (8). For the monophyly, molecular trees are agnostic about character polarities, but the rudiments and absence in hagfish tend to be interpreted as secondary (2, 3). An equally parsimonious alternative— hagfish as primitive and lampreys and gnathostomes as convergent—is rarely discussed (2). As such, cyclostome relationships provide a classic example of long-branch problems hindering morphological–molecular congruence.

Although fossils are critical in reducing branch lengths, the patchy fossil record of cyclostomes remains a formidable challenge. For hagfish (myxinoids), three soft-bodied Carboniferous forms (*Gilpichthys*, *Myxineidus*, and *Myxinikela*) have each been posited as a stem myxinoid (19–21), but myxinoid affinities for the former two have been questioned (9, 22, 23). *Myxinikela* is the sole putative myxinoid with any degree of cladistic support (9), but its similarities to living hagfish may partly be artifacts of preservation (23). As these Carboniferous forms are only preserved with a handful of morphological structures, the inferences have been based on a few myxinoid- or cyclostome-diagnostic characters (potential keratinous teeth in *Gilpichthys*; a longitudinally elongate nasohypophyseal system in *Myxinikela*) and the general absence of lamprey-like traits (e.g., oral funnel, discrete dorsal fins) (19–21). Consequently, living hagfishes imply long ghost lineages, making it difficult to interpret the peculiar morphology of the group. Here, we report an unequivocal hagfish fossil from the Late Cretaceous of Lebanon—which reduces this gap in the fossil record by 100 My—and present an updated morphology-based phylogeny of cyclostomes. We also describe the exquisitely preserved soft-tissue anatomy of this new hagfish using trace elements mapped by synchrotron rapid-scanning X-ray fluorescence (SRS-XRF; described in *Methods* and *SI Appendix*, Part B).

## Systematic Paleontology

Vertebrata Linnaeus, 1758; Cyclostomi Duméril, 1806; Myxinoidea Müller, 1834; *Tethymyxine tapirostrum* gen. et sp. nov.

### Holotype.

**Fig. 2.**
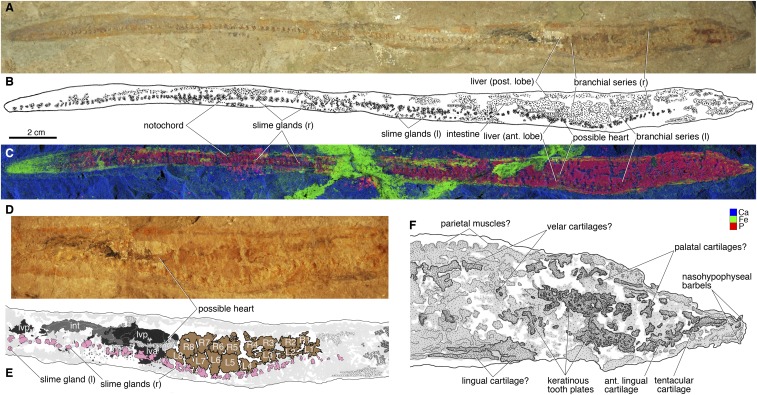
*Tethymyxine tapirostrum* gen. et sp. nov, a fossil hagfish from the Cenomanian of Lebanon. Holotype (BHI 6445) in right lateral view: (*A*) photograph; (*B*) interpretive drawing; (*C*) false-color composite of distributions of three selected chemical elements (blue = Ca; green = Fe; red = P; Ca and Fe in HZ setup and P in LZ setup, 99.9% threshold) from SRS-XRF. The visceral anatomy of BHI 6445 in composite photograph (*D*) and interpretive drawing (*E*) in the following color codes: black = liver lobes; brown = branchial pouches; dark gray = intestine; light gray = preserved amorphous tissues; pink = slime glands; stippled gray = other soft tissues that are preserved with distinct outlines. The cranial anatomy of BHI 6445 in interpretive drawing (*F*) in which preserved structures are indicated in gray shades and stipples. Abbreviations: ant, anterior; int, intestine; L, left branchial pouch; l, left side; lva, liver, anterior lobe; lvp, liver, posterior lobe; post, posterior; R, right branchial pouch; r, right side.

Black Hills Institute of Geological Research (BHI) 6445. A complete body fossil, exposed in lateral aspect (Fig. 2 and *SI Appendix*, Figs. S1–S4).

### Etymology.

The generic name is derived from “Tethys” after the Tethys Sea and “myxinos” (Latinized Greek for “slimy fish”). The specific name is derived from “tapirus” (Latinized Old Tupi for tapir) and “rostrum” (a Latin root for snout) for the elongated tapering snout.

### Horizon and Locality.

The type specimen was collected from sublithographic limestones of Hâdjula Lagerstätte (Cenomanian, Upper Cretaceous), 10 km east of Byblos, Lebanon. The locality is interpreted as an interreef basin on a carbonate platform (24). See *SI Appendix*, Part A for detailed information on provenance and geological settings.

### Diagnosis.

*Tethymyxine tapirostrum* is a myxinoid exhibiting the following unique combination of characters: tapering nasohypophyseal profile; tentacular cartilage not extending beyond nasohypophyseal aperture; prebranchial length nearly equal to branchial length; 8 pairs of branchial pouches; 133 slime glands on one side; and caudal fin not expanded into a round lobe.

### Description and Comparisons.

In visible light, BHI 6445 (Fig. 2) is preserved in full-body outline with soft tissues (body length = 313 mm). The trunk lies on its left side, whereas the head was twisted counterclockwise to expose the ventral side. The preserved tissues are marked by greater abundance of P, Mn, Fe, Cu, and Zn relative to the surrounding matrix (Fig. 2*C* and *SI Appendix*, Fig. S3). These tissues are inferred to be (*i*) composed of collagenous extracellular matrix (e.g., chondrocranium); (*ii*) enclosed by a highly vascularized epithelial sheet (e.g., branchial pouches, livers); or (*iii*) secreted keratin (e.g., infillings of slime glands, tooth-like apparatus). The original outline of the body is clearly demarcated from the matrix in most elements mapped by SRS-XRF (Fig. 2*C* and *SI Appendix*, Fig. S3). Al, P, S, Mn, Fe, Cu, Zn, and As are markedly more abundant in the preserved tissues than in the matrix, whereas the reverse is true for Si, Ca, and Hg. The preservation of structures and imprints is partly obscured by glue and paint, but the fossilized tissues can be distinguished from artifacts. Optically, the structural preservation is distinguished by surface topography under the artifacts. In SRS-XRF, the artifacts are clearly differentiated from the fossil and matrix based on distributions of S, Ti, V, and Fe. In particular, X-ray absorption spectroscopy reveals differential distributions of sulfur species in this specimen (*SI Appendix*, Fig. S4). Sulfate is the dominant species in all residues; organic sulfur is present in both the fossil and matrix, but virtually absent in the glue (*SI Appendix*, Part B). This result highlights the utility of SRS-XRF in identifying curatorial artifacts in fossil specimens.

The preservation of decay-prone soft tissues such as branchial pouches and slime (Fig. 2) suggests that BHI 6445 was rapidly buried (25). Although the mode of preservation is similar to that of the Early Cretaceous freshwater lamprey *Mesomyzon* from the Jehol Group of China, internal structures of BHI 6445 are not obscured by the preserved integument and muscles as in specimens of *Mesomyzon* (26) (for detailed discussion, see *SI Appendix*, Part A).

The overall characteristics clearly reject nonmyxinoid hypotheses for the identity of BHI 6445. The absence of a mineralized skeleton, paired fins, and jaws rules out anguilliform gnathostomes like the coeval eel *Luenchelys* (27). BHI 6445 is not a lamprey either, because it does not have an oral sucker, tectal cartilages, branchial basket, dorsal fins, and other skeletal and proportional features of lampreys that would have been present given the state of preservation (25). Instead, BHI 6445 has characters diagnostic of myxinoids, including an elongate nasohypophyseal portion of the chondrocranium, posteriorly placed branchial pouches, and slime glands.

**Fig. 3.**
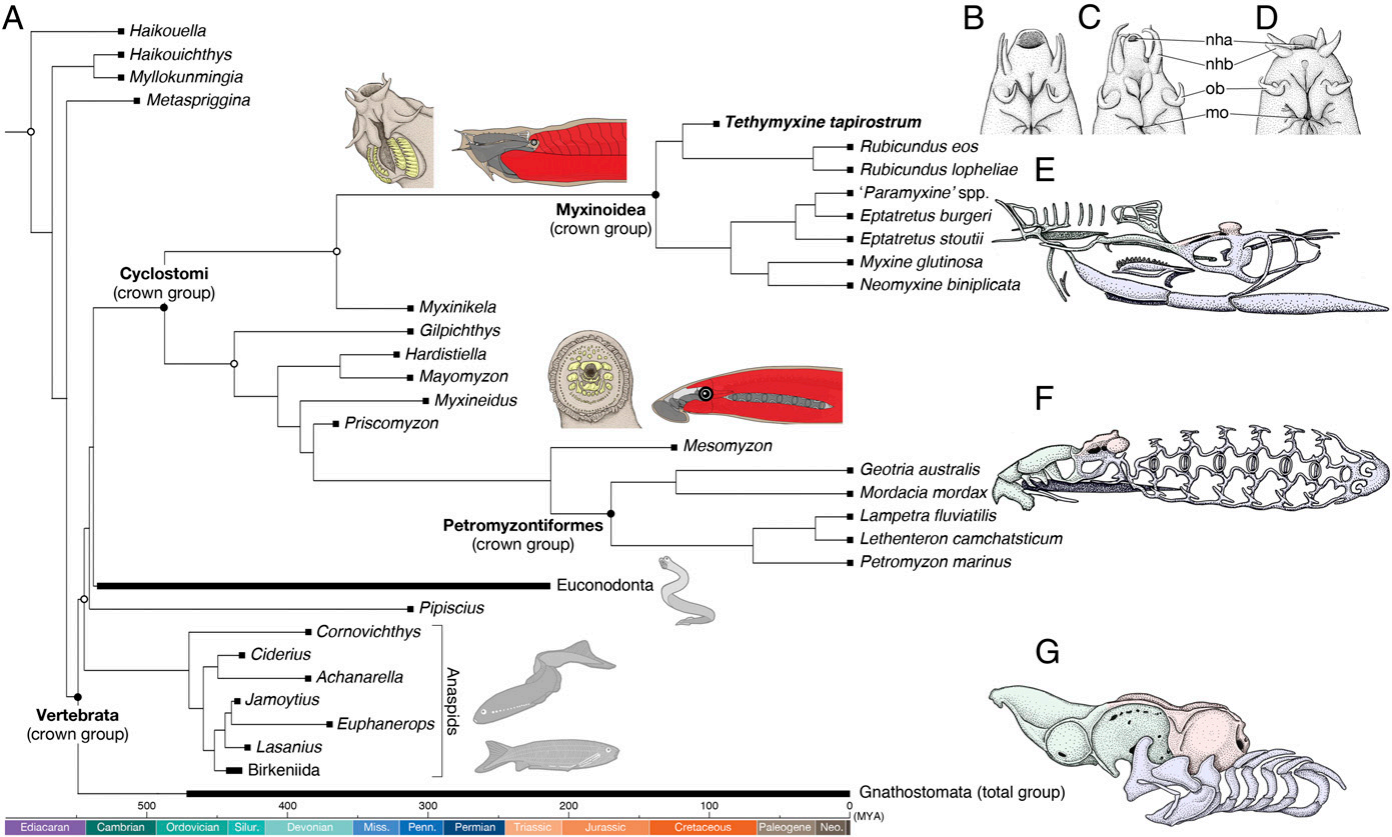
A time-scaled phylogenetic tree of cyclostomes. (*A*) Summary tree showing cyclostome relationships. Maximum parsimony and Bayesian inferences converged onto each other in placing *Tethymyxine* within the hagfish crown group and supporting cyclostome monophyly. The precise topology is from the maximum parsimony analysis (*SI Appendix*, Fig. S6). Node ages represent median of 95% HPD distribution in a Bayesian molecular clock analysis of mitogenomic sequences (16S and COI) under fossilized birth–death model (see *SI Appendix*, Fig. S9 for a fully calibrated maximum clade credibility tree with node intervals). The crown group of cyclostomes is united by at least two morphological characters (shown on each stem): keratinous tooth plates (yellow) and periocular position of trunk muscles (red). At *Top Right*, the nasohypophyseal profiles are compared in ventral view among three selected crown-group hagfishes (*B*: *Tethymyxine tapirostrum*; *C*, *Rubicundus eos*; *D*, *Eptatretus stoutii*; the latter two based on ref. 29). To show morphological divergence among the three major crown groups of living vertebrates (*E*, myxinoids; *F*, petromyzontiforms; *G*, gnathostomes), each is accompanied by a chondrocranium in left lateral view (green: neural crest-derived nasohypophyseal skeleton; red: mesodermally derived neurocranium; blue: neural crest-derived pharyngeal skeleton). Filled squares represent occurrences of the terminal taxa. Crown nodes are each indicated by a filled circle, and total nodes by an empty circle. Abbreviations: nha, nasohypophyseal aperture; nhb, nasohypophyseal barbels; mo, mouth; ob, oral barbels.

*Tethymyxine* has a small head relative to its body size. The prebranchial length of BHI 6445 is shorter than that in living hagfishes, relative to the total body length (13% versus 20–30%) (28). As a consequence, the first branchial pouch sits more anteriorly than in living hagfish species, leaving a relatively short space for the lingual apparatus to occupy. The snout tapers toward the nasohypophyseal aperture (nostril). The nasohypophyseal barbels are preserved with the sigmoidal tentacular cartilage on the left side of BHI 6445. The barbels sit behind the aperture as in living *Rubicundus* (29) (Fig. 3 *B* and *C*), but the specimen differs from it in lacking a protruded nasohypophyseal tube. The tentacular cartilage is fused posteriorly with the lateral element of the anterior lingual cartilages. Both the left and right parts of the keratinous tooth plates are preserved in association with the anterior lingual cartilages. Although several cusps can be identified by the distributions of Ca, Mn, Fe, Ni, and Hg (*SI Appendix*, Fig. S3), precise cusp number and shape cannot be determined. Unlike the preservation of comb-like tooth plates in *Gilpichthys* from Mazon Creek (20), the plates may have been pyrolysated in BHI 6445 as seen in the scales of actinopterygians from the same locality (27). Presumably the missing counterpart of BHI 6445 has the rest of preserved chondrocranial elements.

*Tethymyxine* has eight pairs of branchial pouches. This number is greater than in *Rubicundus* spp. (*n* = 5) and most other living hagfishes (*n* = 4∼7), equal to some species of *Eptatretus* and *Nemamyxyne*, and smaller than in some species of *Eptatretus* (*n* = 14 in *E. polytrema*) (28). A structure immediately behind the branchial series is topographically and morphologically consistent with a heart, which has Fe in higher abundance than in the surrounding tissues (Fig. 2 *D* and *E*). The intestine extends between the anterior and posterior liver lobes. These visceral tissues have different textures and colors that allow clear delineation under natural light (*SI Appendix*, Fig. S2). However, the element mapping by SRS-XRF shows similar compositions among these tissues. P, S, and Cl appear to have greater abundances in the branchial pouches than in the intestine, and Ca seems to be the reverse (*SI Appendix*, Fig. S3).

A characteristic unique to hagfish, slime glands are preserved in an uninterrupted series of infillings that extends nearly the entire length of the body on the ventral side. The infillings are distinguished from other tissues by surface profile, high Ca, P, and S abundances, and higher levels of Cl, Ti, Mn, Fe, Cu, Ni, Zn, and Hg relative to the matrix (Fig. 2 and *SI Appendix*, Figs. S2 and S3 and Table S1). The chemical mapping is consistent with the fact that a major component of hagfish slime is tightly coiled, mucin-coated α-keratin threads (30)—pyrolysates of which are characterized by calcium phosphatic content (31). The number of the slime glands (*n* = 133 on the right) is greater than in most living hagfishes (*n* = 70∼110), but close to that of *R. eos* (*n* = 128∼130) (28). The end of the tail is obscured by paint, but it is possible to outline the body by relatively high concentrations of Cu, P, S, and Fe (contained in paint, but higher in preserved tissues) and relatively low concentrations of Ca (Fig. 2*C* and *SI Appendix*, Figs. S3 and S4 and Table S1). The notochord is difficult to identify optically (as it is generally in fossils of soft-bodied vertebrates), but can be delineated with the high P and S concentrations along the dorsal midline (*SI Appendix*, Figs. S3 and S4 *B* and *C*). The tail tapers rather than expanding into a round lobe.

### Phylogenetics.

We generated a morphological dataset to test the relationships of *Tethymyxine* and cyclostomes. *Tethymyxine* has myxinoid apomorphies not identified in other fossil taxa. These traits include the posteriorly placed branchial series and slime glands, which are absent, missing, or ambiguous in *Myxinikela* and other putative fossil cyclostomes (9). To code these unequivocal and potential myxinoids in a phylogenetic dataset, we reevaluated morphological characters that are nonindependent, and enhanced contingency coding to reduce implicit weighting (32). For example, attributes of a mineralized skeleton such as bones and scales are not simply absent in the soft-bodied myxinoids. Those characters are inapplicable in this lineage. Applying no character values to attributes of biomineralization in myxinoids removes additive weight of lacking mineralization in every coded skeletal element. This contingency coding eliminated, on average, 70% of character information from nonvertebrate outgroups and 32% from cyclostomes, which would otherwise be available to coding (*SI Appendix*, Parts B–D and Fig. S5 for comparative analysis).

Searches of this dataset, using both maximum parsimony and Bayesian methods, resolved *Tethymyxine* within the myxinoid crown group, sister to species of the living genus *Rubicundus* (Fig. 3*A* and *SI Appendix*, Figs. S6–S8). These two genera with an elongate snout, indented barbels, and a large number of slime glands (>100) (Fig. 3 *B* and *C*) were nested outside the clade of more conventional living myxinoids (*Eptatretus*, *Myxine*, and *Neomyxine*) (Fig. 3*D*). *Myxinikela* was resolved as a stem myxinoid, whereas other Carboniferous forms (*Gilpichthys*, *Hardistiella*, *Mayomyzon*, and *Myxineidus*) and *Priscomyzon* were found along the stem of lampreys. Under maximum parsimony, the crown group of cyclostomes is united by two unambiguous character changes: keratinous tooth plates and migration of postotic myomeres to position of eyes (Fig. 3*A* and *SI Appendix*, Table S3). Euconodonts, *Pipiscius*, and anaspids fell into the cyclostome stem. Together, these lineages comprised the total group of cyclostomes. Controversial Cambrian forms (*Haikouella*, *Haikouichthys*, *Metaspriggina*, and *Myllokunmingia*) were resolved in the vertebrate stem.

Given the topology obtained in the maximum parsimony analysis, and using the fossil-calibrated dataset of mitogenomic sequences, tip-dated Bayesian estimates of divergence placed the crown cyclostome node from the earliest Cambrian (Terreneuvian) to Early Silurian (Wenlockian) times [95% highest posterior density interval (HPD): 536.31–428.44 Mya] with median near the Cambrian–Ordovician boundary (485.57 Mya) (*SI Appendix*, Fig. S9). Both the myxinoid and petromyzontiform crowns were estimated to have their origins in Mesozoic times, with the myxinoid estimates straddling the Jurassic–Cretaceous boundary (95% HPD: 183.95–103.30 Mya; median: 138.59 Mya) and with the petromyzontiform estimates within the Jurassic times (95% HPD: 194.84–163.14 Mya; median: 169.82 Mya).

## Discussion

*Tethymyxine* is significant as being the sole fossil of the crown group Myxinoidea. It therefore provides a crucial calibration point for the evolution of the myxinoid clade as well as the distinctive morphology of the group. In our dataset, the myxinoid topologies were robust to altered character coding schemes (assuming taphonomic artifacts) or different taxonomic combinations (*SI Appendix*, *Part C* and Fig. S8). Multiple traits of the myxinoid crown in *Tethymyxine* are lacking in the Carboniferous stem myxinoid *Myxinikela*. In *Myxinikela*, the branchial apparatus sits well within the cranial domain, and the liver appears to be a single lobe. Given its state of preservation, α-keratin in the slime glands would have been preserved if present in *Myxinikela*. Thus, these crown myxinoid traits were likely acquired along the remaining 200–250 My ghost lineage (Fig. 3*A*).

Our analyses reveal cyclostomes as a clade using morphological data. Both maximum parsimony and Bayesian analyses converged on this result with the same clade membership (*SI Appendix*, Figs. S6 and S7). The dataset performed consistently with theoretical predictions (33). Maximum parsimony and Bayesian inferences generated trees that are largely congruent with each other. The maximum parsimony trees are better resolved (more precise) than the Bayesian trees. Importantly, cyclostome monophyly based on morphology corroborates the topology consistently supported by molecular data (17, 18). The fossil-calibrated mitogenomic molecular clock (*SI Appendix*, Fig. S9) also produced divergence estimates for cyclostome crown clades that are largely congruent with the previous molecular clock analysis of nuclear genes (34). For the cyclostome crown, our divergence estimates (536.31–428.44 Mya) agree well with the previous estimates (480–430 Mya). Calibrated with *Tethymyxine* (100.5–93.9 Mya), the myxinoid crown is now pushed to an older interval (183.95–103.30 Mya) than in the previous analysis (90–40 Mya). The petromyzontiform crown is estimated shallower (194.84–163.14 Mya) than in the analysis of nuclear genes (280–220 Mya), but the split between the southern and northern hemispheric clades is constrained to before the separation and breakup of Gondwana in both analyses (Fig. 3*A* and *SI Appendix*, Fig. S9) (34).

This morphological–molecular congruence is a significant update of the cyclostome hypothesis (Fig. 1 *C* and *E*). One principal advance is phylogenetic resolution of fossil jawless vertebrates under cyclostome monophyly. These fossil forms include (*i*) putative stem taxa of cyclostome lineages (e.g., *Myxinikela*), and (*ii*) lineages that have been placed on the gnathostome stem under cyclostome paraphyly (e.g., anaspids and euconodonts). Molecular datasets contain no information from the fossils, but previous schemes of the cyclostome hypothesis assumed the taxa within cyclostomes and the taxa on the gnathostome stem without including them all in a single cladistic test (18). The stem cyclostome status of anaspids and euconodonts has never been supported quantitatively in previous analyses (Fig. 3*A* and *SI Appendix*, Figs. S6 and S8). Furthermore, our Bayesian analysis (*i*) did not resolve the polytomy of several other lineages with cyclostome and gnathostome total clades; and (*ii*) supported euconodonts as stem myxinoids (*SI Appendix*, Fig. S7). These results are reminiscent of early classification schemes of cyclostomes (Fig. 1 *A* and *B*) and precladistic attempts to classify conodonts, but have the basis in different characters.

Our morphological dataset has a long list of characters that support cyclostome monophyly and stem cyclostome status of anaspids and euconodonts (*SI Appendix*, Table S3). The crown has two unambiguous synapomorphies in keratinous tooth plates and periocular position of trunk myomeres (Fig. 3*A*). The characters posited previously as cyclostome synapomorphies (e.g., pulley-like feeding apparatus) (4) are either not preserved in fossils [thus optimized to the crown cyclostome node only under delayed transformation (DELTRAN)] or symplesiomorphic to cyclostomes. In comparison, keratin is decay-resistant relative to other soft tissues (25), and myomeric positions can be determined using a number of osteological correlates, such as postbranchial wall in osteostracans (10). As for euconodonts, their position in the cyclostome stem is supported by the lack of anterior extension of the midline fin and the presence of denticulate elements within the buccal cavity (*SI Appendix*, Table S3). Recent attempts to associate conodonts with cyclostomes (or myxinoids) were based on the inferred presence of a pulley-like feeding apparatus (35) or the alleged homology of conodont elements with the keratinous tooth plates in living cyclostomes (36, 37). Both of these characters were coded as ambiguous in euconodonts in our dataset (*SI Appendix*, Fig. S7). Nevertheless, the Bayesian inferences supported the stem-myxinoid position. For parsimony, the cyclostome crown collapsed after coding these ambiguous characters for conodonts as seen in living cyclostomes (*SI Appendix*, Fig. S8*C*).

The morphological support for cyclostome monophyly emerged after enhancement of contingency coding method. Nearly all cyclostome synapomorphies in our dataset were included in previous morphological datasets in various definitions and coding schemes, and would have been optimized as cyclostome synapomorphies if the topology were constrained (*SI Appendix*, Table S4). These datasets suggest a number of other characters as potentially synapomorphic to cyclostomes under the same topological constraint. In our dataset, most of those additional characters are symplesiomorphic to cyclostomes (denoted with “§,” *SI Appendix*, Table S4) optimized in opposite character polarity (**), or parsimony-uninformative and not included (‡)—all consequences of assigning nonapplicable scores within those characters (*SI Appendix*, Parts D and E). Recoding nonapplicable scores to discrete character states resurrected paraphyletic cyclostomes (*SI Appendix*, Fig. S8*A*).

Our phylogeny implies hagfish morphology as specialized, not primitive. In myxinoids, trait absence or underdeveloped condition has been difficult to interpret—whether the absence (e.g., electroreceptors or extrinsic eye musculature) represents an ancestral or secondary condition, or whether the underdeveloped traits (e.g., vertebrae) are rudimentary or vestigial. Hagfish nested within cyclostomes suggest that these are derived states. Indeed, a large number of reversal events are optimized between the total and crown myxinoid nodes under both accelerated transformation (ACCTRAN) and DELTRAN (*SI Appendix*, Table S3). Regarding those listed traits, hagfish appear to have lost them secondarily, in line with the narrative of the cyclostome hypothesis (5, 38). These character changes are parallel with acquisitions of the traits unique to hagfish, such as slime glands (*SI Appendix*, Table S3). Even cyclostomes on the whole appear to be rather specialized than primitive. The last common ancestor of all living vertebrates has often been posited as a soft-bodied hagfish/lamprey-like form (1). However, the stem cyclostome status of anaspids and euconodonts in the new phylogeny implies that the potential to mineralize skeletal matrix predated the cyclostome crown. This scenario is consistent with observations that modern hagfish and lampreys deposit calcium phosphates to line their inner ears (39), or that lamprey cartilages spontaneously calcify in vitro (40).

## Conclusions

An assessment of nonindependence among morphological characters may lead to unexpected congruence when a phylogenetic dispute is locked in a stalemate between internally consistent morphological and molecular inferences. Our phylogenetic analysis of cyclostomes provides one such case study. In this phylogeny of cyclosotmes, the new fossil hagfish *Tethymyxine* represents an important calibration point not just for the divergence of the myxinoid crown, but also for many morphological traits (e.g., slime glands) that set hagfish apart from all other vertebrates. The morphologically supported Cyclostomi implies some of the characters conventionally considered primitive in hagfish (e.g., the absence of extraocular muscles, electroreceptors, or taste buds) as specialized. This view is consistent with the developmental features of hagfish interpreted as vestigial and derived (e.g., axial skeleton) (38, 41). Between two living cyclostome lineages, lampreys may serve as a better outgroup to gnathostomes, but a full extent of their utility as an outgroup remains unknown until evolutionary origins of the peculiar life history (distinct larval phase with dramatic metamorphosis) are resolved (42, 43).

## Methods

SRS-XRF was performed at beam line 6–2 at the Stanford Synchrotron Radiation Lightsource (SSRL), SLAC National Accelerator Laboratory. SRS-XRF is a noninvasive technique that can spatially resolve chemical element composition in large samples in short time frames (44). For the new phylogeny, three lines of analyses were performed: (*i*) maximum parsimony analyses; (*ii*) nonclock Bayesian analyses; and (*iii*) clock analyses. The morphological dataset was the same for all of the analyses and included 52 taxa (*SI Appendix*, Table S2) and 168 characters (*SI Appendix*, Part E). For the parsimony analyses, heuristic search was used with no topological constraint. For the non-clock-based Bayesian analyses, each analysis was run for 5 million generations with 2 runs of 4 chains, without topological constraint, and sampling every 5,000 generations. Finally, we generated a dataset with 16S and COI sequences for extant taxa and with fossil calibrations. This dataset was subjected to a Bayesian analysis under the fossilized birth–death model, given topology obtained from the parsimony analysis. This analysis provided divergence estimates shown in *SI Appendix*, Fig. S9. For full description, see *SI Appendix*, Parts B–E. Original data for SRS-XRF and phylogenetic analyses are placed in a permanent depository (45).

## Supplementary Material

Supplementary File

Supplementary File

Supplementary File

Supplementary File

Supplementary File
