# Supplementary material for: Hagfish from the Cretaceous Tethys Sea and a reconciliation of the morphological–molecular conflict in early vertebrate phylogeny
Source: Proc Natl Acad Sci U S A. 2019 Jan 22;116(6):2146–51. doi: 10.1073/pnas.1814794116 (PMC6369785; doi:10.1073/pnas.1814794116)
Supplement: Supplementary File [file pnas.1814794116.sapp.pdf]

Supplementary Appendix:

Hagfish from the Cretaceous Tethys Sea and a Reconciliation of the Morphological-Molecular Conflict in Early Vertebrate Phylogeny

Tetsuto Miyashita[1, 2], Michael I. Coates[1], Robert Farrar[3], Peter Larson[3], Phillip L. Manning[4], Roy A. Wogelius[4], Nicholas P. Edwards[4,5], Jennifer Anné[4,6], Uwe Bergmann[5], A. Richard Palmer[2], Philip J. Currie[2]

[1]Department of Organismal Biology and Anatomy, University of Chicago. Chicago, Illinois, USA 60637-1508

[2]Department of Biological Sciences, University of Alberta. Edmonton, Canada T6G 2E9

[3]Black Hills Institute of Geological Research. Hill City, South Dakota, USA 57745

[4]School of Earth and Environmental Sciences, University of Manchester. Manchester, UK M13 9PL

[5]Stanford Synchrotron Radiation Lightsource, SLAC National Accelerator Laboratory, 2575 Sand Hill Road, Menlo Park, California, USA 94025

[6]The Children's Museum of Indianapolis, Indianapolis, Indiana, USA 46208

To whom correspondence should be addressed: Tetsuto Miyashita, email: tetsuto@uchicago.edu

Cladistic datasets and additional data files available at DOI: 10.6084/m9.figshare.7545002

Digital data from SRS-XRF available at DOI: 10.6084/m9.figshare.7545002

Table of Contents

Supplementary Text

Part A. Additional Information on Holotype………………………………………………… 4

A1. Taphonomy and Paleoecological Implications……………………………………… 4

A2. Provenance……………………………………………………………………….. 5

Part B. Methods…………………………………………………………………………… 6

B1. SRS-XRF………………………………………………………………………. 6

B2. Phylogenetic Analyses………………………………………………………… 8

B2a. Overview……………………………………………………………… 8

B2b. Maximum Parsimony Analyses……………………………………… 10

B2c. Bayesian Analyses…………………………………………………… 10

Part C. Results of Phylogenetic Analyses………………………………………………... 12

C1. Maximum Parsimony Analyses……………………………………………….. 12

C2. Bayesian Inferences…………………………………………………………… 14

C3. Morphological Support for Cyclostome Monophyly……………………………….. 15

C4. Additional Analyses Using Different Combinations of Taxa and Characters………. 16

C4a. Analysis of Characters for Crown-group Cyclostomes……………………. 16

C4b. Analysis of Characters for Conodonts……………………………………... 18

C4c. Analysis of Characters for *Pipiscius*…………………………………………….. 19

C4d. Analysis of Characters for *Gilpichthys*……………………………………….. 20

C4e. Analysis of Characters for Euphaneropids………………………………… 21

C4f. Analysis of Characters for Cambrian Taxa…………………………………. 22

C4g. Questionable Myxinoid Affinity of *Palaeospondylus*……………………... 23

C4h. Exclusion of *Tullimonstrum*………………………………………………….. 24

C4i. Concluding Statements for Secondary Analyses…………………………… 25

Part D. Character and Taxon Sampling………………………………………………… 26

D1. Sampling Strategies, Overview……………………………………………….... 26

D2. Excluded Characters…………………………………………………………… 28

D3. Included Characters……………………………………………………………. 33

Part E. List of Characters………………………………………………………………… 34

E1. Brain, Sensory, and Nervous System……………………………………………... 34

E2. Mouth and Branchial System…………………………………………………... 46

E3. Circulatory System…………………………………………………………….. 56
E4. Fins and Fin-folds…………………………………………………………… 58
E5. Skeletal System……………………………………………………………… 62
E6. Miscellaneous Characters…………………………………………………… 82
Explanation of Data Supplements……………………………………………….. 84
Supplementary Figures……………………………………………………………… 86
Fig. S1. The cranial anatomy of *Tethymyxine tapirostrum*……………………………… 86
Fig. S2. The trunk anatomy of *Tethymyxine tapirostrum*……………………………….. 88
Fig. S3. The chemical compositions of *Tethymyxine tapirostrum* (BHI 6445)…………. 90
Fig. S4. Analyses of the output from SRS-XRF………………………………………… 92
Fig. S5. Comparative analysis of the morphological dataset……………………………. 93
Fig. S6. Cyclostome monophyly under parsimony……………………………………… 95
Fig. S7. Cyclostome monophyly under Bayesian inferences…………………………… 97
Fig. S8. Selected results of secondary analyses using alternative sampling strategies…. 99
Fig. S9. Time-scaled maximum clade-credibility tree………………………………….. 101
Supplementary Tables………………………………………………………………. 102
Table S1. Quantification of trace elements in the holotype of *Tethymyxine tapirostrum*.. 102
Table S2. Operational taxonomic units included in the dataset…………………………. 103
Table S3. List of synapomorphies for major nodes……………………………………... 105
Table S4. Comparison of character support for cyclostomes……………………………. 117
References for Supplementary Appendix…………………………………………….. 122

Part A. Additional Information on Holotype

A1. Taphonomy and Paleoecological Implications

Holotype of *Tethymyxine tapirostrum* (BHI 6445) is exquisitely preserved. The preservation of branchial pouches suggests the early stage of decay when buried (1, 2). In comparison to a decay series of a modern hagfish (*Myxine glutinosa*) under controlled conditions, the organs preserved in BHI 6445 include the intestine (onset of loss in *M. glutinosa*: 2 days post-mortem), slime glands (4 days), heart (6 days), branchial pouches (15 days), barbels and liver (48 days), myomeres and caudal fin (63 days), chondrocranium and keratinous tooth plates (200 days) (1, 2). Among them, the gut is preserved in BHI 6445, and the slime glands are represented by α-keratin infillings. Some other organs are not preserved, even though they are expected to be present in the slab of BHI 6445, including: nasopharyngeal duct, pharyngocutaneous duct, and mouth (4 days) and inflection of myomeres (11 days) (1, 2). On the basis of this combination, BHI 6445 may be best compared to decay stage 2 of modern hagfish, between the onset of loss of heart and of branchial pouches (1, 2).

The mode of preservation is similar to the specimens of *Mesomyzon mengae* from the Lower Cretaceous Jehol Group of China (3, 4). Compared to the modern river lamprey *Lampetra fluviatilis*, the organs prone to rapid disintegration but preserved in the specimens of *Mesomyzon* include: branchial cartilages (11 days), pericardiac cartilage (135 days), branchial lamellae, chondrocranium, and otic capsules (207 days) (1, 2). This combination would place the adult specimens of *Mesomyzon* between the decay stage 1 (loss of branchial cartilages) and 2 (loss of kidney) (1, 2). In specimens of *Mesomyzon*, the external organs such as the epidermis and myomeres are preserved so well that they obscure internal structures. Therefore, it is difficult to constrain the morphology of the skeletons and visceral tissues in this taxon. This is not the case in BHI 6445. Like in typical vertebrate fossils from the Cenomanian limestone of Hâdjula (5, 6), the soft integument is only discerned at the outline, perhaps within a decay halo, which allows delineation of internal structures. These observations based on decay sequences of modern relatives cannot be assumed to replicate the taphonomy of fossil taxa

precisely (7). However, these comparisons indicate: (***a***) the tissues that tend to be lost relatively early post-mortem are non-equivocally preserved in BHI 6445 (e.g., intestine, branchial pouches, keratin); and thus (***b***) taphonomic artifacts are unlikely to explain the presence of myxinoid synapomorphies (e.g., posteriorly displaced branchial pouches) in BHI 6445 or the absence of characters diagnostic to other known vertebrate lineages.

Paleoecological implications remain unclear for the occurrence of a hagfish in Hâdjula. The Cenomanian limestone of the locality has been interpreted as a mass-death assemblage of shallow, coastal, marine fauna of an inter-reef basin on the carbonate platform (5, 6, 8) — seemingly an unexpected environment in which to find a hagfish. Living hagfish generally inhabit deep (> 400 m), high-salinity (> 30 ppt), low-temperature (< 20 ºC) regimes, especially at low latitudes (9). However, they may transiently occupy benthic habitats shallower than 50 m (9). If similar ecological constraints applied to *Tethymyxine*, it would suggest that the type specimen of *Tethymyxine* does not represent a resident population in the Cenomanian of Hâdjula. However, it is difficult to compare the occurrence of *Tethymyxine* with the ecology of modern hagfish in the absence of precise paleoclimatic estimates or geochemical indicators for local environmental conditions.

A2. Provenance

Holotype of *Tethymyxine tapirostrum* (BHI 6445) was discovered in the Cenomanian strata (5, 6) of Hâdjula (34°08'01.0" N, 35°44'38.1" E), Lebanon during the sponsored collecting operation of Geoworld Group by Stefano Piccini, and acquired by Black Hills Institute of Geological Research in 2013. Numerous vertebrate, invertebrate, and plant fossils were collected from the Cenomanian limestone of Hâdjula, constituting a significant portion of the enormous collections in world-wide museum repositories of the Cenomanian reef communities of Lebanon (5, 6).

The collections at the Black Hills Institute have served as a depository of two other holotype specimens (10, 11). The Institute issued a corporate resolution in 1983 and reaffirmed it in 2014 — in

compliance with the bylaw of the Society of Vertebrate Paleontology — to state its public mission, including: (***a***) all type and referred specimens accessioned at the Institute shall remain accessible to the scientific community as a portion of the permanent collections; and (***b***) in the event of dissolution of the Institute, all such specimens shall be deposited in one or more of the seven designated, publicly funded museums in North America. Digital data from SRS-XRF have been accessioned at DOI: 10.6084/m9.figshare.7545002

Part B. METHODS

B1. SRS-XRF

SRS-XRF (12) of BHI 6445 was performed at beam line 6-2 at the Stanford Synchrotron Radiation Light-source (SSRL), SLAC National Accelerator Laboratory, USA. Experiments were conducted with an incident beam energy of either 13.5 keV (flux calculated between 10^{10} and 10^{11} photons s^{-1}) or 3.15 keV (flux ~10^{9} photons s^{-1}) and a beam diameter of 50 μm defined by a pinhole. Fluoresced x-rays were detected using a single element Vortex silicon drift detector. SRS-XRF maps from SSRL were processed from the raw detector count raster files using a custom MATLAB script that converted the data array into 8 bit tiff images clipped at various contrast percentiles. Image subtraction was performed using ImageJ (13), and the image correlation was completed using the CorrelationJ plugin.

Energy dispersive spectra for quantification were obtained from single spot locations (50 μm spot size) for 100 live seconds. All EDS spectra were fitted using PyMCA (14) from fundamental parameters of the experiment using a Durango apatite (fluoroapatite) mineral standard with known element concentrations for calibration. 2σ errors on concentration were calculated using the standard deviation of peak area for each element output by PyMCA. The concentrations of each element are an average of 3 individual EDS spectra taken within a few hundred microns of each other on the same specimen.

The results from SRS-XRF were analyzed primarily through spatial distribution of trace elements (Figs. S3, S4) (15). Several elements have similar concentrations in the fossil and the matrix. The Ca signal appears relatively reduced throughout the fossil tissue compared to the high Ca content of the embedding limestone matrix. Thus, absolute Ca contents may be high in the tissues, but it is difficult to contrast specific tissues against the background on the basis of distributions alone. In the case of S, the spectrum is clearly different between the preserved tissues and the glue that shows only inorganic S (Fig. S4). The inorganic S and other organic S species (with peak energies equivalent to theoretical values for methionine sulphoxide, sulphonate, and cysteine) in the matrix are oxidation products diffused away from BHI 6445. Higher S levels in the tissues relative to the matrix (Table S1) imply that mass transfer of S was from the fossil outwards. However, spectroscopy shows relatively high levels of organic S in the matrix as well, which is a function of the biogenic nature of the Hâdjula limestones. The XANES spectroscopy confirms that the organic S species present in both the matrix and fossil are different and requires further detailed study via this method to quantify the compositional variance.

The curatorial artifacts (glue and paint) can be easily identified in SRS-XRF, highlighting potential advantage of applying this method to fossils. An 'N-shaped' repair in the center of the specimen is highlighted by the glue, which showed elevated levels of Ti, V, Mn, and Fe. The distributions of Ni, Cu, Zn, P, S, As and Hg were predominantly controlled by soft tissues. These results are consistent with the observation that Cu, As, Fe and Hg naturally accumulate in the tissues of extant hagfish (16). In particular, hagfish are predisposed to hyperacumulate Hg. As for Ni, the living hagfish *Eptatretus stoutii* takes up this element via a high affinity-low capacity transport pathway, which mainly accumulates the element in the brain, gills, and heart (17). The high levels of P and S in BHI 6445 may be attributed to the presence of polysulphate peptides in the skin (18). Thus, the levels of these elements observed in BHI 6445 are expected for a myxinoid. Although the concentrations of Hg were below detection limits on a single point, the SRS-XRF maps indicate the presence of this

element. A cadaver decay island appears to surround BHI 6445. This decay halo may be due to the mass-transfer of elements from the organism to the embedding matrix. The fossil still has elevated levels of several elements (P, S, As, Cl, Zn and Cu) relative to the matrix. Fe distributions also indicate a small decay halo. Data from SRS-XRF are deposited at DOI: 10.6084/m9.figshare.7545002

B2. Phylogenetic Analyses

B2a. Overview

No previous phenotype-based cladistic analysis has supported cyclostome monophyly. Instead, a phenotype-based analysis tends to recover hagfish nested outside the lamprey+gnathostome clade (19–31). Although most of the previous analyses were of maximum parsimony, the topology is not an artifact of the algorithm. Bayesian approach still did not support monophyly of cyclostomes with morphological characters (32). It is not just an artifact of using the same dataset, either. Many of the recent analyses (26–28, 32, 33) used independently modified versions of the dataset constructed by Donoghue and colleagues (23), which was formulated after Janvier (21). There are several datasets constructed outside the mainstream parent-child datasets (29, 34–36). Still, none of them supported monophyletic cyclostomes. Although hagfish-lamprey comparison of selected characters questioned paraphyly (37–40), these comparative analyses often lacked proper outgroup comparison. Several analyses experimented with different taxon- and character-sampling schemes and topological constraint to compare cyclostome monophyly versus paraphyly (23, 26, 32). Paraphyly still persisted as the most parsimonious hypothesis. Nevertheless, the difference between cyclostome monophyly versus paraphyly is subtle in levels of statistical support (34, 41), which highlights the need to examine morphological characters and coding carefully.

A treatment of miRNAs as discrete characters (presence/absence) favors cyclostome monophyly over paraphyly, as hagfish and lampreys share at least four miRNA families unique to them (34). However, the phylogenetic utility of presence/absence of miRNA families has since been

questioned (42). In the analysis by Heimberg and colleagues (34), sampling errors were not considered because non-detection of expression in samples was interpreted as the absence of the miRNA family altogether. Furthermore, secondary loss of miRNA is common across the metazoan tree. Heimberg and colleagues (34) presented a tree in which all secondary losses of miRNA were mapped outside the stems of cyclostomes, gnathostomes, and vertebrates. The models incorporating sampling errors and secondary losses did not corroborate such miRNA-based trees (42). Therefore, cyclostome monophyly remains under question when the datasets contain standard presence/absence data.

In comparison to phenotypic data, molecular datasets have almost always supported monophyly of cyclostomes (41, 43–52). Cyclostome monophyly is a topology predicted for long branch attraction, so this is an expected result of a molecular-based analysis (41). The consistency of molecular inferences is not to be confused with additive reinforcement of the topology (41). Nevertheless, cyclostome monophyly has gained wide acceptance to the extent that recent phenotype-based analyses use monophyletic cyclostomes as a topological constraint (23, 28, 32, 33, 35). Such a constraint imposes serious theoretical implications to interpreting the resulting trees. For these reasons, cyclostome paraphyly has not been ruled out entirely (53).

For the new attempt including *Tethymyxine* in the dataset, both parsimony and Bayesian analyses were performed. The morphological dataset is common among all analyses. It contains 52 taxa (Table S2) and 168 characters (**Part E. List of Characters**). Rationales for taxon and character sampling are discussed in detail in **Part D. Character and Taxon Sampling**. The purpose of combining parsimony and Bayesian analyses is to identify robustly supported clades. Parsimony and probabilistic methods often result in trees of different shapes and resolutions even using the same morphological datasets (54, 55). The debate is ongoing about which method outperforms others, and which method should be favored (56–63). For our analyses, it is critical to avoid confusing those two questions. Bayesian methods tend to be more accurate than parsimony, but parsimony appears to be

more precise (greater resolution) (58, 59). Probabilistic methods require proper estimates of parameters or priors (54, 64), which remains challenging in cyclostomes.

The factors to consider for our dataset are: (***a***) it contains a large proportion of taxa that are highly incomplete (Table S2); (***b***) missing entries are distributed non-randomly and asymmetrically, and characters based on soft tissues are underrepresented; (***c***) we enhanced contingency coding for the current dataset; and (***d***) previous morphological datasets consistently supported cyclostomes as paraphyletic under parsimony. Precision is an important criterion when including a large number of incomplete and unstable taxa (***a***), hence parsimony may be preferred in this respect. Although biased distribution of missing entries (***b***) and historical trends (***d***) suggest probabilistic methods as a suitable option, the enhancement of contingency coding (***c***) questions that choice. How degrees of contingency in morphological datasets impact the performance of probabilistic methods remains unclear (54, 65). Under parsimony, however, contingency coding has predictable outcomes (66). Therefore, we used maximum parsimony as a primary line of phylogenetic analyses, and ran Bayesian analyses to highlight areas of agreement and disagreement with the results of the parsimony analysis.

B2b. Maximum parsimony analyses

For the parsimony analyses, we used PAUP* 4.0a152 (67) (heuristic search; 10,000 random sequence additions; TBR; outgroup: Hemichordata) with no topological constraint. The main analysis included the 52 core taxa (retained + new in Table S2).

B2c. Bayesian analyses

For Bayesian analyses, we used MrBayes ver.3.2.5 (68). Each analysis was run for ten million generations with four runs of eight chains in total, without topological constraint, and sampling every five thousand generations. To facilitate comparison of different taxonomic combinations, all MCMC searches with MrBayes used the same set of priors:

Datatype = Standard

Coding = Variable

States = Variable, up to 10

Symmetric Dirichlet is fixed to 2.00

Rates = Gamma

Alpha_symdir

Type = Symmetric diricihlet/beta distribution alpha_i parameter

Prior = Symmetric dirichlet with fixed (2.00) variance parameter

Alpha

Type = Shape of scaled gamma distribution of site rates

Prior = Exponential(1.00)

Ratemultiplier

Type = Partition-specific rate multiplier

Prior = Dirichlet(1.00)

Tau

Type = Topology

Prior = All topologies equally probable a priori

Subparam. = V

V

Type = Branch lengths

Prior = Unconstrained: GammaDir (1.0, 0.1000, 1.0, 1.0)

Relative burnin = 25.0%

In addition, we used tip-dated molecular clock to calibrate nodes in the tree presented in Fig. S6. Fossilized Birth Death model was run in BEAST 2.4.5 (69) (gamma site model: HKY,

kappa = 2.0; clock model: relaxed clock, log normal). The dataset for molecular clock includes 57 taxa with 1493 loci from 16S and COI genes (Supplementary data file 4). Fossil taxa were used as hard minimum calibration points. Tree topology was constrained after strict consensus of the maximum parsimony analysis (Fig. S6) using tree priors. Some of these nodes were given strong priors on the basis of (***a***) consensus of molecular clock estimates and (***b***) other constraints (e.g., Northern and Southern Hemispheric clades of lampreys were encouraged to diverge from one another before the complete separation of Laurasia and Gondwana). The BEAST analysis was run over 10 million generations, and MCC tree was selected for median heights.

Part C. RESULTS OF PHYLOGENETIC ANALYSES

C1. Maximum Parsimony Analyses

In the analysis of 52 taxa (Table S2), heuristic search found 16 most parsimonious trees (tree length = 366; consistency index = 0.519; retention index = 0.798; rescaled consistency index = 0.414; homoplasy index = 0.481) (Fig. S6). The strict consensus tree resolves *Tethymyxine* as nested within the crown-group Myxinoidea with the species of *Rubicundus*. Cyclostomes (myxinoids + petromyzontiforms) were recovered as a clade. Anaspids, euconodonts, and *Pipiscius* were nested outside the crown (split between myxinoids and petromyzontiforms). The relationships among myxinoids closely follow those from the previous Bayesian analysis of living myxinoids using COI gene (70): (***a***) *Rubicundus* was nested outside other living myxinoids; (***b***) *Neomyxine* and *Myxine* formed a clade; and (***c***) *Paramyxine* was nested among *Eptatretus*. *Myxinikela* (71) was resolved as a stem myxinoid, whereas *Gilpichthys* and *Myxineidus* — two forms previously posited as potential stem hagfish — were placed among stem petromyzontiforms. In that latter clade, the Northern and Southern Hemispheric clades were recovered in accord with both morphological and molecular inferences (72–76). The chronologically oldest stem petromyzontiform *Priscomyzon* was closer to the crown than the

Carboniferous *Hardiestilla* and *Mayomyzon*. *Mesomyzon* did not fall into Northern or Southern Hemispheric clades. In this consensus, crown-group cyclostomes are united by two unambiguous character changes (142.1: keratinous feeding structure; 163.1: postotic myomeres assuming periotic position) (Table S3).

Cephalochordates and *Pikaia* were nested outside the Olfactores (tunciates + vertebrates). The controversial Cambrian forms were recovered in the vertebrate stem. Myllokunmingiids (*Haikouichthys* + *Myllokunmingia*) formed a clade, whereas *Metaspriggina* is the closest and *Haikouella* farthest from the crown vertebrate node. Crown-group vertebrates are split between the total groups of cyclostomes and gnathostomes (both monophyletic). In the gnathostome clade, the consensus tree closely follows topologies previously recovered, except about the stem cyclostome status of anaspids (23, 26–28, 32, 33, 35, 36).

The position and membership of the anaspid clade is unconventional in this analysis. Anaspids have been placed on the stem of gnathostomes in most previous analyses (23, 26–28, 32, 33, 35, 36). In this analysis, euphaneropids — or 'naked' anaspids — (*Euphanerops* and *Jamoytius*) are nested within the clade of 'armoured' anaspids (*Birkenia*, *Rhyncholepis*, and *Lasanius*). *Achanarella*, *Ciderius*, and *Cornovichthys* were often compared with euphaneropids anatomically but found in a polytomy at the base of vertebrates in the previous analysis (32). These potential euphaneropids form outgroups of that clade. It takes five additional steps to move the clade (preserving all other topologies) to either the stem of gnathostomes or that of vertebrates. However, it is two steps away to have one or more of these anaspid taxa outside cyclostomes. This instability is expected given that many of these taxa are incompletely preserved. The stem cyclostome status of euconodonts is consistent with some of the recent analyses (28, 33).

The crown cyclostome node is supported by two unambiguous character changes: keratinous feeding structures (character #141:1) and periocular position of trunk myomeres (161:1) (Table S3). Other potential synapomorphies include: fusion of profundal and trigeminal ganglia (6:1); flattened

profile of spinal cord (8:1); deposition of calcium phosphates in inner ear (37:1); lingual apparatus (142:1); cartilaginous trematic rings (147:1); the absence of vertebral centra (150:0); and intracoelomic sperm dispensation (160:1). Information is generally missing for these characters in fossil taxa (Table S3) so these changes are constrained to the crown cyclostome node under DELTRAN optimization. Mature chondrocytes that are hypertrophied to the diameter of approximately 50 µm (101:1) and organized in pairs (102:1) unite myxinoids and petromyzontiforms, but these two states also occur in *Euphanerops* (77). With *Euphanerops* nested among anaspids on the cyclostome stem, these two characters are constrained to the total cyclostome node (Table S3).

C2. Bayesian Inferences

MCMC search of the morphological dataset including 52 core taxa (retained + new; Table S2) by MrBayes 3.2.5 resulted in a consensus tree that is remarkably consistent with the parsimony-based consensus (Fig. S7). It found majority support for the cyclostome total group and crown group. As in the maximum parsimony analyses, *Tethymyxine* and *Rubicundus* were nested outside the rest of crown myxinoids, where *Tethymyxine* is sister to *Rubicundus* spp. *Myxinikela* sat in the stem of myxinoids, whereas euconodonts had marginal HD support (59%) as a stem myxinoid lineage. Likewise, *Gilpichthys* migrated to the myxinoid stem. The crown petromyzontiform node had less than 50% HPD support — thus in a polytomy with *Myxineidus*, *Priscomyzon*, and *Mesomyzon*. The crown cyclostome node is at a polytomy with the Carboniferous forms *Hardistiella*, *Mayomyzon*, and *Pipiscius*. Anaspids were resolved in a grade, with mineralized forms closest to the crown vertebrate node, with naked forms closest to the crown cyclostome node, and with *Lasanius* in between. The crown vertebrate node was collapsed into a polytomy with several 'ostracoderm' lineages (arandaspids, astraspids, *Athenaegis*, and heterostracans). Outside the crown vertebrate node, *Haikouella* remains the closest to the crown olfactorian node, whereas myllokunmingiids — not *Metaspriggina* — are the most immediate outgroup to the crown group of vertebrates.

C3. Morphological Support for Cyclostome Monophyly

Cyclostome monophyly has never been supported parsimoniously by morphological data before this analysis. This topological change is attributed to enhancement of contingency coding and maximum inclusion of putative fossil cyclostomes (both detailed in **Part D. Character and Taxon Sampling**). When the matrix was re-scored without considering character contingency, cyclostomes formed a grade where myxinoids ((myxinoid crown + euconodonts) + *Gilpichthys*) became nested outside the clade of petromyzontiforms and gnathostomes under strict consensus of 3198 most parsimonious trees (Fig. S8a). This is a classical position of myxinoids in the previous phenotype-based cladistic analyses.

Under contingency coding, the consensus trees from the parsimony and Bayesian analyses (Figs. S6 and S7) corroborated phylogenetic inferences based on molecular data. In addition, they provide topological resolution for some controversial fossil taxa. Unexpectedly, unambiguous character changes at the total and crown nodes of Cyclostomi did not include many structures considered synapomorphic to cyclostomes such as lingual apparatus and velum (37, 38) unless optimized under ACCTRAN (Table S3: Cyclostomi). These structures have relatively low preservation potentials in fossils (1, 2, 78), and the characters are uninformative to parsimony as they cannot be compared outside cyclostomes (discussed in **Part D. Character and Taxon Sampling**). The contingency coding ruled most characters related to biomineralization inapplicable among crown cyclostomes, and redefined characters previously considered simply absent in hagfish. Thus it prevented myxinoids from slipping stemward, and the presence of several characters with relatively high consistency index (e.g., 161:1: anterior shift of postotic myomeres; CI=1.00) united them with petromyzontiforms.

The stem cyclostome status of anaspids and euconodonts implies that living cyclostomes lost the ability to mineralize their skeletons secondarily. When character contingency was not considered (thus biomineralization was implicitly weighted), these topologies were not recovered (Fig. S8a). Anaspids have mineralized integumentary skeleton (calcified endoskeleton in *Euphanerops*), whereas

euconodonts have mineralized feeding apparatus. At the level of trait expression, either biomineralization evolved three times (once each in anaspids, euconodonts, and gnathostomes) or became lost independently in crown-group cyclostomes and *Pipiscius*, and within anaspids — if not combination of both. It is difficult to compare these taxa because different skeletal elements are mineralized. Rather than optimizing ancestral state reconstruction methods for simplistic presence/absence of mineralization, we point out that the presence of mineralized skeletons in stem cyclostomes is consistent with two observations: (***a***) living cyclostomes are capable of depositing matrix of calcium phosphate (present as lining of inner ear) (79); and (***b***) cultured chondrocytes of living lampreys undergo calcification (80). If this line of inference is correct, the innate potential to mineralize skeletal matrix may have existed as a crown-vertebrate trait.

C4. Additional Analyses Using Different Combinations of Characters

C4a. Analysis of characters for crown cyclostomes

The parsimony and Bayesian analyses suggest *Tethymyxine* is a crown myxinoid, sister to *Rubicundus*. Under maximum parsimony, three unambiguous changes unite these genera at a node: tapering nasohypophyseal profile (21:1); nasohypophyseal barbels originating posterior to aperture (23:1); and branchial openings spaced accordingly with branchial pouches (55:1) (Table S3: *Tethymyxine* + *Rubicundus*). Character 55 cannot be scored for *Tethymyxine*, whereas characters 21 and 23 allow multiple interpretations. The nasohypophyseal morphology is difficult to assess in a compressed fossil. Possibly, the nasal cartilages could have dislocated and extended past the barbels. Assuming such taphonomic artifacts, unknown (‘?’) would be an alternative scoring for *Tethymyxine* in characters 21-23. After running a maximum parsimony analysis of the same dataset under this alternative coding, the *Tethymyxine* + *Rubicundus* node became fully resolved within crown myxinoids, with *R. lopheliae* nested outside the clade *Tethymyxine* + *R. eos*. The latter clade is supported by a single character (167.1: slime glands >100), as the number of slime glands varies within *Rubicundus* (X in Table S3:

Tethymyxine+*Rubicundus*). Thus, this secondary analysis resulted in the almost identical topology presented in Fig. S6.

It still remains possible that the barbels were misidentified. Anatomically, the connection with the anterior tentacular cartilage (Fig. S1) makes a compelling case that the identified barbels are real. Postulating that the barbels were entirely misidentified nevertheless, characters 21-23, 157 (barbels supported by cartilage), 158 (forked subnasal cartilage, supporting the lower nasohypophsyeal barbels) were modified to unknown ('?') in *Tethymyxine*. Under maximum parsimony, this secondary analysis of the modified dataset still resulted in strict consensus identical to the topology in which nasohypophyseal characters were modified to unknown. Therefore, the position of *Tethymyxine* sister to or nested among *Rubicundus* was supported regardless of interpretations of the nasohypophyseal morphology in BHI 6445.

Among other soft tissues preserved in BHI 6445, the tooth plates and slime glands are diagnostic to myxinoids (Table S3). These structures were identified on the basis of their positions, morphology, and geochemical evidence (Figs. S2-S4). Accepting their identities, the number of slime glands (character 167) may be subject to interpretation. Changing the score of this character in *Tethymyxine* to unknown (while keeping characters 21-23, 157, 158 as unknown) collapsed the *Tethymyxine* + *Rubicundus* clade to the crown myxinoid node (Fig. S8b). Adding on to these modifications to explore alternative character coding further, a rejection of geochemical signals for keratin in the identified structures would lead to two more changes in the character scores of *Tethymyxine*: character 141 (keratinous tooth plates) and 142 (lingual apparatus). Coding these characters as unknown in *Tethymyxine* did not impact the topology. *Tethymyxine* was still resolved at the crown myxinoid node. Therefore, character support is robust for the crown myxinoid status of *Tethymyxine*.

Character changes supporting the crown cyclostome node are either (***a***) umambiguous for the characters that can be scored in euconodonts (characters 141 and 161) or (***b***) optimized under

DELTRAN for characters that are largely unknown in fossils (Table S3: Cyclostomi, crown group). Myomeres (161:1) and keratinous feeding structures (141:1) are resistant to decay relative to other soft tissues (1, 2) so they have reasonable chance of being scored for fossils from a locality where these tissues are known to be preserved. Other characters that may be preserved in fossils include the presence of multidenticulate/cuspidate elements (135:1) and lingual apparatus (142:1), and the absence of arcualia around the notochord (150:0). Together with character changes associated with the total-group node (Table S3), no particular character partitions appear to predominate in supporting the clade of cyclostomes.

C4b. Analysis of characters for conodonts

Several characters scored for conodonts may be interpreted differently (detailed discussion in **Part E. List of Characters**). These include extraocular muscles (28:?), position of branchial apparatus (45:-), number of branchial pouches (56:?), lingual apparatus (142:?), and anterior extreme of myomeres (161:0). We coded these characters for conodonts conservatively because they are based on soft tissues, and because interpretations are polarized between authors. For character 28, the tissue in question may be extraocular muscles or mucocartilage-like structures (**Part E. List of Characters**). We accept the putative branchial pouches in *Clydagnathus* (23, 81). However, the number may be greater than the preserved five (character 56) and appropriate landmark structures are either absent or not preserved in the specimen (character 45).

Conodont apparati were once compared to hagfish tooth plates based on detection of amelogenin in the latter (82). No morphological evidence was presented, and a histological comparison reveals no similarity between the two structures (83). A spectroscopic analysis recently suggested keratinous residues in conodont elements (84). However, nothing is known about morphology of the structure that consisted of keratin. Therefore, the character for keratinous teeth is coded as absent in conodonts conservatively (141:0). Conodont feeding mechanics has been compared to the lingual

apparatus of living cyclostomes (85, 86). In the absence of evidence for skeletal and muscular components of the lingual apparatus, however, this comparison could be interpreted as an implicit resurrection of a comparison between conodont elements and hagfish keratinous teeth.

For interpretations of the preserved structures that were questioned originally in this analysis, reversing these character scores (28:1, 45:0, 56:0, 141:?, 161:?) moved euconodonts and *Gilpichthys* to the myxinoid stem (Fig. S8c). Under strict consensus of 80 trees, the crown cyclostome node is collapsed into a polytomy with *Hardistiella* and *Mayomyzon*.

Although euconodonts were coded conservatively overall, some characters were interpreted provisionally in this lineage. These include an upper lip being absent (66:0) and preanal skin fold being absent (90:0). Changing these character scores to unknown ('?') while keeping the alternate character coding (28:1, 45:0, 56:0, 141:?, 161:?) did not alter topology (Fig. S8c). Under this scheme, euconodonts and *Gilpichthys* remained on the myxinoid stem. Therefore, any particular assessment of individual character scores does not seem to impact the topology of euconodonts greatly.

C4c. Analysis of characters for Pipiscius

Pipiscius is another taxon that we coded conservatively. Although consistently reconstructed as a lamprey-like animal (87–90), an alternative view compared the taxon with *Xidazoon*, a Cambrian vetulicolian (91). A survey of the list of synapomorphies (Table S3) reveals that (***a***) *Pipiscisus* is coded as unknown or inapplicable for many characters diagnostic to cyclostomes; and (***b***) the character scores do not agree with the states shared in crown cyclostomes or petromyzontiforms. The character scores presented here reflect a skeptical view of some traits in *Pipiscius* that have been interpreted as lamprey-like, including a perioral ring of cusps and a lingual apparatus.

To explore the position of *Pipiscius*, the following characters merit reevaluation: keratinous perioral/buccal plates (141:?), lingual apparatus (142:0), radially organized circumoral plates (145:?), and annular cartilage (155:?). Geochemical comparison of the circumoral 'teeth' of *Pipiscius* (36) did

not have a control that is unequivocal keratin, so it remains unclear whether the elements in question are truly keratinous or not. The cusps are not organized as in lampreys (discussed in character 145; **Part E. List of Characters**) and their inward orientations would not allow a pulley-like eversion of oral feeding structures as in the lingual apparatus of modern cyclostomes (discussed in character 142; **Part E. List of Characters**). Annular cartilage may have been present to support the circumoral cuspidate plates, but the cartilage is generally resistant to decay (2). Indeed, it is preserved in the smaller coeval form *Mayomyzon*, which has less prominent an oral funnel than that in *Pipiscius*.

Still interpreting these characters in *Pipiscius* in light of living lampreys, revised coding (141:1, 142:?, 145:1, 146:2, 155:1) resulted in 90036 most parsimonious trees. Strict consensus of these trees collapsed all internal nodes within petromyzontiforms (except the clade of *Geotria* and *Mordacia*), with *Pipiscius* nested in that polytomy (Fig. S8d). *Gilpichthys* was resolved in a polytomy with myxinoids and petromyzontiforms at the crown cyclostome node. In anaspids, 'naked' anaspids formed a clade to which an immediate outgroup is *Lasanius*, followed by the clade of *Birkenia* and *Rhyncholepis*. This secondary analysis predict that further evidence for the characters coded conservatively in our analyses would resolve *Pipiscius* as a stem petromyzontiform.

C4d. Analysis of characters for Gilpichthys

The petromyzonform position of *Gilpichthys* (Fig. S6) is also intriguing, as this taxon has always been compared to hagfish (87–89). However, the myxinoid affinity is based on the lack of lamprey-like characters and not on the presence of characters diagnostic to myxinoids (Table S3). The parallel anterior structures were interpreted as keratinous tooth plates (141:1), but this occurs among petromyzontiforms. In addition, *Gilpichthys* can be scored for only a small proportion of characters (22.6%). Therefore, the position is more sensitive to the inclusion/exclusion of the characters coded for *Gilpichthys* than to interpretations of the characters preserved.

C4e. Analysis of characters for euphaneropids

Similar arguments can be made about *Achanarella*, *Ciderius*, and *Cornovichthys*. These taxa contain missing or inapplicable entries for 76.2-79.8% of characters. We provide justification for individual character scoring in **Part E. List of Characters**. As for the first three (Table S3), character states are similar to *Euphanerops* and *Jamoytius* for unambiguous and optimized synapomorphies at the major nodes (Table S3). Recently, an assessment of taphonomy at the type localities suggested *Achanarella* and *Cornovichthys* as different ontogenetic stages of an euphaneropid (92). Merging these two taxa, strict consensus of the most parsimonious trees (n=28435) collapsed all internal nodes within anaspids except for the clade of the composite euphaneropid and *Ciderius*, and most internal nodes of petromyzontiforms to collapse into a polytomy at the crown cyclostome node (Fig. S8e). When *Achanarella*, *Ciderius*, and *Cornovichthys* were deleted from the dataset, other anaspid taxa were nested in a polytomy (Fig. S8f).

The overall morphology suggests that *Achanarella*, *Ciderius*, and *Cornovichthys* are closely related to *Euphanerops* and perhaps to *Jamoytius*. Nested outside the clade birkeniids + euphaneropids (Fig. S6), their positions in strict consensus of parsimonious trees are compatible for each representing an immature ontogenetic stage. It is also likely that euphaneropid specimens represent varying stages of decay (92). However, they were treated as distinct taxa in this analysis because ontogeny or taphonomy does not fully explain morphological differences. *Achanarella* and *Cornovichthys* differ in states for characters 57 (number of branchial arches), 84 (distinct dorsal fin), and 87 (distinct anal fin). Either or both of these taxa differ from *Euphanerops* in states for characters 45 (arrangement of branchial apparatus), 57, 84, 87, and 95 (skeleton of calcium phosphates).

Given the size of individual arch-like structure relative to the entire branchial frame, the number of branchial arches is likely greater in *Achanarella* than in *Cornovichthys*. The presence of dorsal fin in *Achanarella* is open to interpretation. The structure identified as a dorsal fin in this animal may represent displaced myomeres. A prominent ventral fin in *Cornovichthys* may represent an anal fin.

However, the gut trace above this fin suggests that the anus was behind the ventral fin. The gut could have looped back into an anus anterior to the fin, but no reasonable explanation exists to postulate such a peculiar morphology. Changing these character scores (84:0 for *Achanarella*; 87:1, 90:0, 91:-, 92:- for *Cornovichthys*), all internal nodes were collapsed for anaspids (with additional polytomies in cyclostomes). To follow up this analysis, the presence/absence of calcium phosphates in skeleton was further modified to ambiguous for *Achanarella*, *Ciderius*, and *Cornovichthys* (95:?) while retaining modified codings for the fin characters. This modification is based on two lines of observation: (***a***) larval or juvenile specimens of *Euphanerops* from the Miguasha Lagerstätte have no clear indication of calcified cartilages at sizes similar to *Achanarella*; and (***b***) given the state of preservation, an incipient or incompletely mineralized endoskeleton could have gone undetected for these putative euphaneropids from the Silurian/Devonian Scotland. This last analysis resolved the anaspid polytomy partially by recovering euphaneropids (*Achanarella*, *Ciderius*, *Cornovichthys*, *Euphanerops*, and *Jamoytius*) and another naked anaspid (*Lasanius*) in a clade.

C4f. Analysis of characters for Cambrian taxa

Putative Cambrian stem vertebrates (*Haikouella*, *Haikouichthys*, *Metaspriggina*, *Myllokunmingia*) share several important characters with crown vertebrates, including the well-developed sensory capsules, overall head configurations, and pharyngeal and axial skeletons (Table S3). They consistently lack most other diagnostic character states at the internal nodes within the crown group of vertebrates, or are not preserved with them (Table S3). One exception is *Haikouella*, which was coded as lacking neural crest-derived skeleton (1:0) and endoskeletal fin supports (83:0). *Haikouichthys* and *Metaspriggina* have skeletal bars in the pharynx, but were not coded for neural crest-derived skeletons (1:?) in the absence of other correlates of neural crest (e.g., 3:1, distinct prechordal head) and with the mesodermally derived pharyngeal cartilages in cephalochordates (93). The pharyngeal region of *Myllokunmingia* is not preserved well enough to assess this character. *Haikouella* — coeval with

Haikouichthys and known from numerous well-preserved specimens (94) — still has no evidence for these elements, or any other skeletal elements known to be derived from neural crest in modern vertebrates. The character 'fin supports' was coded following the same logic. Changing these character scores in *Haikouella* to unknown (1:?, 83:?) resulted in an identical topology of strict consensus tree with Fig. S6.

C4g. Questionable myxinoid affinity of Palaeospondylus

Palaeospondylus was only included in secondary analyses. This problematic taxon has been posited as a stem myxinoid, stem petromyzontiform, jawless stem gnathostome, placoderm, chondrichthyan, teleost, or dipnoan/amphibian larva — but no hypothesis has been tested in a rigorous cladistic analysis (95–99). A recent reconstruction of *Palaeospondylus* as a stem myxinoid (95) is certainly provocative but contains some internal inconsistencies (100). The comparison is tenuous for some of the characters, such as: (***a***) the purported nasal capsule basket fused to the main palatal element; (***b***) the purported velar cartilages as mineralized, displaced elements; (***c***) the purported lingual apparatus being a bilaterally paired structure; and (***d***) otic capsule, which has three semicircular canals like jawed gnathostomes (100, 101). Like many other problematic taxa, even the coarsest classification is difficult because *Palaeospondylus* does not exhibit multiple characters that clearly falsify alternatives. As such, we used two coding schemes. (A) The cyclostome model was coded on the assumption that *Palaeospondylus* was a cyclostome (but not necessarily a myxinoid; excluding internal inconsistencies from character coding). (B) The gnathostome model was coded on the assumption that it was a gnathostome (but not necessarily jawless or jawed, nor specifically as placoderm, chondrichthyan, actinopterygian, or dipnoan).

When *Palaeospondylus* was coded on the assumption that it is a cyclostome, internal nodes of gnathostomes were collapsed into a polytomy at the crown vertebrate node (Fig. S8g). Other 'ostracoderms' were found in the cyclostome stem. *Palaeospondylus* was nested within anaspids in a

polytomy with *Euphanerops*. When *Palaeospondylus* was coded on the assumption that it is a gnathostome, the taxon was still found as a sister taxon to *Euphanerops*. Under strict consensus, anaspids migrated to the gnathostome stem as a basal grade, whereas cyclostomes formed a clade (Fig. S8h).

However, *Euphanerops* and *Palaeopondylus* are disparate from each other in suites of characters except for the combination of two characters (101:1, 102:1) — which code for large size and paired organization of chondrocytes. So these trees alone do not support or reject any of the multiple hypotheses proposed for the affinity of *Palaeospondylus*. In the very least, the quantitative analyses failed to corroborate the myxinoid hypothesis.

C4h. Exclusion of Tullimonstrum

Tullimonstrum is another problematic taxon recently reconstructed as a stem petromyzontiform (36) or as a vertebrate (102). For this taxon, a reanalysis questioned its membership as a petromyzontiform and the coding responsible for the placement (103). That reanalysis highlights the need for critical evaluation of the petromyzontiform or vertebrate hypotheses of *Tullimonstrum* against its alternative positions in protostomes (such as arthropods and molluscs). Soft tissue traits in *Tullimunstrum* have been interpreted variably to align with its proposed affinity, but rarely has evidence been presented to falsify particular hypotheses. Characters used to support the vertebrate or petromyzontiform affinity (36) can be interpreted differently (100, 103). These include those interpreted as arcualia, myomeres, or branchial pouches, proposed organization of the feeding apparatus, and number and arrangement of the structures interpreted as tectal cartilages.

Coding *Tullimonstrum* in our dataset similarly to the scheme B of Sallan and colleagues (103) (see Supplementary Data File 3), our preliminary parsimony analysis found it on the petromyzontiform stem, collapsing the total petromyzontiform node with *Gilpichthys*. This analysis did not test sensitivity of the character coding or sampling. Rather than supporting the petromyzontiform or vertebrate

affinity, the preliminary result highlights the degree of care that it must take to set a comparative framework for such a puzzling animal. A hypothesis cannot be falsified with a dataset sampling only one of the potential groups, and a resolved topology does not justify that choice of comparative framework. At this time, no single cladistic dataset available can test the full range of possible affinities for *Tullimonstrum*. So the taxon is not considered further in this paper.

C4i. Concluding statements for secondary analyses

Based on a number of primary and secondary analyses, the topology presented in Fig. S6 is robust to different taxon and character sampling methods. Stable nodes include the total/crown cyclostomes, total/crown myxinoids, and total/crown petromyzontiforms. Euconodonts represent a stem cyclostome lineage. *Tethymyxine* is a crown myxinoid. *Myxinikela* is a stem myxinoid, whereas *Gilpichthys* and *Myxineidu*s — often assigned to the same stem (21, 87–89, 104) — fell into the stem of petromyzontiforms. Putative Cambrian vertebrates are stem vertebrates. *Pipiscius* may represent a stem petromyzontiform.

The fossil taxa tended to be coded conservatively in this analysis. However, modifications to individual character scores did not impact the overall topology significantly. A review of justifications for the chosen character scores indicates that no one measure fits all when multiple conflicting interpretations exist for the characters. Sometimes one interpretation was favored, whereas other times the character coding was compromised into equivocal state as unknown or inapplicable. One taxon may be coded using different standards of evidence from one character to another, and standards of evidence may differ from a taxon to another in the same character. These decisions were made on case-by-case basis, and are discussed in individual character descriptions (**Part E. List of Characters**).

Part D. CHARACTER AND TAXON SAMPLING

D1. Sampling Strategies, Overview

The character list and data matrix used for this study were mainly derived from previous analyses (19–21, 23–29, 32–36, 105). Primary purposes of our analysis are to resolve the phylogenetic position of *Tethymyxine* and to test cyclostome relationships. Secondarily, those of putative stem cyclostomes and stem vertebrates may be affected by addition of new characters and taxa or re-interpretation of the existing characters. With these intentions, we constructed the dataset to (***a***) synthesize datasets from the recent analyses (32, 33, 35, 36) in character and taxon sampling and (***b***) increase character and taxon sampling as necessary.

For characters, the suite from Conway Morris and Caron (35) is used as a backbone, as this is the most recent extensive revision of the dataset by Donoghue and colleagues (23). The dataset by Conway Morris and Caron (35) (=CMC) received sequential revisions by McCoy and colleagues (36) (=MSL) and Gabbott and colleagues (32) (=GDS). Both expanded the taxonomic scope. Gabbott and colleagues (32) eliminated some characters and re-coded many others. Meanwhile, Keating and Donoghue (33) (=KD) presented another parallel revision to incorporate additional histological information. All characters included in the CMC, MSL, GDS, and/or KD datasets were considered. As a general rule, coding in this dataset reflects the latest scoring so that the GDS and KD coding supersedes the MSL or CMC wherever they disagree. We supplemented this initial dataset with (***a***) characters from other past analyses (27, 29, 34) and (***b***) wholly new characters. Each character in the list is denoted by the latest authority that defined it.

We edited the compiled character list extensively to: (1) revise character definitions; (2) revise character coding; and (3) eliminate parsimony-uninformative characters such that at least two states provide grouping information within a character. Each of these decisions is accounted for in **4.3 Character Sampling** and in the notes added to each modified character description (**Part E. List of Characters**: these characters are indicated as **definition modified** or **coding modified**). Most revisions

to character definitions and coding were made to reorganize hierarchically related character states through non-additive binary coding and contingency coding (66). The CMC dataset (35) and its subsequent revisions (32, 33, 36) already consist mostly of binary characters and contain contingency coding for 6-9% of all scores (Fig. S5A). The GDS dataset extended this approach by identifying additional characters that are hierarchical (e.g., arrangement of branchial openings [character 55] contingent on absence of single confluent branchial opening [character 53]) (32). However, it remains unclear how much of the contingency coding is carried over from the previous CMC and MSL datasets because the GDS matrix does not distinguish inapplicable scores due to contingency coding ('-') from missing entries due to lack of information ('?') (Fig. S5A).

Following these previous efforts, we enhanced the coding strategies (1-3) further. An enforcement of the reductive and contingency coding strategies is necessary as phenotype-based cladistic analysis of cyclostomes and gnathostomes has been criticized for favoring absence of derived states over loss or degeneracy of characters (22, 34, 37, 41, 88, 89, 106–108).

We followed the GDS analysis in using maximum parsimony as a primary method of phylogenetic reconstruction and using Bayesian analysis as secondary. Parsimony has predictable outcomes for optimization of non-binary additive and contingency coding strategies (66). On the other hand, these coding methods may affect Bayesian inferences computationally, but precise impacts are unknown. Although parsimony-uninformative characters are known to contribute to Bayesian inferences, such characters were still eliminated from the dataset to simplify the analytical pipeline, because: (***a***) non-additive and contingency coding strategies would require re-coding the existing characters that are contingent upon or related to the uninformative characters added back in; and (***b***) it is beyond the scope of the present study to sample unique character states exhaustively among these deep chordate lineages, at which point the character sets will no longer be reciprocal between parsimony and Bayesian analyses. Therefore, we optimized the dataset for a maximum parsimony analysis first to generate the shortest primary phylogenetic hypothesis, and then subjected the same

dataset to a Bayesian analysis to test for congruence and robustness of phylogenetic inferences supported by parsimony.

The resulting dataset contains 52 taxa and 168 characters (Tables S2; **Part E. List of Characters**). Among the characters, 54 represent new additions (not present in the CMC, MSL, GDS, and KD datasets (32, 33, 35, 36); 55 have modified definitions and coding from the latest datasets (32, 33); and 38 have same definition but modified coding. When coding for presence/absence of non-mineralized structures, we considered the non-random decay patterns in living analogues (1, 2, 78, 109) and locality-specific trends in taphonomic bias (26, 28, 32, 103) to reduce stemward slippage of soft-bodied taxa (78, 109, 110), conforming the analytical strategy in the recent analyses.

Abbreviations for previous datasets: CMC, Conway Morris and Caron (2014); **GCR**, Gess et al. (2006); **GDS**, Gabbott et al. (2016); **HSM**, Heimberg et al. (2010); **KLV**, Khonsari et al. (2009); **KD**, Keating and Donoghue (2016); **MH**, Mallatt and Holland (2013); **MSL**, McCoy et al. (2016).

D2. Excluded Characters

Characters were excluded on the following basis: (***a***) definition is unclear; (***b***) character is parsimony-uninformative, or does not contribute to resolving hagfishes or lampreys (if each was treated as a natural clade) in a parsimony analysis; and (***c***) characters duplicate the same phenotypic variations. Characters in category (***b***) may have been typically coded in previous analyses as primitive ('0)' for chordates and hagfish, and derived ('1') for lampreys and gnathostomes. Instead, many of these characters were coded for contingency as inapplicable ('-') for chordates, 0 for hagfish, and 1 for lampreys and gnathostomes. In the latter coding scheme, the character does not contribute to resolving cyclostomes parsimoniously as a clade or a grade.

CMC (Conway Morris and Caron, 2014) #5 (adenohypophysis, simple versus complex): definitions are unclear. This character is parsimony-uninformative unless coded for invertebrate chordates, which lack the system at anatomical levels.

CMC #6 (optic tectum, absent or present): the character is parsimony-uninformative. Vertebrate outgroups have neither camera eyes nor morphologically unequivocal brain compartmentalization. Therefore, the outgroups would be coded as inapplicable, whereas all living vertebrate taxa would be scored as present. This distribution does not inform cyclostomes.

CMC #20 (semicircular canals, absent or present): cannot be coded for the outgroups that lack an otic capsule (=parsimony-uninformative).

CMC #33 (endodermal branchial lamellae, absent or present): parsimony-uninformative when coding for hagfish is revised from absent to present.

CMC #34 (branchial lamellae with filaments, absent or present): parsimony-uninformative when the taxa lacking branchial lamellae are coded as inapplicable for this character.

CMC #36 (oral hood, absent or present): this character is redundant with the oral funnel/disc after re-coding. Cephalochordates do not have an oral hood readily comparable to lampreys. The prominent hood-like snout in lampreys arises from the posthypophyseal process anterior to the nasohypophyseal canal. The developmental attribute of this morphology is described in another character (#66, this analysis).

CMC #63 (midline retractor muscle and paired protoractor muscles, absent or present): the definition does not provide accurate description of the morphology. This character applies to both hagfish and lampreys (although the former was originally coded as absent), but in both lineages there are multiple retractors (38, 111). The character is inapplicable to those without a cyclostome-like lingual apparatus because the ‘protractors’ and ‘retractors’ cannot be compared with any other oropharyngeal structures outside cyclostomes. Once they are coded as such, the character is invariable.

CMC #66 (chondroitin 6-sulphate, absent or present): parsimony-uninformative when re-coded.

CMC #68 (neurocranium entirely closed dorsally, absent or present): parsimony-uninformative when re-coded. In the extant gnathostomes, the neurocranium is closed by: (***a***) dermal skull roof

(cranial vault; derived from neural crest and mesoderm in various combinations) and/or (***b***) extension of chondral elements (occipitals). These two states cannot be confused.

CMC #85 (scales/denticles/teeth composed of odontodes, absent or present): this character is constant after re-coding. Odontodes assume mineralized dermal skeleton, dentine, and pulp cavity, but these attributes are already described in the existing characters. Therefore, the taxa lacking dentine or mineralized dermal skeleton altogether cannot be scored for this character and are inapplicable. All known taxa with mineralized dermal skeleton and dentine have odontodes by definition (coded as present). So no taxon can be coded as absent. In this analysis, character #115 describes monodontodes versus polyodontodes as contingent on the presence of dentine (and therefore of odontodes).

CMC #96 (hyperosmoregulation, absent or present): the character is insufficiently defined. With respect to ambient salinity that differs among taxa (e.g., freshwater lampreys versus marine hagfish), the character may be modified to contrast osmoregulator against osmoconformer, those with kidneys against those without, or those with particular ion channels against those without. However, it is difficult to formulate specific characters on different modes of osmosis because comparative studies emphasized the lack of physiological traits in hagfish that are otherwise shared across gnathostomes (19). Hagfish may be truly primitive in physiology or secondarily specialized, but this cannot be meaningfully assessed in a cladistic context without sufficient character-by-character comparison with anadromous lampreys and invertebrate chordates.

CMC #99 (larval phase, absent or present): the character is vaguely defined. Hemichordates have a larval phase, whereas the presence of larvae cannot be assessed reliably for stem chondrichthyans, stem osteichthyans, and stem gnathostomes. Importantly, however, it is questionable that the larval phases present in multiple lineages can be compared to each other. Different modes of feeding, locomotion, and patterning suggest that the larval forms evolved independently in each lineage. The character may be reinterpreted in the vein of the physiological pathways capacitating metamorphosis (such as function of the thyroid hormone derivative, triiodothyroacetic acid) (112), but

the comparative analysis has not included some important living lineages (e.g., hagfish) so that the distribution of character states is parsimony-uninformative.

CMC #103 (neuromasts in sensory lines, absent or present): parsimony-uninformative after re-coding. Neuromasts have not been identified in the lateral lines of hagfish (113), but they cannot be ruled out because development of the lateral line suggests presence of the neuromast primordium in this taxon (114). The lateral line is reduced in hagfish — so the absence of neuromast in the lateral line of adult hagfish should not be confused with the absence of neuromasts in invertebrate outgroups. Coding hagfish as unknown (‘?’) renders this character uninformative. The character is excluded provisionally.

CMC #104 (relative position of atrium and ventricle of heart, well separated or close to each other): this character is invariable. The atrium and ventricle are closely associated to each other in all known taxa with a chambered heart (on which this character is contingent).

CMC #113 (mandibular branchial bar, absent or present): invariable character. There is no compelling evidence for the presence of gills in the mandibular arch in any known vertebrate, extinct or extant (115). In *Metaspriggina*, the most anterior pair of the branhial skeleton is interpreted as the mandibular arch (35). However, no anatomical correlates (trigeminal nerve, upper lip cartilages) exist to corroborate this identification, unless one assumes *a priori* that the ancestral mandibular arch derived a branchial skeleton or that the most anterior pharyngeal skeleton represents a mandibular arch — thereby creating a circular argument (115). Nor does any evidence rule out alternative interpretations (e.g., mandibular cartilages not preserved, and the putative mandibular branchial bar were derived from the hyoid arch). Regardless of positional identity, no definitive branchial fillaments can be confirmed on this most anterior pair. Even if the identification of a mandibular branchial bar in *Metaspriggina* is accepted, the character is still parsimony-uninformative because no other taxa can be scored as present.

CMC #114 (first pharyngeal bar [mandibular], undifferentiated or differentiated): the character

is insufficiently defined. It contains two internal inconsistencies. First, the most anterior pharyngeal skeleton preserved does not necessarily represent a mandibular arch derivative. The most anterior pharyngeal bar in cephalochordates cannot be treated as a mandibular arch derivative based on its anterior position alone. Second, the mandibular arch derivatives are differentiated from the rest of the pharyngeal skeleton in all known vertebrates, extinct or extant (111). But the degrees of differential morphology vary among taxa and cannot be confused with one another.

CMC #115 (eyes, absent/unpaired/ocelli or paired): redundant with CMC #12. The states are poorly defined. It is not clear whether eyes are intended to be an integrated photoreceptive organ with a lens, more narrowly with optic nerve, or broadly so as to include structures like ciliary eyes. There is no justification for grouping ocelli and paired eyes under the same character state..

CMC #116 (notochord, absent or present): parsimony-uninformative. *Haikouella* has yet to provide unambiguous evidence for the presence of a notochord except for a thin sheath (116), despite extensive preservation of muscular and other soft tissues (94, 117, 118). The state for this taxon is unknown. With only hemichordates scored for the absence of notochord in this dataset, this character would not have parsed putative chordates inside or outside chordates parsimoniously, regardless of how a notochord is inferred for these taxa.

GDS #72 (dermal skeleton in trunk, absent or present) is largely redundant with another character (#112, mineralized exoskeleton, present or absent) and is parsimony-uninformative when coded as contingent on that character.

GDS #109 (inflected myomeres in post-larval stage, absent or present): parsimony-uninformative after re-coding. Myomeres assume segmented organization and/or an axial rod (notochord). The character is thus inapplicable to hemichordates (and arguably to tunicates, as well) because neither the notochord nor axial musculature is retained post-metamorphosis.

D3. Included Characters

The characters encoding presence/absence of placode-derived structures (adenohypophysis, nasal capsules, eyes, otic capsules, lateral line) in vertebrates cannot be decoupled from the presence of ectodermal placodes. Assigning the inapplicable status to taxa lacking neurogenic ectodermal placodes (hemichordates and cephalochordates) forces all such characters to become parsimony-uninformative. Nevertheless, these characters are coded as absent for both outgroups and retained in the present dataset. This is because: (***a***) functional or anatomical homologues of these sensory/secretory structures have been proposed in vertebrate outgroups independent of the presence/absence of placodes (119–124); (***b***) the emergence of neurogenic ectodermal placodes is not necessarily coupled to the evolution of one particular sensory capsule (e.g., tunicates have placodes but none of vertebrate-like sensory capsules) (125–128), so these characters vary independently from one another; and (***c***) some putative stem vertebrates may be argued to possess or lack one or more of these sensory capsules that are decay resistant in the living analogues (1, 2, 78).

As discussed in the rationale for deleted characters, those that can be coded only for several extant taxa under our scheme tend to be parsimony-uninformative (constant, or non-polarized). One exception to this tendency is the presence of internal taste buds (#43, this analysis) — oropharyngeal chemoreceptive structures innervated by gustatory nerves (facial, glossopharyngeal, and vagus nerves) (129, 130). Coding for presence/absence of the internal taste buds (HSM #27), hagfish, invertebrate chordates, and hemichordates would be scored as absent (34). However, these taxa are not equivalent in the state of absence. Hagfish have a unique chemosensory structure called Schreiner organ, which resembles internal taste buds. The organ is innervated by the trigeminal nerve and not contingent on the purinergic signaling (130, 131). So this character may be modified to accommodate two states (internal taste buds; Schreiner organs). As Schreiner organs are identified in two hagfish species (*Eptatretus stoutii* and *Myxine glutinosa*) (131), the character state may be useful as a myxinoid synapomorphy even if it does not contribute to the resolution of cyclostome mono-/paraphyly.

Part E. List of Characters

See **Abbreviations for previous datasets** above for the identitites of datasets abbreviated here.

E1. Brain, Sensory, and Nervous systems

1. Skeletal derivatives of neural crest: 0, absent; 1, present (CMC #1: **definition modified).**

The original definition has been modified from the presence of neural crest to that of the *derivatives* of neural crest. Robust embryological evidence indicates that neural crest gives rise to the splanchocranium, a large part of the dermatocranium, and a premandibular region of the chondrocranium (132, 133). Teeth and scales are considered to derive from neural crest ectomesenchyme (134, 135), and this model is supported by cell lineage tracing in a chondrichthyan (little skate) (136). However, evidence from zebrafish suggests a mesodermal origin for their scales and dermal fin rays (137–139). Phylogenetic distribution remains unclear for cellular origins of the integumentary skeletons beyond those two terminal taxa. The taxa only known from integumentary skeletons (*Astraspis*, *Birkenia*, *Loganellia*, *Rhyncholepis*, *Turnina*, Arandaspida, Heterostraci, and Furcacaudiformes) were coded for presence.

The pharyngeal skeletal elements observed in myllokunmingiids (*Haikouichthys*, *Myllokunmingia*, and *Metaspriggina*) coded as unknown ('?') because pharyngeal cartilages occur as a mesodermal derivative in cephalochordates (93). Tunicates were originally coded as having neural crest (35), but they lack such typical neural crest derivatives (140). Although evidence points to the presence of neural crest-like cell lineages in tunicates (141, 142), the multi-potent differentiation of neural crest including skeletal derivatives is still unique to vertebrates.

2. Ectodermal placodes: 0, absent; 1, present (**new character**).

The presence of neurogenic ectodermal placodes in tunicates (122, 125–128, 143) suggests that the sensory fates of these ectodermal thickenings constitute a synapomorphy of olfactores (and

the constituent cell types and gene expressions may have their evolutionary origins deeper still). For fossil taxa and those for which embryos are not known, the presence of sensory capsules was used as an indicator.

3. Distinct prechordal head: 0, absent or weakly developed; 1, prominent (**new character**).

    One important prediction of the New Head Hypothesis is that the evolution of neural crest gave rise to the prechordal cranium (119, 144, 145). Indeed, all living vertebrates exhibit a prominent prechordal cranium to house the nasal and adenohypophyseal organs and encapsulate the forebrain. On the other hand, *Haikouichthys* and *Metaspriggina* have a notochord extending well frontally, and the nasal capsules are tucked between the eyes, whereas the mouth sits at a more posterior level (35). The character cannot be scored in hemichordates (no notochord) or tunicates (no distinct cephalization). *Euphanerops* is superficially similar to the condition in *Metaspriggina*, but the presence of midline cartilages anterior to the notochord indicates a prechordal cranium (77). Arandaspids have the eyes exposed at the anterior end, with the nasohypophyseal canals tucked between them (146). However, it is more plausible to interpret the position in light of heterostracan anatomy, where the optic tectum extends anteriorly. Heterostracans tend to have the eyes exposed in anterior positions, but internal casts indicate that they had a prominent prechordal cranium (88, 147–149).

4. Tripartite vesicles at anterior end of neural tube (prosencephalon, mesencephalon, rhombencephalon): 0, absent; 1, present (**new character**).

    Hemichordates are coded as inapplicable because they lack a fully closed, axially elongate neural tube.

5. Morphologically distinct cerebellum with corpus cerebelli: 0, absent; 1, present (CMC #7, KLV #71: **definition modified**).

    Gene expression patterns indicate cyclostomes do have the rhombic lip and medial ganglionic eminence (150). Previously, a cerebellar primordium has been considered present in lampreys

but absent in hagfish (151), but new evidence (150) reveals that (***a***) brain development in hagfish parallels that of gnathostomes in gene expression patterns more closely than that of lampreys; and (***b***) cyclostomes do exihibit similar brain regionalization patterns at the level of gene expression. Cyclostomes still lack a clearly demarcated cerebellum with corpus cerebelli, so the original definition was modified in accordance with the analysis by Khonsari and colleagues (29). Non-vertebrates lack a tripartite brain (#4, this analysis). They are coded as inapplicable (-) for this character. This character is contingent on having a vertebrate brain and cranial nerves (#4, this analysis).

6. Profundal nerve ganglion: 0, separate from trigeminal ganglion; 1, fused with trigeminal ganglion (HSM #46; **character addition**).

   In the galeaspid *Shuyu*, the roots for V1+0 and V2+3 are well separated (152) so the ganglia were likely independent as well. In osteostracans, these nerves share a narrow root under the myodome of the orbital cavity (153, 154) so the ganglia were likely fused. One conspicuous example is seen in *Belonaspis* (MNHN SVD 1005). It is difficult to assess this character in arthrodires, but the size and shape of the trigeminal canal are comparable to those in chondrichthyans (155–159), and they are tentatively coded as separated. This character is contingent on having a vertebrate brain and cranial nerves (#4, this analysis).

7. Tripartite division of facial nerve into pharyngeal, ‘pretrematic’, and ‘postrematic’ branches: 0, absent; 1, present (CMC #8, KLV #50: **definition modified**).

   The original definition in the CMC dataset concerned the presence of pretrematic branch in branchial nerves, where jawless vertebrates were coded as lacking the branch. Cyclostomes clearly have pretrematic branch in the glossopharyngeal and vagus nerves (160). The facial nerve, however, lacks the bipartite organization of pre- and post-trematic branches being separated by the hyomandibular pouch (29, 161). What appears to correspond to a pretrematic branch is a split of the postrematic branch. The lack of the pretrematic branch in the facial nerve

is also consistent with the lack of pseudobranch (gill-like folded epithelium in hyomandibular position) in cyclostomes, suggesting that the structure represents a derived state acquired in the stem of gnathostomes (115).

8. Spinal cord in cross section: 0, round; 1, flattened (CMC #9: **definition modified**).

   Shape description was modified for clarity.

9. Ventral and dorsal roots of spinal nerve: 0, separated; 1, united (CMC #10: **definition modified**).

   See the next character for a rationale of modification.

10. Ventral and dorsal roots of spinal nerve originates: 0, intersegmentally; 1, intrasegmentally (**new character**).

    This character supplements character #9 (ventral and dorsal roots united). Indeed, hagfish and gnathostomes are similar to each other for having the dorsal and ventral roots united (they are separate in lampreys). However, both roots originate at the intra-segmental level (medial to myomeres) in lampreys and gnathostomes, whereas the origin is at the inter-segmental level (medial to myosepta) in hagfish (162). To capture the full range of this variation, this addition to CMC #10 is required.

11. Mauthner fibers at rhombomere 4: 0, absent; 1, present (CMC #11: **definition modified**).

    The Mauthner fibers are identified primarily by their position at rhombomere 4. Therefore, the character is inapplicable ('-') for outgroups without a tripartite brain (#4, this analysis).

12. Pineal organ (extra-ocular photoreceptor region expressing pineal opsins): 0, absent; 1, present (CMC #3).

13. Pineal opening: 0, covered; 1, uncovered (CMC #100; **coding modified**).

    The character is inapplicable to taxa lacking a pineal organ (#12, this analysis) and/or a skull (#3, this analysis).

14. Adenohypophysis: 0, absent; 1, present (CMC #4).

15. Olfactory peduncles: 0, absent; 1, present (CMC #2).

Any taxon that scores for 0 in character #4 (tripartite vesicles at anterior end of neural tube, absent) is coded as inapplicable (-).

16. Encapsulated olfactory epithelium with external opening: 0, absent; 1, present (CMC #13: **definition modified**).

Haikouichthys and *Metaspriggina* were coded as having olfactory capsules, following the original descriptions (35, 163).

17. Position of nasohypophyseal/nasal opening: 0, terminal; 1, dorsal (CMC #16: **coding modified**).

This character describes the extent of posthypophyseal/internasohypophyseal skeleton in the snout (39, 164–166) — or, as superficially interpreted, the distance between the mouth and the nostril. If the pertinent structures in *Haikouichthys* (163) and *Metaspriggina* (35) are correlates of the nasohypophyseal system, the openings would have been terminal, not dorsal. This character is inapplicable to those without adenohypophysis or olfactory capsules (#14 and 16, this analysis). *Myxinikela* is coded for the plesiomorphic state (0, terminal). As done for other controversial taxa and characters throughout this analysis, this was verified independently by multiple authors on the original material that preserves the snout.

18. Nasohypophyseal canal: 0, blind; 1, opening into pharynx (CMC #14: **definition modified**).

Galeaspids are coded as unknown (‘?’) for this character. The existing reconstruction of their internal anatomy (152) does not indicate whether or not the nasal cavity was open to the oropharyngeal cavity. The hypophyseal canal opened into the oral cavity, but that system is separate from the nasal passage in this lineage (152), like in crown-group gnathostomes.

Palaeospondylus is coded as having a blind nasohypophyseal canal, both under the cyclostome and gnathostome models. The cyclostome model posits a basket of longitudinal bars at the anterior end of the skull as the olfactory capsule (95). If true, the olfactory cavity would be closed posteriorly by contact between this element and the neurocranium, or else the nasohypophyseal canal would have to extend below the commissure — unlike any known

cyclostomes. Thus, the canal of *Palaeospondylus* was coded as blind, consistent with a cyclostome interpretation. The character is contingent on the presence of the nasohypophyseal/nasal cavity (#14 and #16, this analysis).

19. Nasohypophyseal opening: 0, single; 1, paired (CMC #15, 101: **definition modified**).

    This character likely correlates with the organization of nasal (olfactory) capsules (#20, this analysis). On the basis of paired capsules with a single aperture in stem gnathostomes and possibly in myllokunmingiids, the primitive state (single) does not assume a particular state in character #20. However, there is no example of paired apertures with a single capsule, suggesting that the derived state (paired) in this character is contingent on the derived state of character #20. One way to treat this likely contingency is to code only those with the derived state of character #20. However, multiple stem gnathostomes (anaspids, arandaspids, thelodonts, *Jamoytius*, *Athenaegis*, etc.) are preserved with no imprints of the capsules and therefore can only be scored for the organization of the aperture. Some of them are also highly incomplete with a limited number of characters scored. To compare them with the maximum range of OTUs, this character is coded without contingency on character #20.

20. Nasal (olfactory) capsule: 0, unpaired; 1, paired (CMC #17: **definition modified**).

    Organization of the olfactory capsules can vary independent of the number of the nasohypophyseal opening. The olfactory tracts have paired organization in all known vertebrates, but the olfactory organ may be single or paired (29). Sometimes the olfactory tracts clearly split from one another as in heterostracans and crown gnathostomes.

21. Nasohypophyseal canal: 0, maintains width and height anteriorly; 1, tapers anteriorly (**new character**).

    This character only applies to myxinoids (or putative myxinoids). Among living hagfish, *Rubicundus* spp. have a tapering snout with tubular extension and with barbels originating posterior to the aperture (70, 167–169).

22. External opening of nasohypophyseal canal: 0, terminal aperture; 1, tubular extension (**new character**).

    This character only applies to myxinoids.

23. Nasohypophyseal barbels extend from: 0, rim of nasohypophyseal aperture; 1, posteriorly to nasohypophyseal aperture (**new character**).

    This character only applies to myxinoids.

24. Nasohypophyseal papillae, ventral element: 0, absent; 1, present (**new character**).

    This character only applies to myxinoids. The characters regarding the nasohypophyseal papillae originate from a comparative analysis by Mok (170).

25. Nasohypophyseal papillae, dorsal element(s): 0, midline; 1, paired (**new character**).

    This character only applies to myxinoids.

26. Eyes with pigmented retinal epithelium: 0, absent; 1, present (CMC #12: **definition modified**; HSM #19).

    Pigmented retinal epithelium is characteristic of vertebrate eyes (102, 171).

27. Eyes: 0, exposed; 1, covered by dermis; 2, covered by trunk muscles (**new character**).

    This character is compound, as the state 2 assumes coverage by the dermis as well. However, the character is included in the current form as the state 2 only applies to a nested ingroup within the crown group Myxinoidea, which is the only group of taxa with covered eyes in this dataset. The character is contingent on character #26 (presence of eye).

28. Extrinsic eye musculature: 0, absent; 1, present (CMC #18: **coding modified**).

    The character is contingent on the presence of eyes (#26, this analysis). Euconodonts are typically coded as having extraocular muscles on the basis of the fibrous soft tissues preserved in the Carboniferous euconodont *Promissum* (172). This observation constitutes one of the prominent inferences for the vertebrate affinity of conodonts, but also has attracted controversy (27). Certainly, the extraocular muscles are a collection of individual bundles of muscles,

whereas the supposed structure in *Promissum* shows no such organization. Two lines of evidence consistent with the original interpretation are the position close to the posited eye and the fibrous texture unlike typical cartilages (172). Alternatively, the tissues may be preserved in a more advanced stage of decay than assumed originally (22). In that case, it would be difficult to identify the tissue as anything more specific than remnants of cranial musculature.

Another possible interpretation is that the 'extraocular muscles' of *Promissum* represent a patch of mucocartilage-like supporting tissue. Mucocartilages in modern larval lampreys (173–177) appear morphologically similar and compatible in anatomical position with the 'muscles' of *Promissum*. All of these interpretations are compatible with the currently available evidence, and none can be readily ruled out. To highlight uncertain identity of the preserved tissues, euconodonts were scored as unknown ('?') for this character.

29. Muscles innervated by oculomotor nerve: 0, three; 1, four (**new character**) (178).

The following characters describe three major character transitions in the extraocular muscles before the chondrichthyan-osteichthyan divergence. The oculomotor nerve innervates three muscles in lampreys (dorsal ramus: superior rectus; ventral ramus: anterior oblique, internal rectus) and four in gnathostomes (dorsal ramus: superior rectus; ventral ramus: inferior oblique, inferior rectus; internal rectus [dorsal ramus in chondrichthyans; ventral ramus in osteichthyans]) (178). Osteostracans were coded as unknown ('?') for this character, because only two muscle attachments exist for the muscles of the oculomotor nerve and because neither provides direct evidence for multiple elements attached as in other vertebrate lineages (179). This character explicitly refers only to the numbers of elements so as not to assume any one of multiple possible schemes of homology among these muscles *a priori*. A reference to number of elements (and not to interpretations of homology for the individual components) allows placoderms to be scored for this character.

Depending on the identification schemes, additional characters may be formulated to

resolve ingroup relationships among gnathostomes, but this is beyond the schope of this analysis. In a brief summary, correspondence with the counterparts in crown gnathostomes is unclear for the four muscles innervated by the oculomotor nerve in placoderms. The anterior oblique (corresponding to inferior oblique in crown gnathostomes) is undisputed. The rectus inserting dorsally appears to correspond superior rectus topographically and probably received innervation from the dorsal ramus, but has been identified as internal rectus on the basis of its insertion dorsally to the anterior oblique (178). Problematically, identification of this muscle as internal rectus assumes either: (***a***) the superior rectus was lost and one additional muscle added in placoderms; or (***b***) the superior rectus independently evolved in lampreys and gnathostomes. Transposition of the extraocular muscles is necessary no matter which assignment is followed — the internal rectus passes below the anterior oblique in lampreys, whereas it typically inserts dorsal to the inferior oblique in crown gnathostomes. Therefore, it appears most parsimonious to recognize the four muscles in placoderms as homologous to those of crown gnathostomes (anterior oblique [inferior oblique], internal rectus, inferior rectus, and superior rectus; the last probably being the only muscle innervated by the dorsal ramus). This character is contingent on the presence of extraocular muscles (#28, this analysis).

30. Oblique muscle innervated by trochlear nerve: 0, posterior; 1, superior (**new character**) (178).

    This shift of the oblique muscle correlates with the shift of another oblique muscle from anterior to inferior. This character is contingent on the presence of extraocular muscles (#28, this analysis).

31. Rectus muscles innervated by abducens nerve: 0, two; 1, one (**new character**) (178).

    Lampreys have an external and posterior rectus — only the external rectus persists in crown gnathostomes (retractor bulbi are tetrapod-specific condition). On the basis of the insertion scars, placoderms likely had two muscles innervated by the abducens nerve, although the homology of the second muscle to the posterior rectus remains unresolved (178). In

osteostracans, a single myodome sits in the corresponding position, and it remains unclear whether one or two muscles attached here despite reconstructions typically depict two (179). It is scored as unknown (‘?’) in the present analysis. This character is contingent on the presence of extraocular muscles (#28, this analysis).

32. Eyes: 0, laterally placed (interorbital distance equal to width of head at that position); 1, close together near midline (interorbital distance substantially less than width of head at that position); 2, on prominent eyestalk (**new character**).

This character is contingent on the presence of eyes (#26, this analysis). The tertiary state (2, on prominent eyestalk) would be required only if *Tullimonstrum* were to be included. For the current analysis, this character is binary.

33. Cartilaginous otic capsules: 0, absent; 1, present (GDS #111; **definition modified**).

The original definition (otic capsules, absent or present) is modified to predict distribution of the character in the stem branches with respect to general decay sequences. The otic capsules are among the most resistant skeletal structures to decay (1, 2, 78, 109). Therefore, the absence of the structure may be inferred in specimens preserved with an extensive set of soft tissues that would have otherwise decayed well before the otic capsules, from the localities where cartilages and other soft tissues are readily preserved. These conditions rule out the Lesmahagow and Achanarras forms (*Achanarella*, *Ciderius*, *Cornovichthys*, *Jamoytius*) and the Miguasha forms (*Euphanerops*: most cartilages preserved in this taxon are calcified) (77, 180) from consideration — unless the structure is clearly present in fossils (e.g., *Lasanius*: MNHN specimens under study) (181). These leave several taxa from the Mazon Creek fauna and from the Chengjiang and Burgess/Marble Canyon fauna (35, 94, 117, 163, 182, 183). Among them, the identification of an otic capsule in *Haikouichthys* (182) is accepted, whereas the corresponding region is not well represented in *Myllokunmingia*. For all others, the capsule is coded as absent. This does not rule out the presence of otic placodes or capsules in those taxa

(*Haikouella*, *Pikaia*, and *Metaspriggina*), but if present, they are unlikely to have been extensively chondrified.

34. Vertical semicircular canals forming loops that are separate from roof of utriculus: 0, absent; 1, present (CMC #21; **coding modified**).

    All characters concerning the inner ear anatomy are inapplicable to taxa that lack an otic capsule (#33, this analysis).

35. Anterior and posterior semicircular canals: 0, share a canal toward utriculus after meeting in a confluence; 1, meet each other to form a single loop (**new character**).

    Although the single semicircular canal in hagfish is often described as a 'horizontal' canal, a series of stem gnathostome lineages (heterostracans, galeaspids, and osteostracans) clearly indicates that the horizontal canal is unique to jawed gnathostomes. So the anterior and posterior canals have broader distributions, and the condition in lampreys is compatible with that assignment. Given that hagfish have the two cristae in positions corresponding to the anterior and posterior canals in lampreys (184), the 'horizontal' canal of hagfish is the fusion of the anterior and posterior canals partway in their loops. Its state is best interpreted as the loss of the confluent part of the anterior and posterior loops. This character is contingent on the presence of an otic capsule (#33, this analysis).

36. Horizontal semicircular canal: 0, absent; 1, present (**new character**).

    The character is contingent of character #33.

37. Statoliths composed of calcium phosphate: 0, absent; 1, present (HSM #38).

    Cyclostomes have calcium phosphatic condensations in the inner ear (79). Calcium phosphatic condensation in the otic capsule of *Hardistiella* is accepted provisionally as a statolith, following the description by Janvier and Lund (185).

38. Endolymphatic duct: 0, is blind; 1, opens externally (CMC #22; **definition modified**).

    The original definition (presence/absence of open endolymphatic duct) confounds the

presence/absence of endolymphatic duct with its states (externally open/closed). In that original definition, presence/absence cannot be assessed for outgroups that lack an otic capsule altogether, and the character would have been parsimony-uninformative. The character definition was thus modified. The character is contingent on character #33.

39. Electroreceptive cells: 0, absent; 1, present (CMC #23).

40. Sensory lines: 0, absent; 1, present (CMC #24).

41. Sensory-lines: 0, on head only; 1, on head plus body (CMC #25).

    The character is contingent on the presence of sensory lines (#40, this analysis).

42. Sensory-line: 0, enclosed in grooves; 1, enclosed in canals (CMC #26).

    The character is contingent on the presence of sensory lines (#40, this analysis).

43. Internal taste buds, or functionally equivalent end chemosensory organs innervated by cranial nerves in head: 0, absent; 1, present; 2, lacking internal taste buds but function replaced by Schreiner organs (HSM #27; **definition modified**).

    The absence of internal taste buds in hagfish should not be confused with that in non-vertebrate chordates and hemichordates, as the function is replaced by the unique epidermal structure Schreiner organs (innervated by the non-gustatory trigeminal nerve; not requiring purinergic signaling) (130, 131). This is a specialization unique to hagfish and reported in multiple extant species. The implication is that internal taste buds and Schreiner organs are mutually exclusive, so these are treated as separate states in a single character. Alternatively, this character may be coded in two characters: (***a***) end chemosensory organs innervated by cranial nerves in head: 0, absent; 1, present; and contingent on the presence, (***b***) end chemosensory organs: 0, internal taste buds; 1, Schreiner organs. However, the character (***a***) will be constant because the character cannot be coded for outgroups that lack cranial nerves.

E2. Mouth and Branchial System

44. Preoptic head length: 0, shorter than branchial length; 1, approximately equal to branchial length; 2, longer than branchial length (**new character**).

This character potentially correlates with the presence/absence of the prechordal cranium and the posthypophsyeal/internasohypophyseal distance (#3 and #17, this analysis). However, states vary for this character primarily among those with a distinct prechordal cranium, and independently from the distance between the nostril and mouth. This character is inapplicable outside vertebrates as the preoptic head cannot be recognized in the outgroups.

45. Branchial apparatus: 0, retains arrangement of pharyngula such that first branchial opening assumes infra- to postotic position; 1, displaced anteriorly; 2, displaced posteriorly (CMC #19: **definition modified**).

The original definition in the CMC dataset concerned position of otic capsule with respect to branchial apparatus, but the relationship is reversed. This character now describes a position of the pharyngeal arch derivatives with respect to the nervous system, where the otic capsule and roots of all cranial nerves retain their relative positions to each other. The otic capsule is intended as a landmark — so this character may be assessed by using the overall configuration of structures spatially correlated with the otic capsule (with respect to the eye, the most anterior myomeres, the notochord, and others) when the otic capsule is not preserved. This strategy is sensible only for those that are unambiguously preserved with these alternative landmark structures (e.g., *Euphanerops*) (77). Therefore, this character is inapplicable to taxa in which (***a***) it is uncertain whether the otic capsule was absent or not preserved; and (***b***) other landmark structures are not comparable or not preserved (e.g., euconodonts and *Haikouella*).

For *Myxineidus*, serial swellings are interpreted as an outline of the branchial region (104). This interpretation is tentatively accepted in this analysis. The state assigned to *Myxineidus* (state 0, first branchial opening at infra- to postotic position) is distributed broadly

among vertebrates, including modern lampreys. Admittedly, this branchial interpretation remains open to question. However, whether *Myxineidus* is coded for a discrete state ('0') or as unknown ('?') does not appear to affect the topology. Coding this character unknown for *Myxineidus*, the parsimony analysis still supported strict consensus identical to Fig. S6.

46. Branchial apparatus, displaced anteriorly such that: 0, first branchial opening assume preotic position; 1, multiple branchial arches occupy preotic position (**new character**).

    This character applies only to those taxa that score for anteriorly displaced branchial apparatus in the previous character (state 1 of character #45).

47. Branchial apparatus, displaced posteriorly such that prebranchial length is: 0, less than a quarter; 1, approximately quarter; 1, greater than a third of body length (**new character**).

    This character applies only to those taxa that score for posteriorly displaced branchial apparatus in the previous character (state 2 of character #45).

48. Pharyngeal skeleton: 0, delineates pharyngeal slits with ciliary band; 1, supports well-developed branchial lamellae (CMC #34; **definition modified**).

    In hagfish, the skeleton of the branchial region consists of the extrabranchial cartilages around the excurrent ducts of the branchial pouches. This morphology is interpreted as derived ('1') for this character.

49. Main skeletal support for branchial apparatus with respect to lamellae: 0, lateral; 1, medial (CMC #112; **definition modified**).

    The original definition (branchial bars, external or internal) was modified to allow identification of states with respect to anatomical correlates. This character is inapplicable for those taxa lacking branchial lamellae (character #48, this analysis), and for *Haikouella* and *Pikaia* in which the branchial structures appear to have been exposed externally (183).

50. Pharyngeal skeleton: 0, skeletal arches fused with each other; 1, arches isolated (CMC #111; **definition modified**).

The original character distinguished between 'continuous' and 'basket-like' states, but these states are indistinguishable from one another. The taxa lacking a pharyngeal skeleton are coded as inapplicable.

51. Hyomandibular pouch: 0, blind; 1, externally open (spiracle) (HSM #24; **definition modified**).

Although the adorbital opening has been interpreted as spiracular in amphiaspidiform heterostracans and pituriaspids (115, 186, 187), this character is conservatively coded as unknown in both taxa. Beyond its position beside the orbit and otic capsule, little evidence exists for the hyomandibular identity of this opening. An alternative interpretation for this opening is a nasohypophyseal aperture (149). The amphiaspidiform condition is likely an independent derivation within heterostracans (88, 115) because this clade is nested within the Heterostraci (188–192), and because the general condition among heterostracans is blind (0) for this character. Modifying the scores for heterostracans (0/1) and pituriaspids (1) did not affect the topology presented in Fig. S6.

52. Respiratory current exits through: 0, atrial space; 1, excurrent duct (=branchial pouch); 2, parabranchial cavity (CMC #27: **definition modified**).

The original character definition in the CMC dataset requires some clarification because a 'pouch-shaped gill' is a composite of two different morphological variations — whether the skeletal support is lateral or medial, and whether the excurrent passes through a duct or a cavity. A new state (atrial space) was added to contrast vertebrates and non-vertebrate chordates. *Haikouichthys* and *Metaspriggina* exhibit a mix of features consistent with any of the possible states. In *Metaspriggina*, the branchial lamellae clearly sit lateral with respect to the skeletal arches. Normally, this should preclude pouch-like anatomy in this region, but no other correlate has been identified to conform to parabranchial cavities or an atrial space. Whether or not the skeletal elements are comparable to the vertebrate branchial skeleton may also be questioned. These taxa are coded as unknown ('?') for this character. Hemichordates are coded for the

primitive state ('0'). In this lineage, the external pharyngeal slits do not open directly into the pharyngeal cavity. Instead, internal pores open to the cavity partitioned by ciliated primordial and tongue bars and outlet through the external slits (193). Although the pores and the mesodermal bars have no exact anatomical counterparts outside this lineage, the hemichordate condition seems to parallel the atrial space in chordates better functionally and developmentally (e.g., mesodermal components lie between the external and internal outlets) than other states of this character.

53. Single confluent branchial opening: 0, absent; 1, present (CMC #28).

This character is inapplicable to those with parabranchial cavities (#52).

54. Branchial excurrent duct: 0, opens roughly at position of branchial pouch; 1, extends posteriorly (**new character**).

This character is inapplicable for those that lack branchial pouches (#52, this analysis) and for those with a single confluent branchial opening (#53). The branchial and pharyngeal pouches are not to be confused for this and other branchial characters. The branchial pouch (#52) refers to a respiratory structure, whereas the pharyngeal pouches are embryonic anlagen (115). This character seemingly overlaps with the one regarding posterior displacement of branchial apparatus (#45, 47). However, the orientation of the duct varies independently of relative position of branchial apparatus. In some taxa (such as *Athenaegis*) coding is based on the assumption that the duct must have extended posteriorly under dermal plates to have an outlet (the plates assumed to have functioned as external covers of the branchial pouches). There is no unambiguous osteological evidence for such relationship. However, the alternative state is functionally implausible for those taxa because that would imply the plates to have covered the branchial skeleton.

55. Branchial openings: 0, spaced accordingly with dimensions of branchial cavities; 1, packed closely together; 2, organized into multiple parallel rows (**new character**).

This character was evaluated with respect to dimensions of branchial pouches to ensure some independence from the previous character (#54, this analysis). For example, osteostracans have excurrent ducts extending posteriorly, but the space between the ducts remains roughly consistent with dimensions of the pouches. This is not the case in some taxa of hagfish, which score for both excurrent ducts extending posteriorly and their openings closely packed together (194). Multiple rows of external branchial openings occur in species of "*Paramyxine*" that are now considered as an ingroup nested within *Eptatretus* (70, 194). The coding for euconodonts ('1') is based on the description of *Clydagnathus windsorensis* (IGSE 13821) (23, 81, 195). The original observation has not been repeated for the putative traces of the gills in *Clydagnathus*. However, the state of soft tissue preservation in IGSE 13821 suggests that an anteroposteriorly extensive branchial series, if present, would have been identifiable unambiguously. Coding this character as unknown ('?') in euconodonts resulted in the topology identical to Fig. S6 under strict consensus of most parsimonious trees. Although interpretations did not affect the proposed relationships, the provisional coding ('1') is accepted to map character transformations broadly.

56. Number of arches (or pouches) in branchial apparatus: 0, unconstrained to five; 1, held constant at five (CMC #29: **definition modified**).

The arches and pouches of this definition refer to branchial arches and pouches (branchial bars and gills), respectively, and are not to be confused with pharyngeal arches and pouches (anlagen). The original character (branchial pouches/slits greater than 10 or less than 10) is clarified and split into two individual characters to reflect multiple levels of variation. Among crown gnathostomes, the number of pharyngeal arches almost never exceeds six (except for a few chondrichthyans), with five of them supporting branchial lamellae. The number is typically greater and varies widely among jawless vertebrates (196). *Myllokunmingia*, *Pipiscius*, and euconodonts are coded as unknown. These taxa have five serial impressions that appear to be

related to gills. In *Myllokunmingia*, this number is at odds with the preservation of the most anterior branchial pouch (182) but given the rest of the body the number is either four or five. Five pouch impressions are confirmed for *Pipiscius* on the observation of the specimen FMNH PF8346 (FMNH = Field Museum of Natural History), but it is unclear whether the number is fixed at five in the same way as in crown gnathostomes or is coincidental. The suspected gill impressions in the euconodont *Clydagnathus windsorensis* (23, 81) are elusive, but their tentative identification and comparison to the branchial pouches of *Mayomyzon* (83, 195) is accepted. These three taxa are treated as unknown for this character and coded for the following character describing variations in the number of branchial pouches.

57. Number of arches (or pouches) in branchial apparatus (unconstrained to five), maximum number: 0, greater than five and fewer than 20; 1, greater than 20 (CMC #29: **definition modified**).

    This character is contingent on the previous one (#56) and therefore inapplicable for those constrained to five arches supporting branchial lamellae. Among jawless vertebrates, the number of branchial pouches varies within and between lineages. In hagfish, the number is anywhere between four to fourteen (197). The range is similar among birkeniids (198). The range is greater among galeaspids from five to 45 plus (196). It is difficult to count the number of arches precisely in *Euphanerops*, but the number is at least 30 (77). Galeaspids and *Euphanerops* are unique among vertebrates in having substantially more than twenty branchial arches. So this number is used arbitrarily to distinguish the capacity to develop an exceptionally large number of branchial arches, and composite taxa were coded for the largest number that occurs within that lineage.

58. Number of arches (or pouches) in branchial apparatus (unconstrained to five): 0, four or five; 1, six or seven; 2, eight to ten; 3, greater than ten (**new character**).

    The previous two characters (#56, 57) described whether or not the number of branchial arches (pouches) is constrained at five and whether or not the number of the arches can exceed the

count normally observed in the development of living vertebrates, respectively. These characters do not describe most variations in the number of branchial arches among jawless vertebrate lineages in which the number is not constant or tightly controlled (196). Among these lineages, five and ten each breaks the distribution into discrete ranges. Few (some hagfish species) have only four branchial pouches, whereas several have five (197). Several lineages have more than ten branchial openings, including arandaspids, some anaspids (*Jamoytius* and *Pharyngolepis*), and probably *Cornovichthys* and *Achanarella* (196, 199, 200). Living lampreys all have seven (76). A reexamination of the holotype of *Priscomyzon* (AM 5750) revealed at least eight and as many as nine branchial arches. The count in *Hardistiella* is based on Lund and Janvier (201). Hagfish fall anywhere in this range from four to fourteen although most have six to eight (197). The character is inapplicable to taxa that score for either a constant number (five) (#56, this analysis) or an exceedingly large number (>20) of branchial pouches/arches (#57).

59. Branchial series extends: 0, substantially less than half the body length; 1, semiequal to or greater than half the body length (**new character**).

    This character correlates with the number of branchial arches (#56-58, this analysis) in that none of those constrained to five branchial arches score for the derived state. However, the character distinguishes a component of the variation independent from just meristics. The taxa coded for the derived state of this character (*Achanarella*, *Euphanerops*, and heterostracomorphs) vary in the number of branchial arches, whereas some forms with a large number of branchial arches are coded for the primitive state (e.g., anaspids, myxinoids, and osteostracans).

60. Lateral branchial openings: 0, at similar horizontal level; 1, in a posteroventrally inclined row (CMC #30; **definition modified**).

    The character is inapplicable for those with a highly depressiform profile (#82, this analysis) or those with parabranchial cavities (#52, this analysis).

61. Opercular flaps associated with branchial openings: 0, absent; 1, present (CMC #32).

Coding has been revised according to Gabbott and colleagues (32).

62. Branchial epithelium: 0, internal; 1, external (**new character**).

The suggestion of externally exposed branchial structures in *Haikouella* and *Pikaia* (183) is provisionally accepted. Still, the identity of the appendages remains open to interpretation. Coding this character as unknown ('?') in both taxa renders it constant. A parsimony analysis of this modified dataset still resulted in strict consensus identical in topology to Fig. S6.

63. External branchial openings, demarcated by: 0, single element entirely; 1, single element dorsally; 2, multiple plates; 3, a framework of multiple spines; 4, micromeres; 5, naked (mineralized exoskeleton locally absent around the openings) (**new character**).

This character is contingent on the presence of mineralized integumentary skeleton (#113, this analysis). Even though inapplicable status ('-') is assigned accordingly, these character states necessarily correlate with micro-/macromeric integumentary skeletons. Nevertheless, the character is included because some variations do exist within and among lineages (e.g., anaspids). It is defined as an unordered and compound multistate character because each state is discrete from one another and because no two states can be reasonably grouped in exclusion of others. For example, birkeniids exhibit both states 0 and 3 (198). The order of character states does not reflect any discernible trend or logic of progression.

64. Position of mouth: 0, terminal; 1, subterminal (CMC #35; **definition modified**).

Mouth orientation is correlated partly with overall body profile (#82, this analysis), but position of mouth with respect to other cranial landmarks (e.g., nasohypophyseal canal) can vary independently of the overall body profile.

65. Epidermal oral cirri: 0, absent; 1, present (**new character**).

66. Postoptically derived ectomsenchyme anterior to mandibular arch gives rise to palatal structures that: 0, meet at midline under nasal/nasohypophyseal organs; 1, meet at dorsal midline anterior to

nasohypophyseal organs and form a prominent oral roof (**new character**).

In vertebrates, three streams of neural crest cells populate the premandibular and mandibular regions (37, 39, 115, 164, 166). Among them, the postoptic stream gives rise to a posthypophyseal process (upper lip) in cyclostomes (39, 164), whereas it forms the trabecular cartilage anterior to the adenohypophysis in crown gnathostomes (165, 166, 202). Although these structures differ in topology between cyclostomes and crown gnathostomes (due to a tripartite organization of the nasohypophyseal placode in the latter), the postoptic streams still meet at the midline and extend anteriorly. The structures arising in the postoptically derived ectomesenchyme may form the palate between the nasohypophyseal canal and the oral cavity (hagfish) or shift the palate to the dorsal side into an oral hood (lampreys) (39). The character definition is formulated to describe these two phenotypes while reflecting topological differences in the structures derived from the postoptic ectomesenchyme. Invertebrate outgroups have neither neural crest ectomesenchyme nor explicit homologues of the structures used to identify the region (e.g., nasohypophyseal system, trigeminal nerve, mandibular arch, forebrain). Therefore, the character is inapplicable.

Similarly, a specific character state cannot be assigned to *Pipiscius* (‘?’) because neither preserves correlates to identify the area occupied by the postoptic ectomesenchyme. *Pipiscius* has an oral funnel similar to that of a lamprey (87, 91), but no clear statement can be made about whether it was derived from the postoptic ectomesenchyme. Coding this character in *Pipiscius* after living lampreys (‘1’), *Pipiscius* becomes a wildcard taxon that collapses the crown cyclostome and total and crown petromyzontiform nodes. Changing the scores for other related characters can resolve the polytomy (see discussion in *C4c. Analysis of characters for* Pipiscius).

If *Tullimonstrum* were to be included, the taxon cannot be scored for this character for a similar reason. The identity of putative tectal cartilages in this animal has been since questioned

(103), and no unambiguous nasohypophyseal opening has been identified (36).

However, *Haikouichthys* and *Metaspriggina* may be scored provisionally for the character on the basis of the position of the nasal capsules, eyes, and mouth (35, 163). Coding these taxa for this character potentially conflicts with the assessment for the characters about neural crest (#1) and prechordal cranium (#3). Both *Haikouichthys* and *Metaspriggina* were coded as unknown for having skeletal derivatives of neural crest (#1: ?) and lacking a prechordal cranium (#3: 0). By coding for the primitive state of this character, it implies either: (***a***) *Haikouichthys* and *Metaspriggina* potentially had neural crest ectomesenchyme migrating to occupy similar positions as in cyclostomes (#66: 0), but it did not differentiate into a prominent prechordal cranium (#3: 0) and might not even have acquired skeletal differentiation (#1: ?); or (***b***) both taxa did not have neural crest, but the cephalic ectoderm was patterned similarly (165, 202), such that the corresponding regions may be identified. These implications are considered reasonable for this analysis.

67. Velum: 0, absent; 1, present (CMC #37; **coding modified**).

    There is no cyclotome-like velum in cephalochordates. All non-vertebrate taxa are coded as inapplicable because a velum assumes the presence of a mandibular arch. Contrary to the assessment by McCoy and colleagues (36), hagfish do possess a velum. The functional necessity of having a valve/pump at the mandibular arch, coupled with some osteological correlates (115, 153, 203), led to provisional coding of galeaspids and osteostracans as having a velum or velum-like structure. This assessment is consistent with the analysis of Gabbott and colleagues (32).

68. Velar cartilages: 0, at hyomandibular position; 1, extend posteriorly (**new character**).

    This character is contingent on the presence of a velum (#67).

69. Velar cartilages, functions at terminal ontogenetic stages: 0, pump and valve; 1, valve (**new character**).

The character may partly correlate with position of velar cartilages (#68), but these characters are controlled independently to some extent, as the velum functions as both a pump and a valve in the larval stages of lampreys.

70. Velar wings: 0, absent; 1, present (**new character**).

This character only applies to petromyzontiforms. The coding follows Gill and colleagues (72) and Renaud and colleagues (73).

71. Velar tentacles, papillae or tubercles: 0, absent; 1, present (**new character**).

This character only applies to petromyzontiforms. The coding follows Gill and colleagues (72) and Renaud and colleagues (73).

E3. Circulatory System

72. Multi-chamber heart: 0, absent; 1, present (CMC #38; **coding modified**).

It is unknown ('?') whether the heart has multiple chambers in *Haikouichthys*, *Metaspriggina*, and *Myllokunmingia*.

73. Closed pericardium: 0, absent; 1, present (CMC #39).

74. Circulatory system: 0, open; 1, closed (CMC #40; **definition modified**).

The original character in the CMC dataset was defined and scored as if the circulatory system was open in vertebrates. The circulatory system of hagfish is considered closed, and the extensive subcutaneous sinus is interpreted as a specialization (204) — perhaps associated with the cutaneous exchange and transport of various substances (205). This specialization is related to the fact that hagfish are osmoconformers, but it is difficult to determine which character preceded (or capacitated) the other.

75. Massive subcutaneous sinus: 0, absent; 1, present (CMC #95; **definition modified**).

The original character (high blood pressure, absent or present) was vaguely defined. In that form, the character is redundant with the closed/open circulatory system (#74) and with the

absence/presence of a multichambered heart (#72). The character was modified to refer to the specialized subcutaneous blood sinus present in hagfish. As the sinus extends the entire body length (collected both from the head and tail), the presence of this sinus can be ruled out for stem gnathostomes with a dermal head skeleton with internal impressions.

Smooth internal surfaces of the dermal plates in heterostracans may be used as an inference for subcutaneous sinus underneath the dermal skeleton (88). This interpretation, however, is inconsistent with the role in epidermal intake and output that the sinus plays in living hagfish (206, 207). A massive subcutaneous sinus is correlated with low blood pressure (206), so it cannot serve as a hydrostatic support for the rigid dermal skeleton of a heterostracan.

76. Paired dorsal aortae: 0, absent; 1, present (CMC #41).
77. Lateral head vein: 0, drains into anterior cardinal vein or its derivative; 1, continues into (or functions as anterior extension of) anterior cardinal vein or its derivative (CMC #42; **definition modified**).

    The original character (large lateral head vein, absent or present) is vague in definition. Lampreys have (***a***) a vein collecting from the anterior and middle cerebral regions and (***b***) a vein collecting from the velar sinus, and both drain into the anterior cardinal vein (208). Hagfish have a cardinal heart (=velar sinus) collecting broadly from the head and draining into anterior cardinal vein (209). Osteostracans have a lateral head vein and an enormous marginal vein that corresponds in position to the anterior cardinal vein in other vertebrates (153, 154, 210). This makes galeaspids unique among jawless vertebrates for having an enormous lateral head vein collecting from the cerebral veins and continues into the anterior cardinal vein (152).

78. Lymphocytes: 0, absent; 1, present (CMC #43).
79. Lymphocytes antigen receptors: 0, VLR; 1, T and B (CMC #105; **coding modified**).

    The character is inapplicable to those that lack lymphocytes (#78, this analysis).

80. Subaponeurotic vascular plexus: 0, absent; 1, present (CMC #44; **coding modified**).

The character is inapplicable to non-vertebrate outgroups.

E4. Fins and Fin-folds

81. Body forms, relative length: 0, less than five times the next largest dimension (height or width); 1, greater than five but less than ten times; 2, greater than ten times (**new character**).

To be conservative with effects of taphonomy and decay, maximum dimension is interpreted at the plane of preservation (so it is more likely to underestimate relative length than to overestimate). Admittedly, body profile is a poorly defined composite character but also constitutes one of few biologically informative composite traits that can be observed in poorly preserved early vertebrates.

82. Body forms, width against height: 0, compressed or subcircular so that branchial openings are lateral; 1, depressed so that branchial openings are ventral (CMC #31; **definition modified**).

The original character (openings lateral or ventral) describes body proportions rather than branchial morphology.

83. Endoskeletal fin supports: 0, absent; 1, present (CMC #48; **definition modified**).

The original character (fin ray support, absent or present) is modified to distinguish endoskeletal and exoskeletal (dermal) components of the fin skeleton from one another. In the original dataset, fin rays (exoskeleton) and radials (endoskeleton) appear to have been confused. Contrary to the original coding, endoskeletal fin radials are present in hagfish ('1') (20, 211, 212). *Haikouella* is coded as absent ('0'). The exoskeletal component of the fin skeleton (rays) is parsimoniously uninformative when coded with contingency to mineralized exoskeleton.

84. Distinct dorsal fin: 0, absent; 1, present (CMC #45; **coding modified**).

Contrary to the assessment by McCoy and colleagues (36), hagfish do not have a separate dorsal fin. Coding strategies by Gabbott and colleagues (32) are followed for other taxa, except

for the absence of separate dorsal fin in *Haikouella* and *Metaspriggina* (‘0” in this analysis). As for *Metaspriggina*, the body outline is generally well preserved in the Burgess fauna. It is possible that this animal had a soft fin fold, but it is unlikely that *Metaspriggina* had a prominent dorsal fin as an individual unit. The dorsal fin reconstructed for *Achanarella* (199) may represent dislocated myomeres, but the original reconstruction is provisionally accepted for this analysis. Dorsal fins are typically absent in anaspids. Alternative coding and its consequence are discussed in *C4e. Analysis of characters for euphaneropids*.

85. Dorsal fins: 1, continuous or adjacent to one another; 1, set apart from each other widely (**new character**).

This character is intended to discriminate ingroup relationships of living lampreys; therefore, it is inapplicable outside the crown clade.

86. Fin(s) along dorsal midline originates: 0, above branchial series or anterior to mid-trunk; 1, above anus/anal fin or anterior; 2, posteriorly to anus/anal fin (CMC #46; **definition modified**).

The original character (dorsal fin originates above or posterior to branchial series) is contingent on the presence of distinct dorsal fin (#84, this analysis). In the new definition, the character describes functional property (fins along the dorsal midline) rather than specialization (whether dorsal fin should be interpreted as part of fin fold or a distinct unit, and whether taxa with a midline fin extending to the dorsal side should be coded or excluded). To reflect this change, a new landmark (anus/anal fin) is added to accommodate the range of variation. For cephalochordates, *Haikouella*, *Metaspriggina*, and *Pikaia*, the fin fold is distinguished by a deflection in the outline. The fin arguably extends further anteriorly in these taxa, but it is little more than a dorsal midline ridge. By this criterion, all of them except cephalochordates are coded as unknown (‘?’) or inapplicable (‘-’). In the analysis by McCoy and colleagues (36), *Astraspis* and galeaspids were originally coded for the derived state (‘1’) presumably on the assumption that the fin — if present — would have originated well posterior to the level of the

branchial series, but fin morphology is poorly understood for these taxa. Therefore, the coding is unknown ('?') for the present analysis.

87. Separate anal fin, or a distinct median ventral fin in postanal tail: 0, absent; 1, present (CMC #47; **coding modified**).

    In comparison to *Euphanerops* (77, 213–215), *Achanarella* is coded for the presence of an anal fin because of the pronounced epidermal ridge anterior to the caudal fin (199). *Cornovichthys* — a larger euphaneropid from the same locality — has a prominent ventral midline fin. The gut trace above the fin suggests that it sat anterior to the anus, hence not an anal fin (200). See the description of secondary analyses (*C4e. Analysis of characters for euphaneropids*) for the impact of this and other character scores on the anaspid topologies. As for osteostracans, it is debatable whether the ventral lobe of the terminal fin represents a modified anal fin or constitutes a part of the caudal fin. This is reminiscent of the abnormal anal fin reported for a female of *Petromyzon marinus* (216). In this analysis, they are coded conservatively as absence of the anal fin.

88. Paired skin folds (epidermal ridges) at suprapharyngeal position: 0, absent; 1, present (**new character**).

    This character discriminates thelodonts (217, 218) and is inapplicable to those with macromeric dermal plates forming a head shield (#129, this analysis). However, the margin of the shield in osteostracans and galeaspids may be argued as an epidermal ridge, and the position of the marginal vein (=anterior cardinal vein) in osteostracans indicates that the margin forms in the suprapharyngeal position corresponding to the paired flaps of thelodonts. Incidentally, this domain corresponds to the circumpharyngeal crest (219), or the suprapharyngeal head-trunk boundary. In this analysis, these taxa are coded following this assessment.

89. Constricted pectoral fins with endoskeletal elements: 0, absent; 1, present (CMC #50; **coding modified**).

McCoy and colleagues (36) coded pectoral fins as present ('1') in hagfish but the correct state is absent ('0'). Among jawless vertebrates, only osteostracans provide direct evidence of endoskeletal support for the pectoral fins (220, 221), but this condition is likely shared with pituriaspids (88, 187, 218).

90. Conspicuous preanal skin fold (epidermal ridge): 0, absent; 1, present (CMC #54; **definition modified**).

    To accommodate taxa with pelvic fins or with paired folds along ventral midline, the original definition (preanal median fold, absent or present) is modified. In *Cornovichthys*, the branchial series appears to extend close to the ventral fin, and the gut trace extending posteriorly above it (200). The ventral fin is therefore considered preanal. *Myllokunmigia* is coded as present ('1') based on observations of specimens. If *Tullimonstrum* were to be included, it should be coded as unknown ('?') as the presence of skin fold is questioned (103).

91. Preanal skin fold (epidermal ridge): 0, midline; 1, paired (CMC #49, 106; **definition modified**).

    The definition now includes pelvic fins as paired preanal epidermal ridges. The taxa without preanal skin fold (#90, this analysis) are coded as inapplicable.

92. Preanal skin fold (epidermal ridge): 0, longitudinal; 1, discrete pelvic fins (CMC #51; **definition modified**).

    As for pectoral fins, hagfish were miscoded as present in MSL dataset; this is corrected. The taxa without a preanal skin fold (#90, this analysis) are coded inapplicable. *Cornovichthys* is unique in having a single discrete preanal fin (200).

93. Caudal fin shape: 0, no distinct lobes developed; 1, ventral lobe much larger than dorsal; 2, dorsal lobe much larger than ventral; 3, dorsal and ventral lobes almost equally developed (CMC #52; **coding modified**).

    The following changes were made to the MSL dataset based on personal observations of specimens: Euconodonta, *Haikouella*, hagfish, lampreys ('0'); euphaneropids and thelodonts

(‘1’); arandaspids (‘2’). *Tullimonstrum* was not considered in this analysis, but it has no distinct lobe in this region (‘0’).

94. Chordal disposition relative to tail development: 0, isochordal; 1, hypochordal; 2, hyperchordal (CMC #53; **coding modified**)

    Based on personal observation of specimens, *Achanarella* and *Cornovichthys* are coded for the hypochordal state (‘1’). Character state would be unknown for *Tullimonstrum* (‘?’).

E5. Skeletal System

95. Skeletal elements consisting of calcium phosphate: 0, absent; 1, present (CMC #55; **definition modified**).

    The original definition (the endogenous ability to synthesize creatine phosphatase) (23) cannot be assessed adequately in living outgroups because of structural variations and diversity of creatine kinases and because of the lack of exhaustive comparison. Indeed, phosphocreatine and creatine kinase are abundant within and outside vertebrates. The modified definition distinguishes the presence/absence of mineralized skeleton consisting of calcium phosphates (which requires endogenous creatine phosphatase) (222–225). Mineralized ossicles in hemichordates are aragonitic (226) — therefore this taxon is coded as absent.

    Lampreys are originally coded for the presence of this character, presumably on the basis of otolith or otolith-like structures in the crown and stem lampreys (185, 227). However, crystallization of statoliths cannot be treated equally as mineralization of skeletal matrix (227), and the experimental calcification of lamprey cartilages (80) occurred strictly *in vitro* under high calcium concentrations. Therefore, cyclostomes are coded for the absence of this character in the present analysis (the presence of calcium phosphatic statoliths is treated in a separate character: #37, this analysis). This character describes expression of the trait. If the character is reinterpreted for the presence/absence of developmental *potentials* for calcium phosphatic

skeletal matrix, however, lampreys would be coded as present ('1') on the basis of the *in vitro* calcification (80) and hagfish as unknown ('?').

The Ca signal detected in the trunk structures of *Jamoytius* (28) is accepted as evidence for a mineralized skeleton, so this taxon is scored for the subsequent characters regarding mineralized skeletons unless stated otherwise. On the other hand, *Achanarella* and *Ciderius* are coded conservatively for the absence of mineralized skeletons ('0'). These taxa show no evidence of macroscopic mineralized skeletons, but have been closely compared with *Euphanerops* and *Jamoytius* and are substantially smaller in size than both. They have not been tested for the presence of mineralized elements as identified in *Jamoytius*. The structures in *Jamoytius* may also represent a size-related feature. So the alternative coding (not employed in the present analysis) is to score this character as unknown ('?') and to score the absence of skeletal tissues that are certainly missing (e.g., hypermineralized tissues such as dentine and enameloid).

96. Bone: 0, absent; 1, present (**new character**).

This character is contingent on the presence of calcification/ossification in endo- and/or exoskeleton (#95, this analysis). *Palaeospondylus* is coded conservatively as absent ('0'). The skeleton of *Palaeospondylus* does not consist of bone, but is histologically best compared to endochondral ossification in osteichthyans or calcified cartilages in *Euphanerops* (97). So an alternative coding (not used herein) is to score unknown ('?'). Chondrichthyans are coded the presence on the basis of acanthodians (134, 228, 229), even though bone is absent in the crown group (230).

97. Cellular bone: 0, absent; 1, present (CMC #79; **coding modified**).

The character is contingent on the presence of bone (#96, this analysis). Two alternative coding strategies exist for this and the following character: (***a***) formulate one character distinguishing acellular against cellular bone or (***b***) formulate one character each for the presence/absence of

acellular and cellular bone. The alternative (***a***) may be preferable in principle to not weight either of the characters; however, in many taxa acellular and cellular bones coexist (231, 232), and both acellular and cellular bones distribute widely among stem chondrichthyans (acanthodians) and osteichthyans (134, 233–235). So these two tissue types should be treated separately as in the alternative (***b***). To make the character for acellular bone more specific, and to distinguish types of plywood-like bone in osteichthyans, the following character for the presence/absence of acellular bone is edited to refer specifically to lamellar acellular bone seen in fossil jawless vertebrates.

98. Lamellar acellular bone (isopedine): 0, absent; 1, present (CMC #78; **definition modified**).

This character is contingent on the presence of bone (#96, this analysis). The taxa lacking them are coded as inapplicable ('-').

99. Perichondral bone: 0, absent; 1, present (CMC #74; **coding modified**).

This character is contingent on the presence of cartilage and mineralized skeleton (#95, this analysis), so it is inapplicable to the taxa that lack one or both.

100. Calcified cartilage: 0, absent; 1, present (CMC #75; **coding modified**).

As in the previous character, it is contingent on the presence of cartilage and mineralized skeleton (#95, this analysis).

101. Cellular cartilages with large mature chondrocytes (30-50 µm in diameter): 0, absent; 1, present (CMC #76; **definition modified**).

This character is contingent on the presence of cellular cartilages, so it is inapplicable to taxa lacking them (hemichordates and tunicates). Lacunae occupied by chondrocytes are unusually large in hagfish, lampreys, *Euphanerops*, and *Palaeospondylus* (>30 µm) (77, 97, 111, 174, 176, 180, 236). However, hypertrophied chondrocytes in the growth plates and articular cartilages in living gnathostomes can also reach or even exceed this size (231). The assessment of the enlarged chondrocytes as specific to those three taxa (97) may be influenced by the fact

that chondrocyte sizes are not typically reported or systematically surveyed in extant vertebrates. The currently available data still suggest that they exhibit unusual sizes of mature chondrocytes, with the exclusion of hypertrophic chondrocytes that are either transient or specific to the growing tissues (so the character definition should not refer to hypertrophy). The original definition ('huge clumped" chondrocytes, absent or present) was modified accordingly. That original character was split into two because the nested organization of chondrocytes ('clumped') varies independently.

102. Mature chondrocytes: 0, become separated and generally even spaced by extracellular matrix; 1, remain nested in a pair (CMC #76; **definition modified**).

This character is contingent on the presence of cellular cartilages, so it is inapplicable to taxa lacking cartilages (hemichordates and tunicates). Lampreys and *Euphanerops* are unusual in having chondrocytes nested in a pair within the cartilaginous matrix (77, 97, 180). This condition also occurs in soft cartilages of hagfish (111). Although such pairing organization broadly occurs in growing cartilages within and outside vertebrates (93, 111, 231), retention of such organization in fully mature chondrocytes sets lampreys and *Euphanerops* apart from others. The original definition combined this feature with large sizes of the chondrocytes (see previous character), but this condition varies independently. The character was therefore split into two, and the present character is now defined on the basis of the mature status of chondrocytes.

103. Dentine: 0, absent; 1, present (CMC #80; **coding modified**).

This character is contingent on the presence of mineralized skeleton (#95, this analysis). The identification of dentine in anaspids (33) is accepted tentatively in this analysis, although some inferences used by Keating and Donoghue (33) are insufficiently justified. For example, they refer to Smith and Hall (135) to state: "[cartilage] never occurs in dermal skeleton." However, cartilages form a component of dermal skeleton, exoskeleton, or intramembranously ossified

bones, whichever is intended by the authors (e.g., articular cartilages on dermal or intramembranously formed elements; secondary cartilages; adventitious cartilages; callus cartilages; orbitosphenoids in amphisbaenians) (231). Nor can dermal bone be readily ruled out solely on the basis of histological distinctions from the underlying layer, partly because the criteria to differentiate histological features are not clear (among the cited examples, galeaspids and placoderms show some degrees of differentiation) (237–241), and partly because little evidence justifies the assumption that two different types of dermal bone should not coexist in the same element. The association of enamel/oid and dentine would be a valid argument if the thin superficial layer is not an artifact of scanning but represents a true enamel/oid layer (230). Furthermore, it is odd that tubules are lacking in the proposed dentine in birkeniids. Accepting their observations preliminarily, however, dentine is coded as present in birkeniids. *Lasanius* is coded as unknown for the lack of information.

It is possible to consider dentine to be tubular by definition. However, this typological issue is less of a concern than whether anaspids have a component of dermal skeleton laid out by odontoblasts or not. This character may be modified to "mineralized matrix of odontoblasts" with the same distribution of character states, and would still be open to the same discussion outlined in the previous paragraph. Deleting this character did not affect tree topology.

104. Spherical/globular dentine: 0, absent; 1, present (**new character**).

This character is contingent on the presence of dentine (#103). Spherical/globular dentine occurs in anaspids and conodonts among jawless vertebrates (23, 33, 242, 243). Spherical mineralization similar to that in anaspids also occurs in galeaspids (237, 238), but there is no further support that this tissue represents true dentine. The condition in arandaspids (244) is interpreted as tubular. As in the characters describing different types of bones, two alternatives exist for this and the following character: (***a***) formulate one character distinguishing tubular versus spherical dentines or (***b***) formulate one character each for the presence/absence of

tubular and spherical dentines. The alternative (***a***) does not weight either of the characters; however, the two types coexist in conodonts and some crown gnathostomes. So these two tissue types should be treated separately as in alternative (***b***).

105. Tubular dentine: 0, absent; 1, present (CMC #80; modified by KD #84).

The original definition is modified to refer specifically to tubular dentine. Taxa lacking dentine are coded as inapplicable.

106. Tubular dentine, odontoblasts tend to: 0, retreat into pulp cavity; 1, remain in dentinous matrix (CMC #81; **definition modified**).

Given the diversity of dentinous tissues among early vertebrates, the original definition (mesodentine or orthodentine) is modified into three independent characters (#106-108, this analysis) that describe histological differences in multiple types of dentine at the level of odontoblasts. All of these three characters are contingent on the presence of tubular dentine (#105, this analysis; inapplicable to the globular/spherical dentine in anaspids). The present character distinguishes mesodentine and semidentine from other types of dentine. Different types of dentine may coexist in the same animal or within a lineage. Thus, they are coded on the basis of typical histological characteristics identified in that taxonomic unit. The character is contingent on the presence of dentine (#103, this analysis).

Conodonts are coded as having a dentine type somewhat comparable to mesodentine (23, 242, 245). *Astraspis* is considered having the grade of meta- to orthodentine (134) (coded as 0), although this assessment is at odds with interpretations of the canals invading from the pulp cavity (246, 247). Thelodonts exhibit a diversity of dentine histology (88, 134, 217). In general, however, the canaliculi show polarized but irregular branching and spacing as in *Turinia* in the grade of meta- to orthodentine. *Loganellia* is coded as having the grade of mesodentine.

107. Tubular dentine, interconnections of tubules/canaliculi for odontoblasts tend to be: 0, polarized;

1, non-polarized (CMC #81; **definition modified**).

108. Tubular dentine, interconnections of canaliculi and spacing between odontoblasts tend to be: 0, regular; 1, irregular (CMC #81; **definition modified**).

109. Enamel/oid: 0, absent; 1, present (CMC #82; **definition modified**).

As in other characters coding for skeletal tissues, the original character is split into two so that one only refers to the presence/absence and the other describes monotypic versus bitypic. The presence of enameloid is accepted preliminarily in anaspids (33) and heterostracans (248). Osteostracans are coded as present, as thyestidians unambiguously developed enameloid (249). Although thyestidians form a nested ingroup within osteostracans (250, 251), it is more plausible to consider that the potential to secrete enamel/oid is conserved within osteostracans than to assume that enamel evolved secondarily. In a similar vein, euconodonts evolved enameloid independently from the rest of vertebrates (243) but this lineage is coded as present.

Although enamel as a tissue likely evolved convergently, it also remains unclear how many times enamel, enameloid, and enamel-like tissues evolved within vertebrates. As this analysis does not heavily sample taxa at lower taxonomic levels, this character should be coded as the potential to secrete enamel or enamel-like tissues rather than the distribution of the tissue within each lineage. Alternatively, the character may be defined more finely to differentiate the known types of enamel and enamel-like tissues, but this is beyond the scope of this analysis as these characters will be parsimony-uninformative in the taxon sampling of this dataset. The present character is contingent on the presence of dentine (odontodes) (#103, this analysis). This contingency is not reciprocal (the character for dentine is not contingent on the presence of enamel/oid), however, as naked dentines occur in osteostracans and various crown gnathostomes (88, 134, 252).

110. Enamel/oid: 0, monotypic; 1, bitypic (CMC #82; **definition modified**).

The character is contingent on the presence of enamel/oid (#109, this analysis).

111. Calcification/ossification occurs in endoskeleton: 0, absent; 1, present (**new character**).

Given variations and overlaps in cell lineages and modes of mineralization to give rise to hard skeletal elements (135, 137, 139, 231, 233, 253, 254), it is difficult to formulate a character to distinguish skeletal elements by either of the criteria and score fossil OTUs on the basis of information from living models. However, endo- and exo-skeleton can be clearly distinguished on the basis of anatomy alone, with predicted functional implications. Contingent on the presence of mineralized skeletons (#95, this analysis).

112. Calcification/ossification occurs in exoskeleton: 0, absent; 1, present (GDS #76; **definition modified**).

Mineralization may occur in one or both of endo- and exo-skeletons, and the states may vary independently between internally and externally. This character is contingent on the presence of mineralized skeletons (#95, this analysis). *Lasanius* is unique in the dataset that mineralized exoskeleton only occurs in the trunk, but this is parsimoniously uninformative.

113. Mineralized integumentary skeleton in trunk, surface coverage: 0, extensive; 1, limited (with evidence for variation and potentials for reduction) (**new character**).

The character is contingent on the previous character (#112, this analysis) and is intended to discriminate the condition in taxa such as *Lasanius* and placoderms, which only have partial coverage of the trunk with mineralized scales/plates.

114. Odontodes: 0, monodontodes; 1, polyodontodes (CMC #108; **coding modified**).

In addition to contingency on the presence of odontodes (nested in the presence of dentine [#103], which is further nested in the presence of mineralized dermal skeletons [#95]), this character correlates with the sizes of dermal elements (e.g., #129, this analysis) because macromeric plates assume polyodontodes. Despite that overlap, both characters should be included in the analysis. That latter character (#129) has a broader range of application because the integumentary skeleton in some lineages (such as anaspids) does not contain typical

odontodes with a pulp cavity. These taxa are designated as inapplicable for this character (#114), and coded for micro-/macromery (#129, this analysis).

Euconodonts may be coded for this character (#114) and its contingent character (#127). Tissues that compose the conodont 'teeth' (e.g., crown tissue, spheritic mineralization) appear to have evolved in stepwise fashion within the lineage (243). Perhaps for this reason, they were coded inapplicable for this character by Gabbott and colleagues (32). Nevertheless, the presence of these mineralized tissues and the pulp cavity qualify these elements morphologically as odontodes. Euconodonts are inapplicable for characters describing micro-/macromery (e.g., #129, this analysis) because they lack mineralized integumentary skeleton. Coding this character (#114) and its contingent character (#127) uncertain for euconodonts did not affect the strict consensus topology presented in Fig. S6. All taxa that lack dentine (#103, this analysis) were scored as inapplicable.

115. Exoskeleton, organization of superficial layer: 0, spherical; 1, tubular; 2, lamellar (CMC #83; KD #78; **definition modified**).

The character is contingent on the presence of mineralized exoskeleton (#95, #112, this analysis). All taxa lacking a mineralized exoskeleton are coded as inapplicable. The original character (three-layerd exoskeleton consisting of a basal lamella, middle spongy layer, and a superficial layer, absent or present) is split into three by Keating and Donoghue (33), each describing the presence/absence of superficial, middle, and basal layers. The latter scheme better accommodates known variations in organization of the vertebrate exoskeleton. For this particular character in the present analysis (organization of superficial layer), the definition is modified from Keating and Donoghue (33) to refer to specific conditions rather than to presence/absence. This is because: (***a***) all known exoskeletons have a superficial layer (thereby making the character constant under contingency coding); and (***b***) modes of mineralization differ among lineages in a parsimony-informative manner (e.g., anaspids and galeaspids share

spherical mineralization in this layer) (33, 237).

Anaspids, thelodonts, and chondrichthyans lack the middle cancellar layer, but the latter two differ from anaspids in having the base as attachment and having a non-growing crown (88, 134, 198, 217). These two variants should not be confused in a single state of the absence of the three-layerd exoskeleton. The calcium signature in the dermis of *Jamoytius* (28) is tentatively interpreted as a degenerate form of a typical anaspid condition of having a single tissue type forming a basal lamella and a superficial layer, but the specific morphology has not been described. This taxon is therefore coded as inapplicable. Galeaspids are also interpreted as having a basal lamella and a superficial layer (variably invaded by sensory canals), regarding calcified cartilages as secondary endoskeletal lining (237, 238). The tubercles in this lineage consist of spherical mineralization that superficially resembles 'spherical dentine' of anaspids.

This character partly correlates with dentine characters (#104, #108, this analysis) but distributions do not overlap completely. This character only codes for the integumentary skeleton so that teeth and other exoskeletal elements are excluded. Furthermore, the superficial layer does not always consist of dentine. For example, galeaspids are not coded as having 'spherical dentine' (#104, this analysis), but the similarity with anaspids is accepted for this character as having a superficial layer of spherical mineralization (237, 238). The coding for placoderms is based on a survey of previous works (134, 239–241, 255).

116. Exoskeleton, vascular/cancellar layer of osteons: 0, absent; 1, present (CMC #83; modified by KD #79).

This character is contingent on the presence of mineralized exoskeletons (#112, this analysis). *Jamoytius* conclusively lacks this layer (28), regardless of whether or not this animal had a mineralized exoskeleton. Similarly, pituriaspids probably lack this layer as well. Although the histology has not been described for this lineage, their endoskeletal shields are ornamented with minute tubercles without any indication of a thick canncellar middle layer (187).

117. Exoskeleton, basal tissue: 0, basal lamella; 1, basal attachment (CMC #83; KD #80; **definition modified**).

This character is contingent on the presence of mineralized exoskeletons (#112, this analysis). As in the superficial layer, the original definition by Keating and Donoghue (33) creates an invariable character under contingency coding (all mineralized exoskeletons have basal tissue). The modified version discriminates basal lamella (most) against basal attachment (thelodonts, stem chondrichthyans). The basal attachment consists of acellular bone with abundant Sharpey's fibers.

118. Cancellar layer in exoskeleton, with honeycomb-shaped cavities: 0, absent; 1, present (CMC #84; **coding modified**).

This character only applies to those that score for having a middle cancellar layer.

119. Scale shape: 0, diamond-shaped; 1, rod-shaped (CMC #86; **coding modified**).

This character is contingent on the presence of a mineralized integumentary skeleton (#112, this analysis).

120. Oak-leaf-shaped tubercles: 0, absent; 1, present (CMC #87; **coding modified**).

Shape of tubercles likely reflects mode of growth, whereas shape of scales may be influenced by other spatial factors (such as arrangement and density). This character is contingent on the presence of a mineralized integumentary skeleton (#112, this analysis).

121. Triradiate postbranchial spines: 0, absent; 1, present (KD #116).

This character is contingent on the presence of a mineralized integumentary skeleton in the trunk (#113, this analysis).

122. Median dorsal ridge scales: 0, absent; 1, present (KD #117).

This character is contingent on the presence of a mineralized integumentary skeleton in the trunk (#113, this analysis).

123. Median dorsal ridge scales: 0, simple; 1, hooked (KD #118).

This character is contingent on the presence of median dorsal ridge scales (#126, this analysis).

124. Vascular canal systems in integumentary skeleton: 0, absent; 1, present (KD #119).

This character is contingent on the presence of a mineralized integumentary skeleton in the trunk (#113, this analysis).

125. Scales: 0, without visceral ribs; 1, with visceral ribs (KD #120).

This character is contingent on the presence of a mineralized integumentary skeleton in the trunk (#112, this analysis).

126. Oral plates; 0, absent; 1, present (CMC #88; **coding modified**).

This character is contingent on the presence of a mineralized integumentary skeleton (#113, this analysis).

127. Odontodes: 0, restricted to exoskeleton; 1, extend into in oral cavity; 2, into pharynx (CMC #89; **definition modified**).

This character is contingent on the presence of odontodes (or dentine: #103, this analysis). The original definition (denticles in pharynx, absent or present) is modified to accommodate the topological distributions of dermal denticles in various vertebrate lineages. The condition in euconodonts was evaluated on the basis of P elements (256) and general histological information (243, 245).

128. Dermal head covering in adult state: 0, absent; 1, present (CMC #90; **coding modified**).

This character is contingent on the presence of a mineralized skeleton.

129. Dermal head covering in adult state: 0, micromeric; 1, large (macromeric) dermal plates or shield (CMC #109; **coding modified**).

The inapplicable status is assigned to all taxa lacking an integumentary skeleton in the head (#128, this analysis). Micromeric and macromeric elements coexist in the dermal head covering of birkeniids, but the former category dominates in surface area. They are coded as micromeric ('0') for this character, but are also subject to the characters about specific conditions of

macromeric dermal skull elements (#129, 130, this analysis).

130. Dermal head covering, macromeric: 0, large unpaired plates covering dorsal and ventral sides; 1, covered by tesserae; 2, multiple plates (CMC #91; **definition modified**).

The original definition (large unpaired plates, absent or present) is modified to accommodate variations in the integumentary skeletons of the heads. The exact state in pituriaspids is unknown ('?'). Pituriaspids have a massive continuous endoskeletal shield, but the external morphology is poorly understood (187). It perhaps resembled the condition in osteostracans, although there is no evidence of tessarae. Although the head integumentary skeleton of birkeniids mostly consists of micromeric scales, macromeric plates form the roof (coding for '2'). This character only applies to those that scored for having macromeric dermal head covering, with an exception of birkeniids.

131. Dermal head covering, macromeric/shield: 0, head and anterior trunk continuous; 1, head and anterior trunk decoupled (**new character**).

This character is contingent on the presence of a macromeric dermal head covering (#129, this analysis).

132. Endoskeletal contribution to dermal head covering: 0, absent; 1, present (CMC #92; **definition modified**).

This character refers to calcified or ossified endoskeletal cartilages lining the dermal elements (entirely or partially), which occur in galeaspids, osteostracans, and most jawed gnathostomes. Antiarchs have some endoskeletal components to the mainly dermal skeleton at the exo-endoskeletal interface in the jaws, pectoral joints, and rhinocapsular element (221, 257), although in general the dermal plates consist of exoskeletal components (240). The character coding does not discriminate macromery versus micromery, but is contingent on having a mineralized dermal skull and endoskeleton (#111, 129, this analysis). Thelodonts are coded as inapplicable, as no bones or calcified cartilages are known from these taxa.

133. Mineralized exoskeletal circumocular elements: 0, absent; 1, present (**new character**).

This character is intended to supplement morphological information from the exoskeletal circumocular skeleton to the following two characters about the endoskeletal circumocular elements. In addition to sclerotic ossicles, dermal/exoskeletal elements delineate the orbit in many vertebrate lineages. Chondrichthyans are coded on the basis of acanthodians for this character. Contingent on the presence of mineralized exoskeleton in the head (#129, this analysis).

134. Mineralized endoskeletal circumocular elements (sclerotic elements): 0, absent; 1, present (CMC #93; **definition modified**).

The original character (sclerotic ossicles, absent or present) is modified to accommodate different types of endoskeletal circumocular skeleton. The new definition includes sclerotic elements in arandaspids (25) and optic pedicles in crown gnathostomes (159, 178, 258–261). As the character is contingent on the presence of mineralized endoskeleton (#111, this analysis), the inapplicable status has been assigned accordingly.

135. Sclerotic endoskeleton: 0, isolated circumocular elements; 1, eye capsule or stalk (CMC #94; **definition modified**).

The original definition (eye capsule, absent or present) is modified to assign isolated sclera to a primitive status and make the character contingent on the presence of mineralized endoskeletal circumocular elements.

136. Fusion of visceral (pharyngeal) skeletal arches to neurocranium: 0, absent; 1, present (CMC #56; **coding modified**).

Visceral arches are broadly interpreted in this character as skeletal elements supporting the spaces between pharyngeal slits. In hagfish, the cartilages arising in the pharyngeal arches consist of the facial cartilages (at the level of mandibular arch), visceral plate (at the level of hyoid arch), and the pharyngeal basket lateral to the velum (37, 111, 262). They are all fused to

the parachordally derived cartilage near the otic capsule and in the vinicity of the trigeminal and facial nerve roots. *Haikouichthys* and *Metaspriggina* are coded on the basis of free pharyngeal skeletal elements (35, 163). *Myxinikela* has the cartilaginous elements in the branchial region, which is connected to the neurocranial region by what appears to be collagenous structures (71, 263). In hemichordates and tunicates this character is inapplicable.

137. Denticulate/cuspidate elements of feeding apparatus, perioral: 0, absent; 1, present (CMC #58; **definition modified**).

The original definition of 'circumoral teeth' does not distinguish variants properly. For example, lampreys have multicuspidate piston tooth plates within the buccal cavity and radial circumoral teeth in the oral funnel, whereas hagfish have two pairs of multicuspidate tooth plates. Both were coded originally for the presence of circumoral teeth, but these variations should not be confused. Therefore, the criterion is whether the structures are housed within the buccal cavity or exposed periorally. Absence in jawless stem gnathostomes was evaluated on the basis of whether or not the perioral morphology would allow such structures. The ring of cuspidate plates in *Pipiscius* is interpreted as a perioral structure outside the buccal cavity, so the coding for this taxon is presence in the perioral region ('1') for this character but absence for the character #139 ('0'). Given common preservation in the local fauna, buccal cuspidate plates similar to those in *Gilpichthys* and other coeval cyclostomes — if present — would have been preserved in *Pipiscius*. For these characters (#137, #139), precise tissue types are not distinguished. Both perioral keratinous teeth in lampreys and lip scales in acanthodians qualify for the presence of this character. Likewise, conodont elements and buccal keratinous teeth qualify for the buccal counterpart of this character (#139). Tissue types and functional categories are distinguished in other characters (#126, #127, #140, #141, #142, this analysis).

138. Perioral feeding elements: 0, continuous epidermal covering; 1, discontinuous arrangement (**new character**).

This character is contingent on the presence of perioral feeding structures (character #137). Perioral plates and lip scales are present broadly among stem gnathostomes, but they are continuous epidermal structures. In contrast, the perioral teeth of lampreys are discontinuous.

139. Denticulate/cuspidate elements of feeding apparatus, housed within buccal cavity: 0, absent; 1, present (CMC #58; **definition modified**).

The original character ('circumoral' teeth, present or absent) was split into two characters, one describing perioral structures (#137) and this character describing buccal structures.

140. Longitudinally aligned tooth rows providing transverse bite: 0, absent; 1, present (CMC #64; **reverted to original definition** GCR #64; **coding modified**).

The coding as present in *Mayomyzon* is based on FMNH FR5687. *Myxineidus* is coded as present for this character given recent evidence (264). This character is contingent on the presence of buccal feeding elements, which is described by the foregoing character (#139, this analysis).

141. Perioral/buccal feeding structure consisting of keratin: 0, absent; 1, present (CMC #57; **definition modified**).

Invertebrate outgroups are coded as inapplicable for this character. *Gilpichthys* and *Pipiscius* are coded conservatively as unknown ('?'). Geochemical comparison (36) suggests the proposed feeding apparatus in these taxa had a similar composition. This assessment also applies to *Tullimonstrum*, which was the subject of that paper (36). However, the comparison did not have a reference tissue that is clearly keratin from the same locality. Alternative coding and its effects are discussed in *C4c. Analysis of characters for* Pipiscius and *C4d. Analysis of characters for* Gilpichthys.

142. Feeding apparatus forming a pulley-like system of cartilages and protractor-retractor complex derived from mandibular arch: 0, absent; 1, present (CMC #62; **definition modified**).

The original character (piston cartilage and apical plate, absent or present) only applies to

lampreys. It was therefore redefined to include the lingual and dental apparatus of hagfish, as compared by Yalden (38). The character is inapplicable to those taxa in which no clear homologue of mandibular arch can be identified. *Pipiscius* is coded as absent, as its funnel-like arrangement of the circumoral teeth precludes a structure resembling the cyclostome lingual apparatus. Euconodonts have been suggested to have a cyclostome-like lingual apparatus (86), and this is functionally consistent. However, there is no anatomical evidence to indicate such a structure in a conodont. A potential piston cartilage is preserved in *Euphanerops*, but other components of the lingual apparatus are not (77). These taxa are coded conservatively as unknown ('?').

This character is not contingent on the presence of buccal tooth plates for two reasons: (***a***) to avoid duplicating (and implicitly weighting) information about the keratinous tooth plates of living hagfish and lampreys; and (***b***) to evaluate absence of the complex outside living hagfish and lampreys. Such a pulley-like system is incompatible with the presence of oral plates (in heterostracans), perioral funnel of cuspidate plates (in *Pipiscius*), or jaws (in jawed gnathostomes). With contingency, the character would be constant within and redundant with other characters.

143. Jaws (dorsoventral bite): 0, absent; 1, present (CMC #65; **coding modified**).

This character is incompatible with the character #140 because the transversely and vertically biting apparatus cannot coexist. Therefore, those taxa scored as present for the transverse biting apparatus are scored as inapplicable for this character.

144. Keratinous tooth plate, anterior element, number of fused cusps: 0, two; 1, three (**new character**).

This character only applies to potential myxinoids. Euconodonts are inapplicable.

145. Radially organized circumoral denticulate/cuspidate plates: 0, absent; 1, present (CMC #59; **definition modified**).

This character describes externally exposed, radially organized circumoral teeth. This character cannot be scored for taxa with oral plates or jaws. *Pipiscius* is coded as unknown for this character. Although the circumoral ring of cuspidate plates in this taxon superficially resembles the circumoral teeth of lampreys, it is organized in annular fashion (not radial) and embedded deeply within the oral hood as an externally open funnel. Each cuspidate plate is elongate and its basal tissue was probably unlike those of lampreys and hagfishes — which are organized as a cone and a cap (82, 111, 265–267).

146. Circumoral keratinous teeth, number of tooth rows in lateral field: 0, three; 1, four; 2, five or greater (**new character**).

This character applies to lampreys only. For the terminology of the circumoral field, see Hubbs and Potter (74).

147. Cartilaginous trematic rings: 0, absent; 1, present (CMC #60; **coding modified**).

The extrabranchial cartilages in hagfish are considered provisionally as comparable to trematic rings in lampreys (111).

148. Axial skeleton (in addition to notochord): 0, absent; 1, present (CMC #61; **definition modified**).

The original character (arcualia, absent or present) does not capture the diversity of sclerotome-derived axial skeletons in vertebrates because the distribution of arcualia can be variably interpreted (268). Neural arches, centra, and haemal arches are the midline elements of the axial skeleton, and the centra consist of basidorsal, basiventral, interdorsal, and interventral ossification centers (162). These elements are distributed taxonomically as mosaics — lampreys have neural arches, whereas hagfish have the elements that can be interpreted as a haemal arch-like structure anatomically (53), or as vestigial vertebrae (269). *Euphanerops* appears to have all elements as calcified cartilages (77). Therefore, this character is more broadly defined and followed by three characters that describe contingent conditions of the axial skeletons. These

subsequent characters are inapplicable to those in which the axial skeleton is absent. The state for this character is also unknown for *Gilpichthys*, *Metaspriggina*, and *Myllokunmingia* despite the original coding as present ('1') by McCoy and colleagues (36). Heterostracans are coded on the basis of impressions of several internal casts (270). Despite the lack of other osteological correlates, these pits resemble impressions of an axial endoskeleton on the dorsal wall in tremataspids. Linear rounded structures in the axial lines of *Jamoytius* (28) are interesting, but there is no clear evidence to indicate they are elements of the axial skeleton. There is no evidence of the axial skeleton ('?') in galeaspids. Among the taxa excluded from the current analysis, the 'arcualia' of *Tullimonstrum* (36) have been since disputed (103). We agree that there is little evidence for arcualia in this taxon, so it would be coded as unknown ('?') (see *C4h. Exclusion of* Tullimonstrum).

149. Axial skeletons around dorsal nerve cord (=neural arches): 0, absent; 1, present (**new character**).

150. Axial skeletons around notochord (=centra): 0, absent; 1, present (**new character**).

151. Axial skeletons around dorsal aorta (=haemal arches): 0, absent; 1, present (CMC #107; **definition modified**).

In the dataset by McCoy and colleagues (36), hagfish are given the state ('2') that is not explained in the character description.

152. Parachordal cartilages: 0, absent; 1, present (**new character**).

This character describes the presence of skeletogenic paraxial mesoderm in the head. Putative cranial cartilage identified in *Metaspriggina* (35) sits in the position of a notochord sheath (111). This is provisionally interpreted as a parachordal cartilage.

153. Braincase with lateral walls: 0, absent; 1, present (CMC #67).

The character is contingent on the presence of a tripartite brain and parachordal cartilages (#4, 152, this analysis)

154. Occiput enclosing vagus and glossopharyngeal nerves: 0, absent; 1, present (CMC #69; **coding modified**).

Invertebrate outgroups are inapplicable for this character because they have no precise counterparts to cranial nerves.

155. Annular cartilage: 0, absent; 1, present (CMC #70; **coding modified**).

The structure is incompatible with the jaw (annular and jaw cartilages cannot coexist in the same animal) so jawed gnathostomes are scored as inapplicable ('-'). There is no evidence for an annular cartilage in *Pipiscius*. Although cartilages are typically represented poorly in the Mazon Creek localities (103), the annular cartilage is fairly decay-resistant (2). Nothing in the circumoral ring of *Pipiscius* indicates a supporting cartilage. Even if there was one, the cartilage would not resemble the annular cartilage of a lamprey as the ring departs markedly from the morphology of the circumoral keratinous teeth in lampreys (73). The presence of this character in *Lasanius* is on the basis of MNHN specimens under study.

156. Large oral disc: 0, absent; 1, present (CMC #71; **coding modified**).

Myxineidus is coded for the presence ('1') (264), whereas *Achanarella*, *Ciderius*, *Cornovichthys*, and *Haikouella* for the absence ('0').

157. Barbels supported by cartilages: 0, absent; 1, present (CMC #72; **definition modified**).

The character definition has been modified to allow assessment based on the outline. The cartilages supporting the barbels in living hagfish are susceptible to decay (2). The barbels in *Myxinikela* have been interpreted differently because the outline is not exactly well delineated in the holotype (FMNH PR15373) (71, 89, 90, 263, 271). The second specimen (FMNH PR8472) is currently studied, and it appears to have nasohypophyseal barbels.

158. Forked subnasal cartilage: 0, absent; 1, present (GDS #112; **coding modified**).

This character is only applicable to those in which the posthypophyseal processes meet at the midline. The presence/absence of subnasal cartilage is treated conservatively as a separate

character from the presence/absence of tectal cartilages. It could be argued that the posthypophyseal processes forming a prominent oral roof in lampreys precludes rod-like cartilages supporting sensory structures in principle, and the processes forming the floor of the nasohypophyseal canal in hagfish also precludes tectal cartilages. However, this reasoning is based solely on the two living forms of cyclostomes and is therefore circular.

159. Tectal cartilages: 0, absent; 1, present (MSL #117; **coding modified**).

This character is contingent on the presence of a posthypophyseal process meeting at the midline (cyclostome upper lip) (#66, this analysis).

E6. Miscellaneous Characters

160. Male gametes shed directly through the coelom: 0, absent; 1, present (CMC #97; **coding modified**).

Hagfish are coded as present for this character.

161. Postotic myomeres migrate anteriorly to the position of eye: 0, absent; 1, present absent (CMC #98; **coding modified**).

Forward migration of the anterior myomeres occurs in hagfish. The character assumes the absence of myomeres in the head and is therefore inapplicable to invertebrate chordates that lack one or more of the key attributes to assess character states. Instead, taxa with clear cephalization may be coded with the preservation of myomeric structures — these include *Metaspriggina* and conodonts. The position and orientation of spinal nerves in galeaspids and osteostracans indicate that the myomeres did not have anterior migration in a fashion similar to cyclostomes.

162. Inflected myomeres: 0, Z-shaped; 1, W-shaped (GDS #110).

The taxa lacking myomeres or having simple myomeres are designated as inapplicable. *Haikouella* is coded as Z-shaped myomeres (‘0’) for having a gentle inflection (94, 117). The

'myomeres' in *Metapspriggina* are interpreted as unusually thick myosepta, but this does not affect identification of the state. W-sheped myomeres are observed in some specimens of *Gilpichthys* and *Pipiscius* that are currently under study (e.g., FMNH PF8475, PF8346). The putative myomeric structures of *Tullimonstrum* (36) seem to represent some kind of segment boundaries (this does not preclude its potential place in arthropods) rather than segments themselves. If myomeres were present in this animal, the preserved structured would be myosepta. They only show minor inflection, if at all, despite the complex shape reconstructed by McCoy and colleagues (36).

163. Digestive tract: 0, follows pharynx; 1, passes (or loops) over branchial apparatus (**new character**).

In adult stages of lampreys, the digestive tract separates from the branchial passage and passes over the branchial region. Such separation or loop of the digestive tract dorsal to a branchial apparatus occurs in osteostracans (153, 154). Two conflicting interpretations have been proposed for the digestive tract of *Euphanerops*, but each reconstruction suggests either passing or looping of the tract over the branchial apparatus (77). In furcacaudiforms, the gut trace (interpreted as stomach) extends onto the dorsal side of the branchial openings, again suggesting either passing or looping of the digestive tract over the branchial apparatus (272, 273). This feature is ambiguously represented in *Turinia* (coded as unknown) (274).

164. Anus, with respect to distribution of mesoderm: 0, terminal or subterminal; 1, non-terminal (**new character**).

Terminal or subterminal anus occurs in *Pikaia* (183) and *Haikouella* (94, 117, 275). If *Tullimonstrum* were to be included in an analysis, the anus would be terminal (36, 103). The digestive tract in *Tullimonstrum* does not appear to terminate anterior to the fin flap. The tract appears to have an opening at a subterminal position in the flap, marked by a white patch (characteristic of the digestive tract in an anterior portion of the animal).

165. Globular slime glands: 0, absent; 1, present (**new character**).

Slime glands are susceptible to decay relative to other soft tissue structures (1, 2), but *Tethymyxine* shows that the mechanically strong, tightly coiled, high-performance fibres of slime (α-keratin and mucin) (276–282) are resistant to decay relative to other proteins. Given the high keratin composition, this character can be coded for the taxa from the localities that readily preserve keratin — structurally or chemically (283).

166. Number of slime glands: 0, approximately 100 or fewer; 1, substantially greater than 100 (**new character**).

The character is contingent on the presence of slime glands. The number of slime glands has served as an important taxonomic character. Although there is no clear phylogenetic trend, a clear break in distribution exists around the count of 90-110 (197).

167. Slime pores: 0, overlap region of external branchial openings; 1, do not overlap region of external branchial openings (**new character**).

The character is contingent on the presence of slime glands.

168. Gular pouch in adult male: 0, absent; 1, present (**new character**).

This character is applicable only to lampreys.

EXPLANATION OF DATA SUPPLEMENTS

These data supplements are also available in original file format at DOI: 10.6084/m9.figshare.7545002

Digital data from SRS-XRF are deposited at DOI: 10.6084/m9.figshare.7545002

Suppl. 1: Data matrix of morphological characters for maximum parsimony and Bayesian analyses.

Suppl. 2: Data marix for *Palaeospondylus*, which was not used in the main analyses. This file contains two different examples of coding for this puzzling taxon.

Suppl. 3: Data matrix for *Tullimonstrum*, which was not used in the main analyses. This file contains one example of coding for this puzzling taxon that may represent an arthropod or a mollusc, instead of a vertebrate as recently suggested.

Suppl. 4: Data matrix for the time-calibrated Bayesian clock analysis in xml format.

SUPPLEMENTARY FIGURES

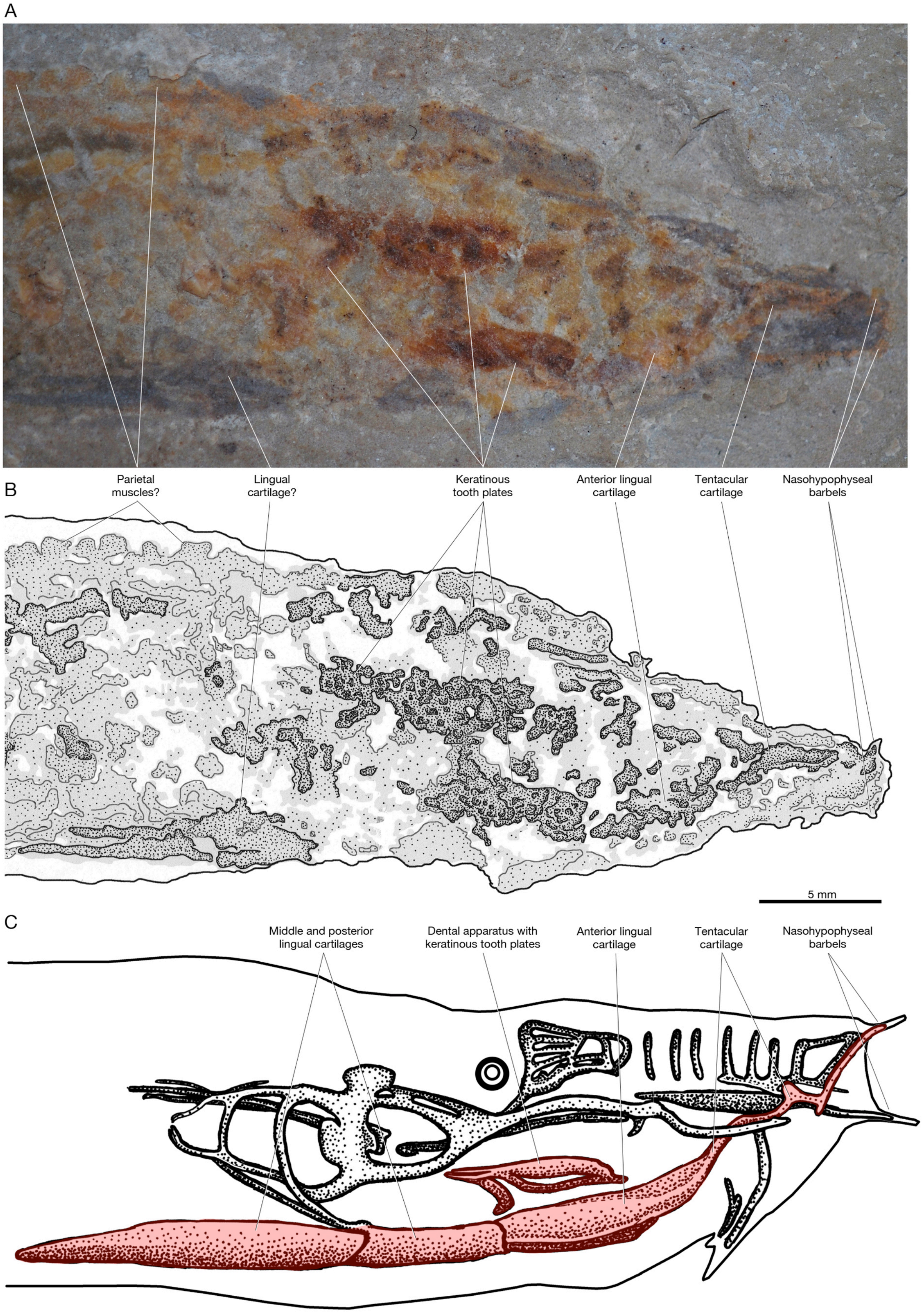

Fig. S1. The cranial anatomy of *Tethymyxine tapirostrum* (BHI 6445) in photograph (*A*) and interpretive drawing (*B*), compared to an interpretive drawing of the chondrocranium of the living hagfish *Eptatretus stoutii* (*C*), showing the elements preserved in BHI 6445 in pink shade (modified from Miyashita, 2012). The lingual cartilages and nasohypophyseal barbels are distinguished as orange-coloured structures underneath dark-colored paint. They are delineated on the basis of the surface profile and geochemical signals for various elements (Figs. S3, S4). Grey areas indicate organic preservation of tissues. Stipples indicate structural preservation of soft tissues (based on surface topography).

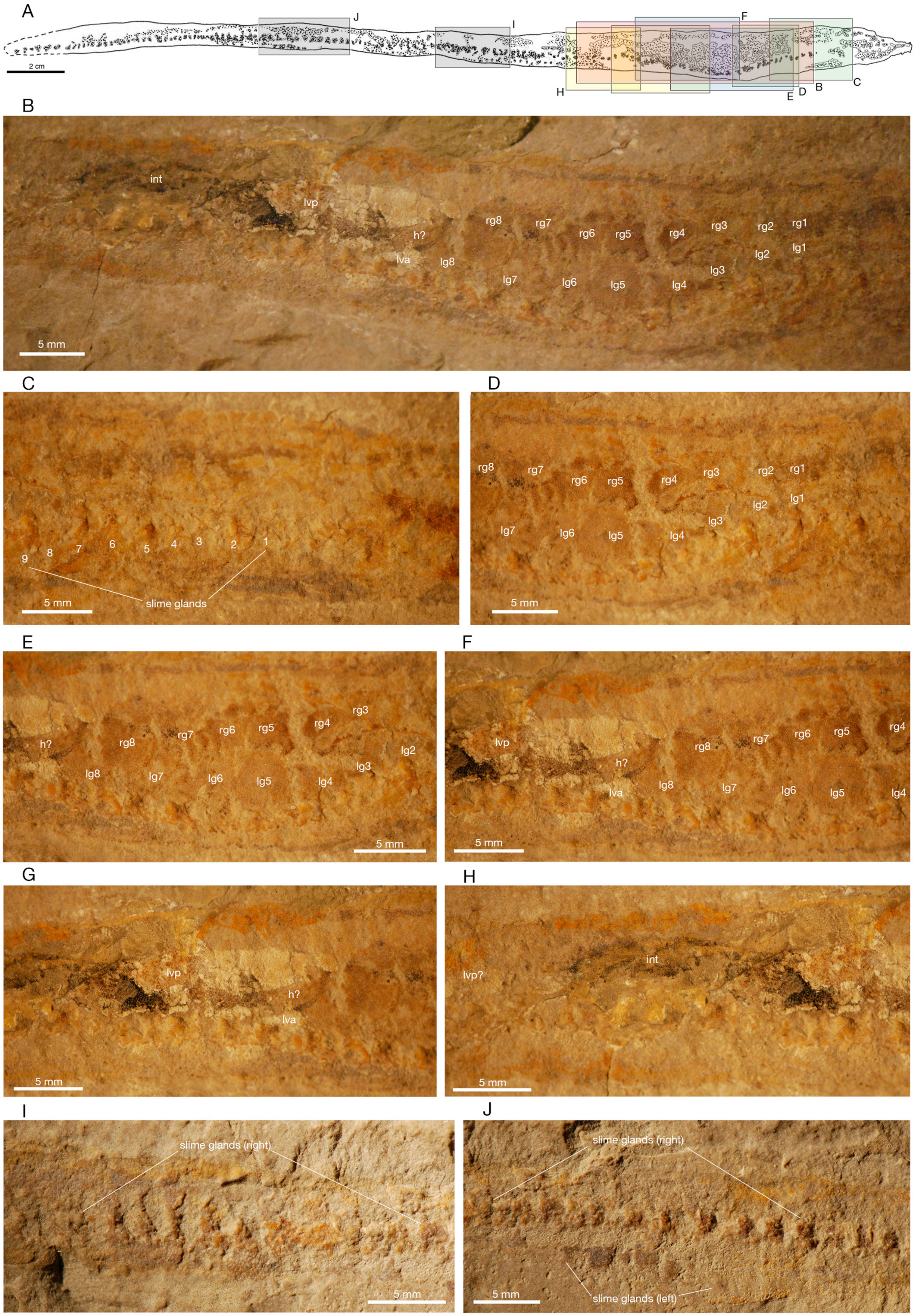
A
J
I
F
H
E
D
B
C
2 cm
B
int
lvp
h?
lva
rg8 rg7 rg6 rg5 rg4 rg3 rg2 rg1
lg8 lg7 lg6 lg5 lg4 lg3 lg2 lg1
5 mm
C
9 8 7 6 5 4 3 2 1
slime glands
5 mm
D
rg8 rg7 rg6 rg5 rg4 rg3 rg2 rg1
lg7 lg6 lg5 lg4 lg3 lg2 lg1
5 mm
E
h?
rg8 rg7 rg6 rg5 rg4 rg3
lg8 lg7 lg6 lg5 lg4 lg3 lg2
5 mm
F
lvp
h?
lva
rg8 rg7 rg6 rg5 rg4
lg8 lg7 lg6 lg5 lg4
5 mm
G
lvp
h?
lva
5 mm
H
lvp?
int
5 mm
I
slime glands (right)
5 mm
J
slime glands (right)
slime glands (left)
5 mm

Fig. S2. The trunk anatomy of *Tethymyxine tapirostrum* (BHI 6445). An interpretive drawing (*A*) to indicate regions captured in individual photographic panels (*B*-*J*): overall visceral anatomy (*B*), anterior series of slime glands (*C*), anterior (*D*) and posterior (*E*) regions of the branchial series, posterior end of the pericardial region (*F*), liver and heart (*G*), intestine (*H*), and slime glands in the mid-trunk *(I)* and close to tail (*J*). **Abbreviations:** h?, possible heart; int, intestine; lg, left branchial pouch; lva, liver, anterior lobe; lvp, liver, posterior lobe; rg, right branchial pouch.

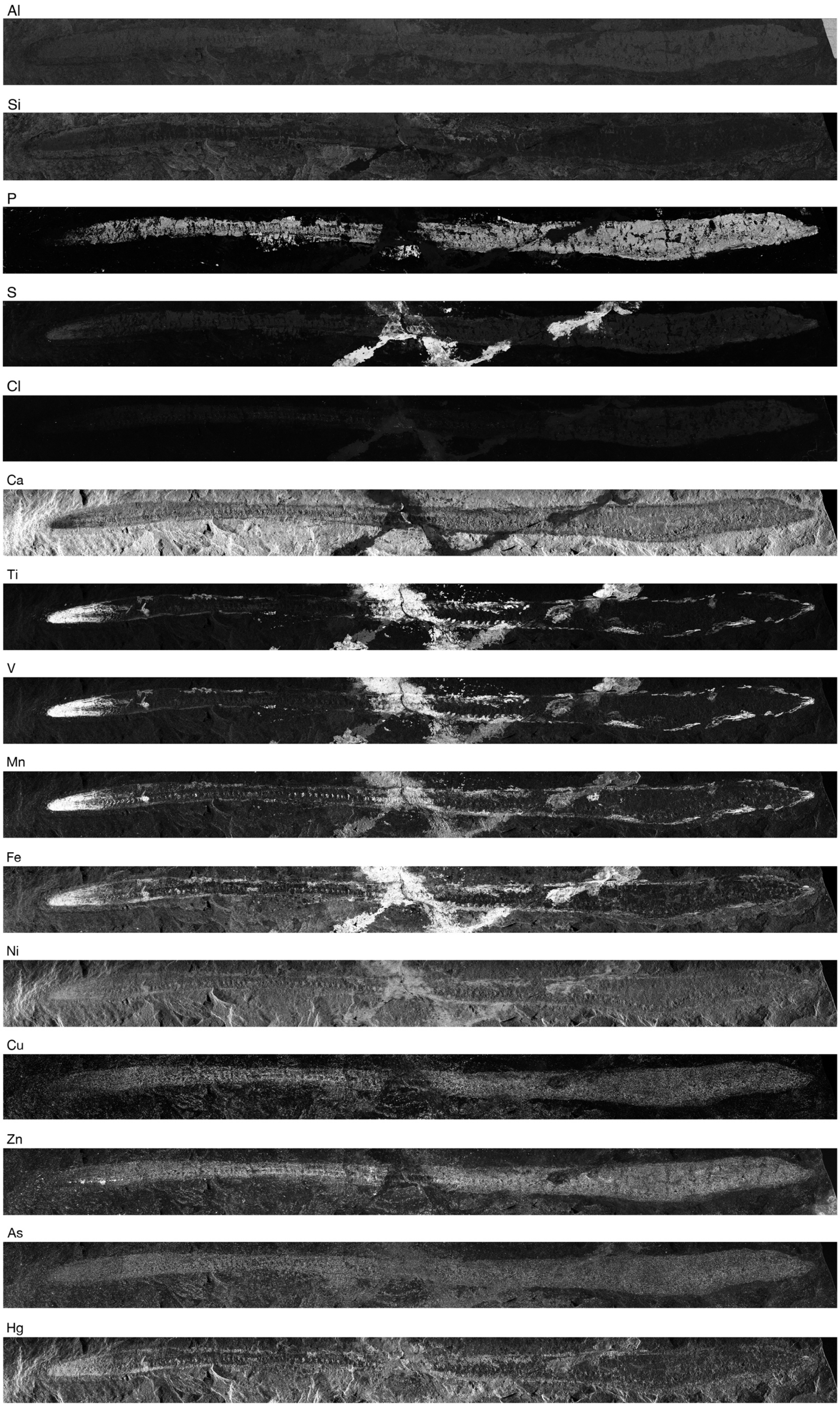
Al
Si
P
S
Cl
Ca
Ti
V
Mn
Fe
Ni
Cu
Zn
As
Hg

Fig. S3. The chemical compositions of *Tethymyxine tapirostrum* (BHI 6445) in right lateral view revealed by Synchrotron Rapid-Scanning X-Ray Fluorescence (SRS-XRF). Distributions of selected elements were reconstructed through HZ and LZ setups using a 99.9% threshold. HZ: Ca, Ti, V, Mn, Fe, Cu, Zn, As, Hg. LZ: Al, Si, P, S, Cl, Ni.

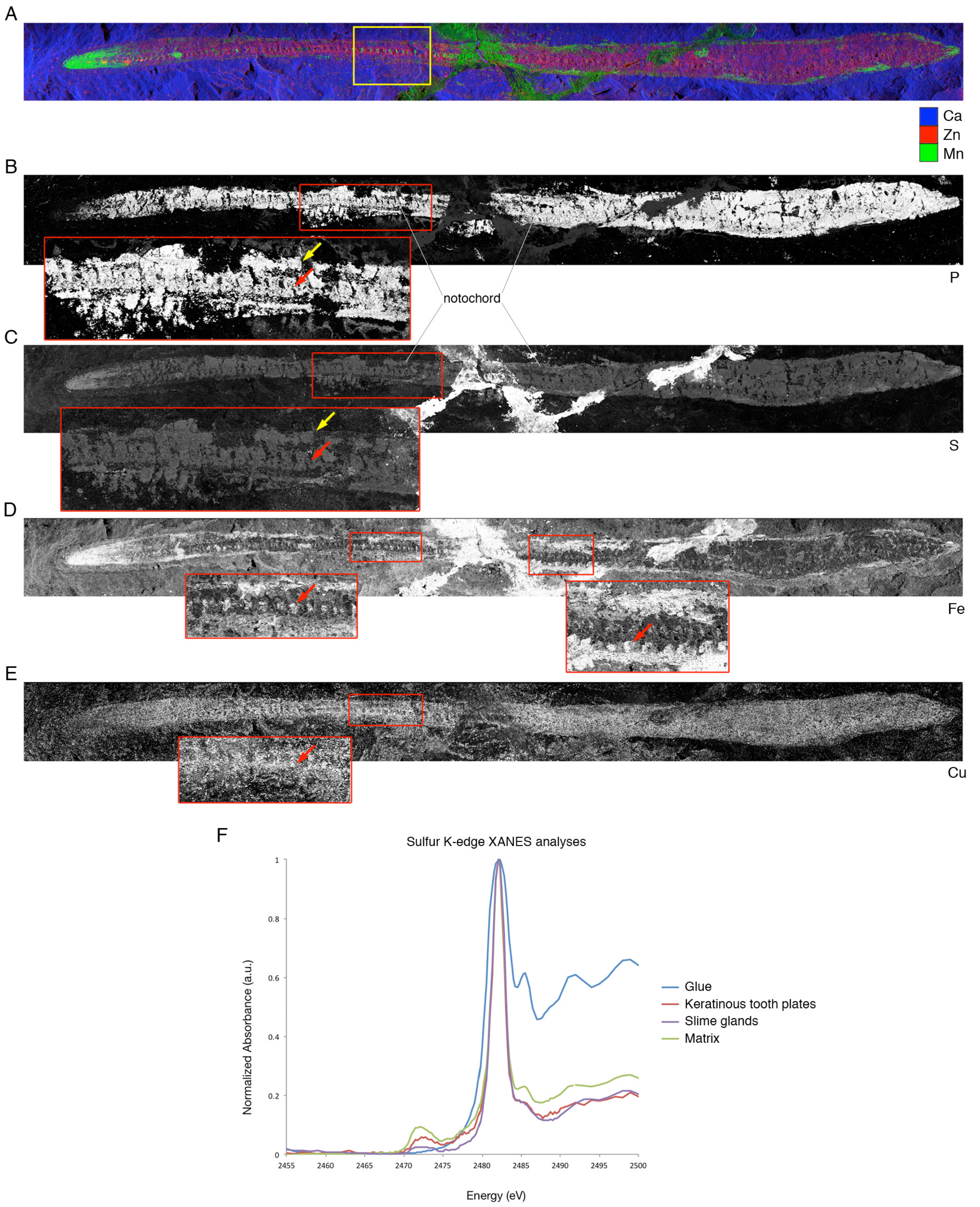

Fig. S4. Analyses of the output from SRS-XRF. The spatial distributions of trace elements highlight the slime glands and notochord of BHI 6445 in multiple elements: (*A*) false-color composite of Ca (blue), Zn (red), and Mn (green); (*B*) P; (*C*) S; (*D*) Fe; (*E*) Cu. Inset boxes show a part of the right series of slime glands. Red arrows indicate slime glands, whereas yellow arrows point to the notochord. Spectroscopy can be used to distinguish preserved tissues from the inorganically bound curatorial artifacts, which lacks organically bound sulfur. (*F*) K-edge XANES analyses of BHI6445 compared to the sedimentary matrix using sulfur (S).

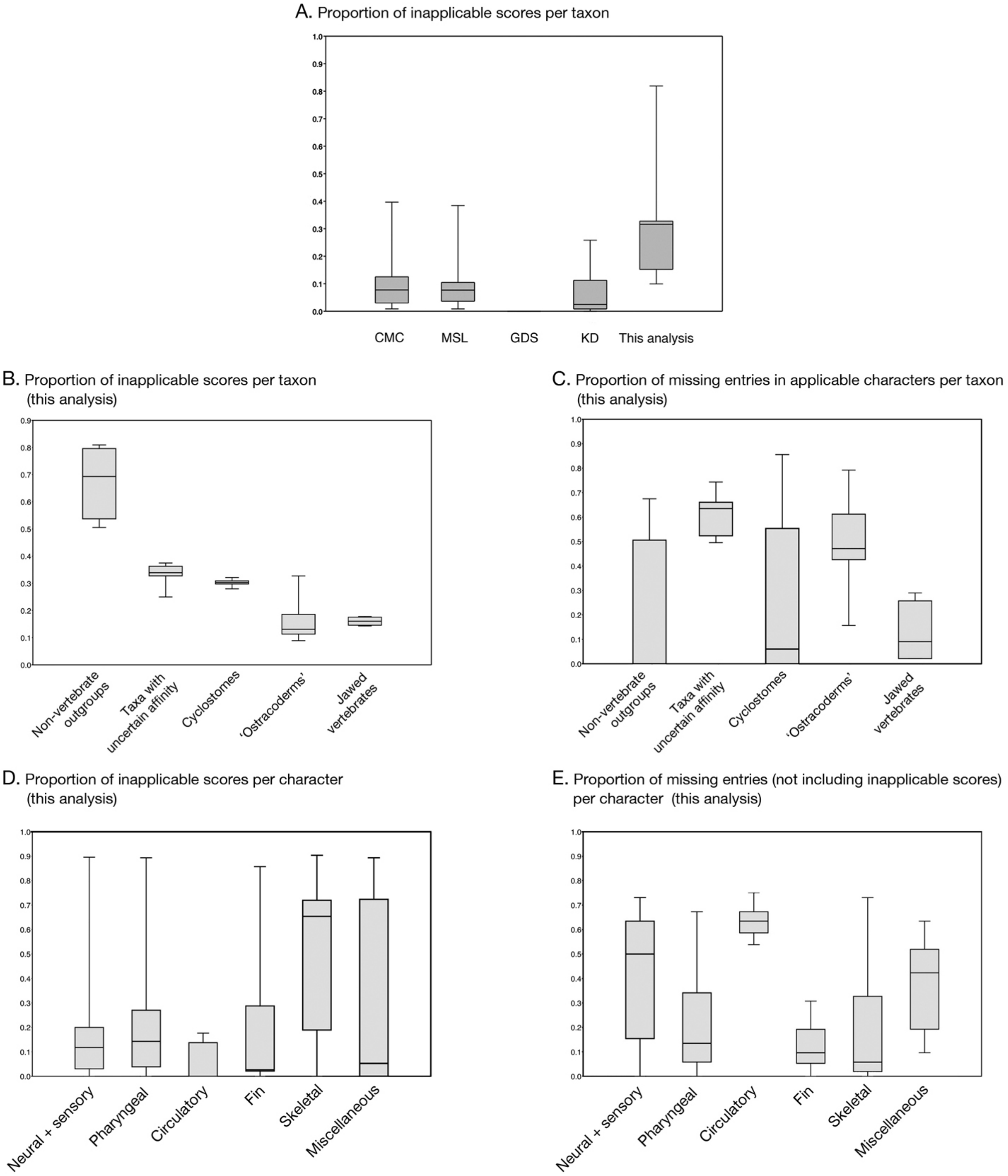

Fig. S5. Comparative analysis of the morphological dataset generated for cladistic analyses. (*A*) Our dataset contains a greater proportion of inapplicable character scores than recent datasets (CMC = Conway Morris and Caron, 2014; MSL = McCoy et al., 2016; GDS = Gabbott et al., 2016; KD = Keating and Donoghue, 2016) reflecting the contingency coding strategy. (*B*) Proportions of inapplicable scores by major taxonomic groups in our dataset. Non-vertebrate outgroups scored most highly for inapplicable states because many characters in the dataset describe vertebrate-specific morphology. Cyclostomes contain more inapplicable coding than gnathostomes, due mainly to the lack of mineralized skeletons. (*C*) In addition to inapplicable coding, information may be missing for characters. Overall, the taxa with uncertain affinity are less complete than other taxonomic categories

in our dataset. (*D*) Inapplicable codings occur in higher frequencies among skeletal characters than in other character categories in our dataset. This is because a substantial portion of the skeletal characters describes morphology of mineralized skeletons. (*E*) In contrast, skeletal characters contain lesser amount of missing entries than other character categories in our dataset, because of higher preservation potentials of mineralized skeletons. Whiskers on box plots indicate 95% confidence intervals of standard errors, and transverse lines indicate means. The GDS dataset does not distinguish inapplicable scores ('-') and missing entries ('?'); so it was not included in comparisons of inapplicable and missing character values. Percentiles were computed by interpolation.

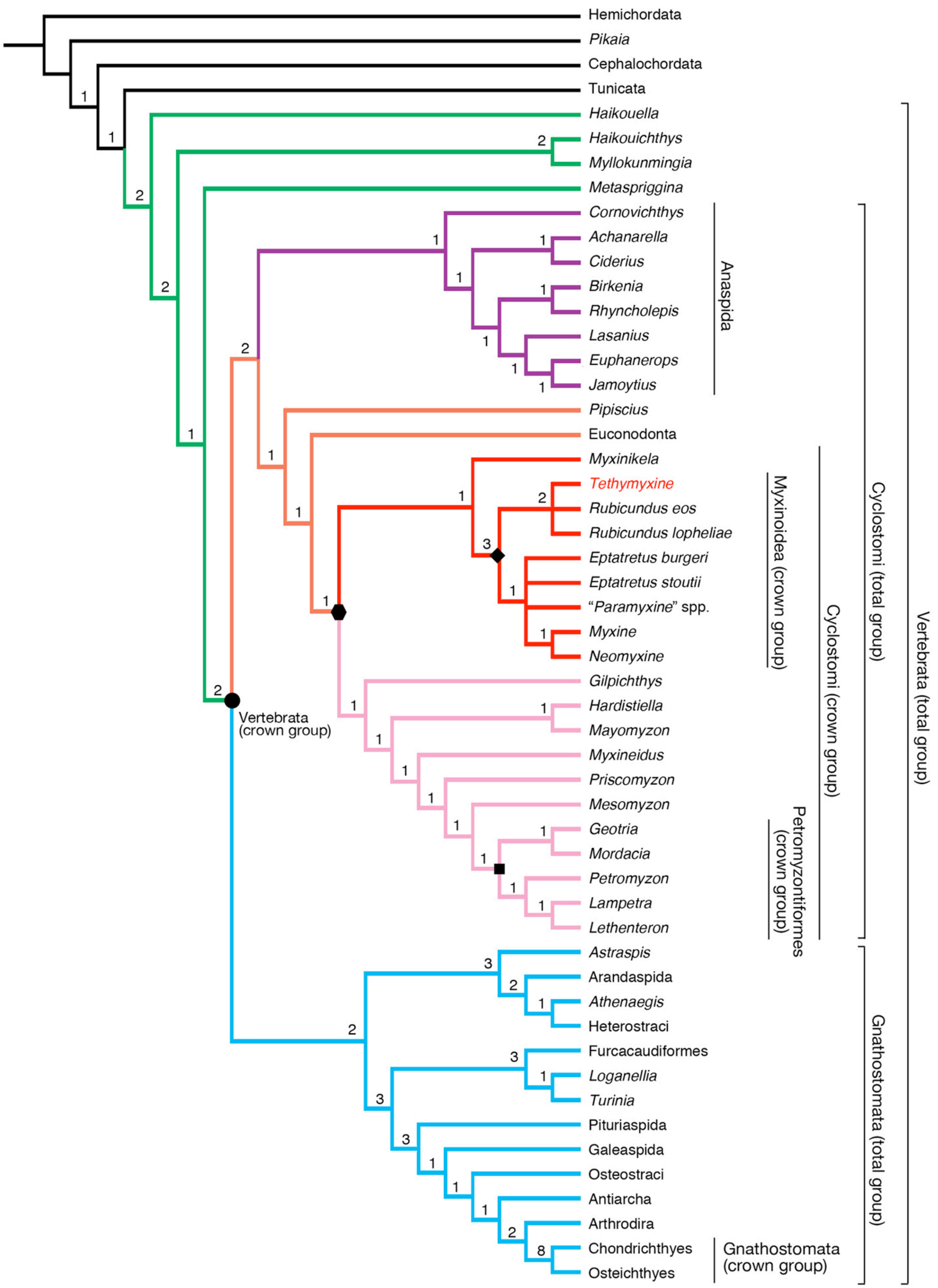

Hemichordata
Pikaia
Cephalochordata
Tunicata
Haikouella
Haikouichthys
Myllokunmingia
Metaspriggina
Cornovichthys
Achanarella
Ciderius
Birkenia
Rhyncholepis
Lasanius
Euphanerops
Jamoytius
Anaspida
Pipiscius
Euconodonta
Myxinikela
Tethymyxine
Rubicundus eos
Rubicundus lopheliae
Eptatretus burgeri
Eptatretus stoutii
"Paramyxine" spp.
Myxine
Neomyxine
Myxinoidea (crown group)
Gilpichthys
Hardistiella
Mayomyzon
Myxineidus
Priscomyzon
Mesomyzon
Geotria
Mordacia
Petromyzon
Lampetra
Lethenteron
Petromyzontiformes (crown group)
Cyclostomi (crown group)
Cyclostomi (total group)
Vertebrata (crown group)
Astraspis
Arandaspida
Athenaegis
Heterostraci
Furcacaudiformes
Loganellia
Turinia
Pituriaspida
Galeaspida
Osteostraci
Antiarcha
Arthrodira
Chondrichthyes
Osteichthyes
Gnathostomata (crown group)
Gnathostomata (total group)
Vertebrata (total group)

Fig. S6. Cyclostome monophyly under parsimony: strict consensus of maximum parsimony analysis of 52 taxa. Numerical values at each node represent Bremer support values. The branches are colour-coordinated: dark, outgroups; green, stem vertebrates; orange, stem cyclostomes; purple, anaspids (as stem cyclostome lineages); red, myxinoids; pink, petromyzontiformes; blue, gnathostomes (total group) Important nodes are labeled with symbols: filled circle = crown group Vertebrata; hexagon = crown group Cyclostomi; diamond = crown group Myxinoidea; square = crown group Petromyzontiformes. See Table S4 for the list of synapomorphies based on this strict consensus tree.

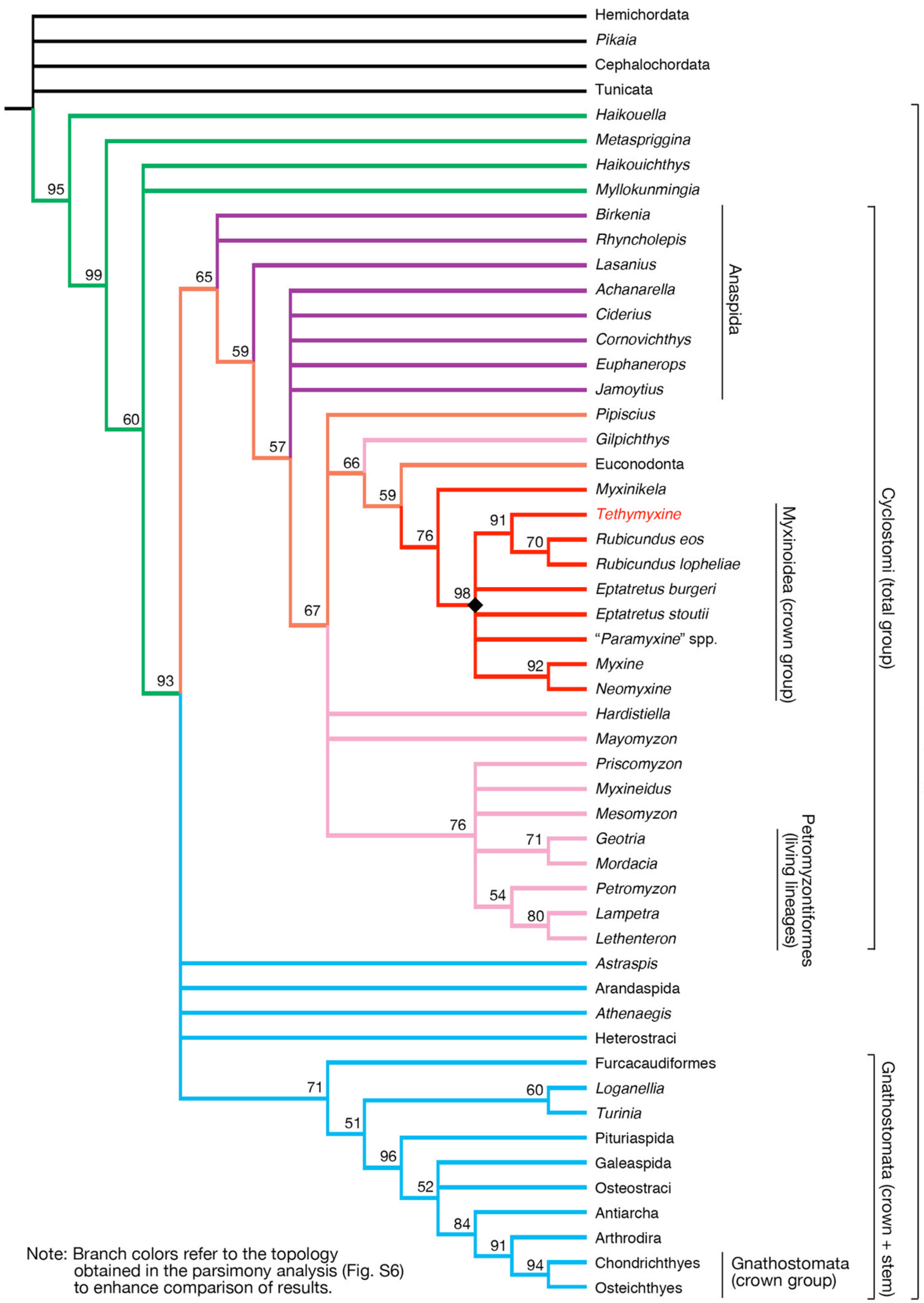

Note: Branch colors refer to the topology obtained in the parsimony analysis (Fig. S6) to enhance comparison of results.

Fig. S7. Cyclostome monophyly under Bayesian inferences. The membership of the total group of cyclostomes is identical to the maximum parsimony (Fig. S5) but precise topology differs within the clade. Numerical values indicate clade-credibility values (frequencies among sampled, supported trees). To enhance comparison of results, branch colors follow those assigned in the maximum parsimony topology in Fig. S6.

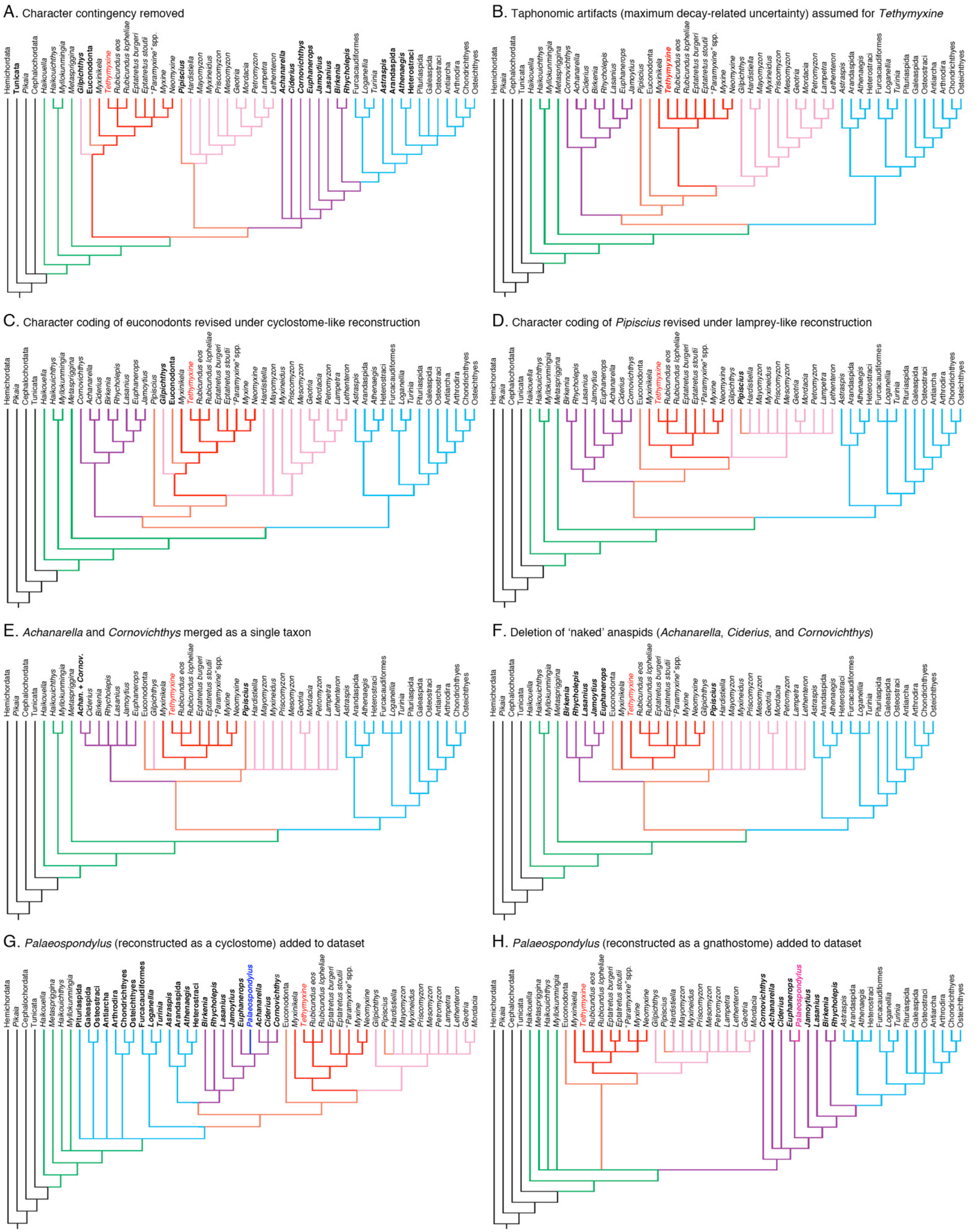
A. Character contingency removed
B. Taphonomic artifacts (maximum decay-related uncertainty) assumed for *Tethymyxine*
C. Character coding of euconodonts revised under cyclostome-like reconstruction
D. Character coding of *Pipiscius* revised under lamprey-like reconstruction
E. *Achanarella* and *Cornovichthys* merged as a single taxon
F. Deletion of 'naked' anaspids (*Achanarella*, *Ciderius*, and *Cornovichthys*)
G. *Palaeospondylus* (reconstructed as a cyclostome) added to dataset
H. *Palaeospondylus* (reconstructed as a gnathostome) added to dataset

Fig. S8. Selected results of secondary analyses using alternative character coding and taxon sampling. Taxa being modified or acting as wildcards are indicated by bold typeface. Branch colors remain unchanged from those assigned in the original topology (Fig. S6). See *SI Appendix*, Part C for detailed description of the analyses. (*A*) Cyclostomes became paraphyletic when character contingency is not considered in scoring the dataset (strict consensus of 3198 trees). In this scheme, plesiomorphic states (e.g., absence of a trait) were treated as additive among non-independent characters. The resulting topology of paraphyletic cyclostomes parallels those of previous morphology-based analyses. (*B*) *Tethymyxine* remains nested within the myxinoid crown when an advanced stage of decay is assumed (maximum ambiguity ["?"] in character scores) (strict consensus of 71 trees). (*C*) Euconodonts and *Gilpichthys* are resolved as stem myxinoids when conservative character coding is relaxed for euconodonts (strict consensus of 80 trees). In this scheme, controversial character scores of euconodonts are revised in favour of cyclostome-based interpretations of the morphology. (*D*) *Pipiscius* is nested among petromyzontiforms when conservative character coding is relaxed for this taxon (strict consensus of 90036 trees). In this scheme, character coding is revised after a lamprey-like reconstruction of the taxon. (*E*) Ingroup nodes collapse when *Achanarella* and *Cornovichthys* are assumed to represent different ontogenetic stages of a single taxon (strict consensus of 28435 trees). (*F*) Ingroup nodes collapse when enigmatic 'naked' anaspids (potential euphaneropids, not including *Euphanerops* and *Jamoytius*) are deleted from the dataset (strict consensus of 28481 trees). (*G*) *Palaeospondylus* becomes nested within anaspids, whereas gnathostome nodes are collapsed or rearranged, when the taxon is added to the dataset and coded after a myxinoid-model (strict consensus of 133878 trees). Some myxinoid characters were coded as ambiguous ('?') where comparison is tenuous. (*H*) *Palaeospondylus* is sister to *Euphanerops*, whereas anaspids now form a stem gnathostome grade, when the taxon is added to the dataset and coded after a gnathostome-model (strict consensus of 606972 trees).

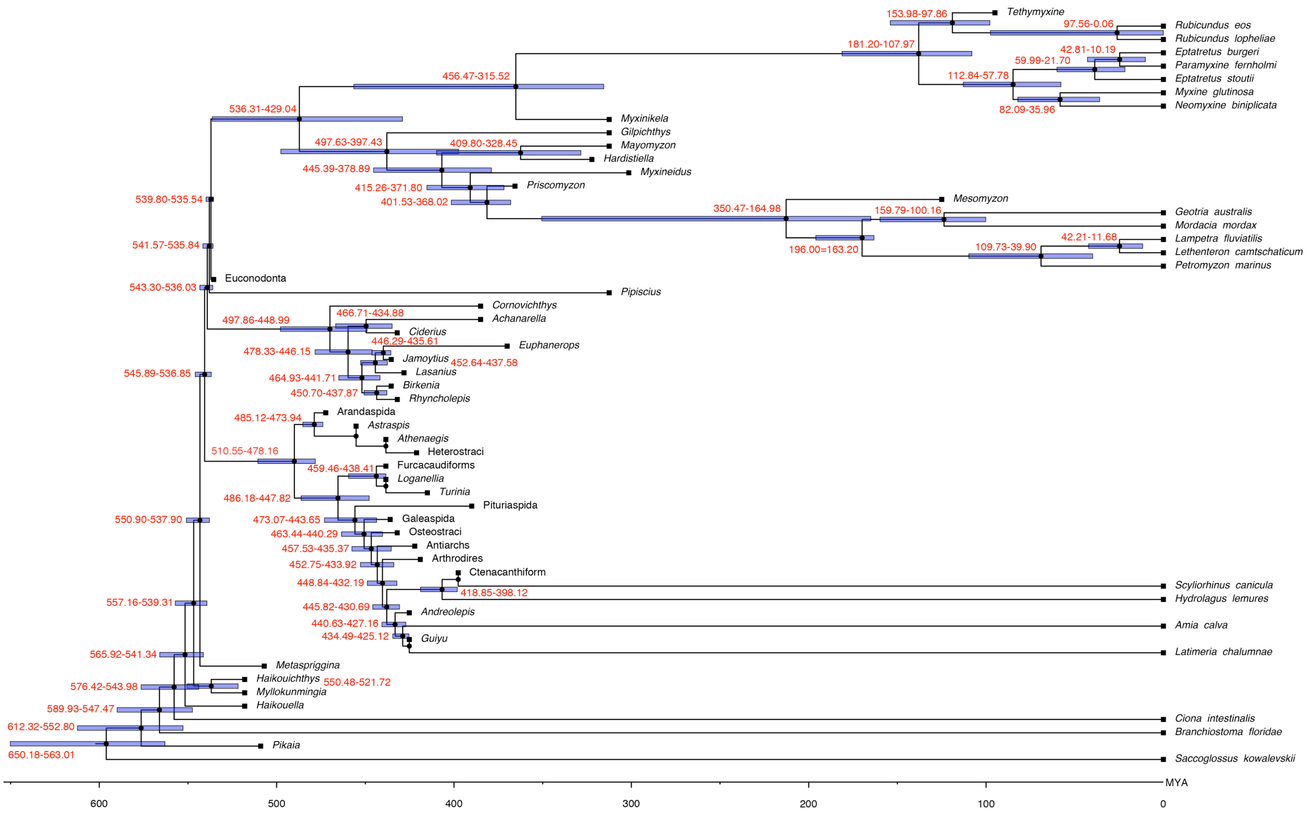

Fig. S9. Time-scaled maximum clade-credibility tree from the clock-based Bayesian analysis. The bar at each node shows 95% HPD interval for node age, indicated in MY (million years) by red font. The nodes are set at median of the frequency distribution for node-age estimates. Node ages are estimated based on the molecular clock of 16S-COI sequences calibrated with fossil occurrences in the birth-death model. Therefore, HPD is not available for nodes that: (a) collapse into a polytomy consisting of terminal, fossil taxa only in the maximum parsimony analysis (Fig. S6); or (b) split between two terminal taxa, with one represented only by fossil occurrence. For composite taxa, the branch tip represents the earliest occurrence. See *SI Appendix*, Parts B and C for methodological details. Additional data supplements are available at DOI: 10.6084/m9.figshare.7545002

SUPPLEMENTARY TABLES

Table S1. Quantification of trace elements in the holotype of *Tethymyxine tapirostrum* gen. et sp. nov (BHI 6445) compared to the surrounding matrix and glue using synchrotron analysis. Durango (fluroapatite) was used for calibration. Concentrations are in ppm or weight percent (%). Conservative 2σ error for all quantification is ~10% of the absolute value.

Element	Fluroapatite (Durango)	Glue	Matrix	Keratinous tooth plates	Slime glands	Caudal fin
Low *Z*						
Si	1990 ppm	2110 ppm	7.2 %	1.3 %	3.0 %	2.4 %
P	18.2 %	820 ppm	-	15.8 %	16 %	16.5 %
S	490 ppm	3.6 %	5710 ppm	2 %	2 %	2.3 %
Cl	7210 ppm	230 ppm	620 ppm	3310 ppm	2990 ppm	1520 ppm
High *Z*						
Ca	38.1 %	-	40.1 %	16.1 %	26.5%	19.8 %
Ti	-	-	1260 ppm	1.2 %	2220 ppm	4.2 %
Mn	-	-	20 ppm	1440 ppm	420 ppm	1280 ppm
Fe	310 ppm	-	3490 ppm	7980 ppm	5650 ppm	1.7 %
Cu	-	-	100 ppm	80 ppm	300 ppm	300 ppm
Zn	-	-	90 ppm	140 ppm	170 ppm	120 ppm
As	700 ppm	-	10 ppm	20 ppm	40 ppm	40 ppm
Ba	-	-	-	3.2 %	3250 ppm	2.9 %

Table S2. Operational taxonomic units included in our dataset for parsimony and Bayesian analyses. Cat. = taxonomic categories (used in Fig. S2): C = cyclostomes; G = gnathostomes; I = non-vertebrate outgroups; O = ostracoderms; U = uncertain affinity. Sources used to code the taxa include: S = first hand observation of specimens; L = information derived from literature or second-hand data (including photographs provided by colleagues); C = digital data derived from CT scan and other radiation or histological methods; U = information derived from unpublished specimens. *Additional taxa included only in a subset of analyses.

**Taxonomic unit**	Cat.	% Coded	Inappl.	Missing	Sources			
					S	L	C	U
Hemichordata	I	19.0%	81.0%	0.0%		X		
Cephalochordata	I	36.9%	63.1%	0.0%		X		
Tunicata	I	24.4%	75.6%	0.0%		X		
Pikaia	I	16.1%	50.6%	33.3%	X	X		
Haikouella	U	24.4%	37.5%	38.1%		X		
Myllokunmingia	U	17.3%	32.7%	50.0%		X		
Metaspriggina	U	30.4%	36.3%	33.3%	X	X		
Haikouichthys	U	32.7%	35.1%	32.1%		X		
Pipiscius	U	22.6%	33.3%	44.0%	X	X		
Gilpichthys	U	22.6%	33.9%	43.5%	X	X		X
Euconodonta	U	27.4%	25.0%	47.6%	X	X		
Myxinikela	U	28.6%	29.8%	41.7%	X	X		X
Tethymyxine	C	32.1%	28.0%	39.9%	X		X	
Eptatretus "*Paramyxine*" spp.	C	68.5%	30.4%	1.2%		X		
Eptatretus burgeri	C	68.5%	30.4%	1.2%	X	X		
Eptatretus stoutii	C	69.6%	30.4%	0.0%	X	X	X	X
Myxine spp.	C	67.9%	32.1%	0.0%	X	X		
Rubicundus eos	C	64.9%	30.4%	4.8%		X		
Rubicundus lopheliae	C	66.1%	30.4%	3.6%		X	X	X
Neomyxine biniplicata	C	64.3%	31.5%	4.2%		X		
Priscomyzon	C	31.0%	29.8%	39.3%	X	X		X
Mayomyzon	C	32.7%	30.4%	36.9%	X	X		X
Hardistiella	C	22.0%	28.6%	49.4%	X	X		
Myxineidus	U	10.1%	29.8%	60.1%	X	X		
Mesomyzon	C	34.5%	31.0%	34.5%		X		

**Taxonomic unit**	Cat.	% Coded	Inappl.	Missing	Sources S	L	C	U
Geotria australis	C	66.7%	31.0%	2.4%		X		
Mordacia spp.	C	64.9%	31.0%	4.2%		X		
Lampetra fluviatilis	C	69.0%	31.0%	0.0%	X	X		
Lethenteron camtschaticum	C	69.0%	31.0%	0.0%	X	X		
Petromyzon marinus	C	69.0%	31.0%	0.0%	X	X		X
Jamoytius	O	33.9%	15.5%	50.6%	X	X		
Euphanerops	O	42.9%	28.0%	29.2%	X	X		X
Achanarella	O	21.4%	32.7%	45.8%	X	X		
Ciderius	O	20.2%	32.7%	47.0%		X		
Cornovichthys	O	23.8%	31.5%	44.6%	X	X		
Lasanius	O	41.1%	14.9%	44.0%	X	X		X
Birkenia	O	50.0%	11.9%	38.1%	X	X		
Rhyncholepis	O	50.0%	11.9%	38.1%	X	X		
Arandaspida	O	50.0%	11.3%	38.7%	X	X		
Astraspis	O	41.7%	10.7%	47.6%	X	X		
Heterostraci	O	60.7%	11.3%	28.0%	X	X		X
Athenaegis	O	46.4%	9.5%	44.0%	X	X		
Furcacaudiforms	O	44.0%	12.5%	43.5%	X	X		X
Turinia	O	48.2%	13.7%	38.1%	X	X		
Loganellia	O	47.0%	13.7%	39.3%	X	X		
Galeaspida	O	54.2%	15.5%	30.4%	X	X		
Pituriaspida	O	18.5%	11.3%	70.2%	X	X		
Osteostraci	O	76.8%	8.9%	14.3%	X	X	X	X
Chondrichthyes	G	79.8%	16.7%	3.6%	X	X		X
Osteichthyes	G	82.1%	15.5%	2.4%	X	X		X
Antiarchs	G	58.3%	17.9%	23.8%	X	X		X
Arthrodires	G	72.0%	14.3%	13.7%	X	X		X
*Palaeospondylus**	U	25.0%	18.5%	56.5%	X	X		X

Table S3. List of synapomorphies for major nodes in a strict consensus of the most parsimonious trees generated in a maximum parsimony analysis (Fig. S6) with character states for selected taxa. Parsimony-based character optimization methods cannot distinguish missing information ('?') from inapplicable state ("-"). Thus, ACCTRAN and DELTRAN are both blind to contingency between characters. Some character changes are optimized to nodes more inclusive than those to which their parent characters are optimized. This is particularly problematic for ACCTRAN because it places a character change to the most inclusive node possible. **These spuriously optimized changes are denoted by asterisk (*).** Furthermore, additional character changes may not be optimized if an internal clade entirely consists of taxa with inapplicable scores due to contingency to another character. **These character changes with parsimoniously silent transformations are denoted by diesis (‡).**
These are known problems with the method of character optimization.

Headings: O=optimization of character changes, #=character number in this dataset, Trans.=transformation of character states.

Abbreviations: A=ACCTRAN (accelerated character transformation, reversals preferred); D=DELTRAN (delayed character transformation; convergence preferred); U=unambiguous; X=polymorphism (0+1).

Color coding: dark=unambiguous; dark grey=ACCTRAN optimization; light grey=DELTRAN optimization; green=state 0; red=state 1; orange=state 2; dark orange=state 3 and beyond; blue=missing; light cyan=inapplicable.

Vertebrata, total group

O	#	Trans.			Synapomorphies	*Tethymyxine*	*Rubicundus*	*Eptatretus*	*Myxinikela*	*Petromyzon*	*Myxineidus*	*Gilpichthys*	Euconodonta	*Pipiscius*	*Euphanerops*	*Jamoytius*	*Cornovichthys*	*Birkenia*	*Haikouella*	*Haikouichthys*	*Metaspriggina*
U	26	0	=>	1	Eye with pigmented retina, present	?	1	1	1	1	?	1	1	1	1	1	1	1	1	1	1
U	48	0	=>	1	Pharyngeal skeleton, supporting branchial lamellae	?	1	1	?	1	?	?	?	?	1	?	?	?	1	1	1
A	4	0	=>	1	Tripartite brain, present	?	1	1	?	1	?	?	?	?	?	?	?	?	?	?	?
A	14	0	=>	1	Adenohypophysis, present	?	1	1	?	1	?	?	?	?	?	?	?	?	?	?	?
A	16	0	=>	1	Olfactory capsule, present	1	1	1	1	1	?	?	?	?	1	1	?	1	?	1	1
A	20	0	=>	1	Olfactory organs, paired	?	0	0	0	0	?	?	?	?	?	?	?	?	?	1	1
A	39	0	=>	1	Electroreceptive cells, present	?	0	0	?	1	?	?	?	?	?	?	?	?	?	?	?
A	40	0	=>	1	Sensory lines, present	?	1	1	?	1	?	?	?	?	?	?	?	1	?	?	?
A	43	0	=>	1	Internal taste buds, present	?	?	2	?	1	?	?	?	?	?	?	?	?	?	?	?
A	52	0	=>	1	Branchial excurrent duct, present	1	1	1	1	1	1	1	?	1	1	1	1	1	?	?	?
A	72	0	=>	1	Multi-chambered heart, present	?	1	1	?	1	?	?	?	?	?	?	?	?	?	?	?
A	74	0	=>	1	Circulatory system, closed	?	1	1	?	1	?	?	?	?	?	?	?	?	?	?	?
A	78	0	=>	1	Lymphocytes, present	?	?	1	?	1	?	?	?	?	?	?	?	?	?	?	?
A	94	0	=>	1	Tail, hypochordal	?	1	1	?	1	?	1	1	?	1	?	1	1	?	?	?
A	148	0	=>	1	Axial skeleton (in addition to notochord), present	?	1	1	?	1	?	?	?	?	1	?	?	?	?	1	?
D	81	1	=>	0	Body length, shorter than five times width or height	2	2	2	1	2	2	1	2	1	1	1	1	0	0	1	0

Vertebrata, crown group + Myllokunmingiida

O	#	Trans.			Synapomorphies	*Tethymyxine*	*Rubicundus*	*Eptatretus*	*Myxinikela*	*Petromyzon*	*Myxineidus*	*Gilpichthys*	Euconodonta	*Pipiscius*	*Euphanerops*	*Jamoytius*	*Cornovichthys*	*Birkenia*	*Haikouella*	*Haikouichthys*	*Metaspriggina*
U	83	0	=>	1	Endoskeletal fin supports, present	?	1	1	?	1	?	?	1	1	1	?	1	1	0	1	?
U	162	0	=>	1	Myomeres, inflected into W-shape	?	1	1	1	1	?	1	1	1	1	1	?	?	0	1	1
A	1	0	=>	1	Neural crest (skeletal derivatives), present	1	1	1	1	1	?	?	?	1	1	1	?	1	0	?	?
A	33	0	=>	1	Otic capsule (cartilaginous), present	?	1	1	1	1	?	1	?	1	?	?	1	?	0	1	0
A	60	0	=>	1	Branchial openings in a posteroventrally inclined row	?	0	0	?	1	?	0	?	1	1	?	1	1	0	?	?
D	16	0	=>	1	Olfactory capsule, present	1	1	1	1	1	?	?	?	?	1	1	?	1	?	1	1

O	#	Trans.			Synapomorphies	*Tethymyxine*	*Rubicundus*	*Eptatretus*	*Myxinikela*	*Petromyzon*	*Myxineidus*	*Gilpichthys*	Euconodonta	*Pipiscius*	*Euphanerops*	*Jamoytius*	*Cornovichthys*	*Birkenia*	*Haikouella*	*Haikouichthys*	*Metaspriggina*
D	20	0	=>	1	Olfactory organs, paired	?	0	0	0	0	?	?	?	?	?	?	?	?	?	1	1
D	52	0	=>	1	Branchial excurrent duct, present	1	1	1	1	1	1	1	?	1	1	1	1	1	?	?	?
D	148	0	=>	1	Axial skeleton (in addition to notochord), present	?	1	1	?	1	?	?	?	?	1	?	?	?	?	1	?

Vertebrata, crown group + *Metaspriggina*

O	#	Trans.			Synapomorphies	*Tethymyxine*	*Rubicundus*	*Eptatretus*	*Myxinikela*	*Petromyzon*	*Myxineidus*	*Gilpichthys*	Euconodonta	*Pipiscius*	*Euphanerops*	*Jamoytius*	*Cornovichthys*	*Birkenia*	*Haikouella*	*Haikouichthys*	*Metaspriggina*
U	90	1	=>	0	Preanal skin fold (epidermal ridge), absent	1	1	1	0	0	?	0	0	0	1	1	1	1	1	1	0
U	152	0	=>	1	Parachordal cartilages, present	1	1	1	1	1	?	?	?	?	1	?	?	?	0	0	1

Vertebrata, crown group

O	#	Trans.			Synapomorphies	*Tethymyxine*	*Rubicundus*	*Eptatretus*	*Myxinikela*	*Petromyzon*	*Myxineidus*	*Gilpichthys*	Euconodonta	*Pipiscius*	*Euphanerops*	*Jamoytius*	*Cornovichthys*	*Birkenia*	*Haikouella*	*Haikouichthys*	*Metaspriggina*
U	3	0	=>	1	Distinct prechordal head, present	1	1	1	1	1	1	1	0	?	1	1	1	1	?	0	0
U	49	1	=>	0	Branchial skeleton, lateral to lamellae	?	0	0	0	0	?	?	?	?	0	0	0	?	-	1	1
U	64	1	=>	0	Mouth, terminal	1	1	1	?	0	?	0	?	0	1	1	?	0	1	1	1
U	136	0	=>	1	Visceral skeletal arches, fused to neurocranium	1	1	1	1	1	?	?	?	?	?	?	?	?	?	0	0
U	163	0	=>	1	Digestive tract, separate from branchial duct	0	0	0	?	1	?	?	?	?	1	?	?	?	0	?	0
A	50	1	=>	0	Pharyngeal skeleton, arches fused to each other	?	0	0	0	0	?	?	?	?	0	0	0	?	?	1	1
A	58	1	=>	0	Number of branchial pouches, four or five	2	0	3	0	1	?	1	0	0	-	3	3	2	1	1	1
A	153	0	=>	1	Braincase with lateral walls, present	?	0	0	0	1	?	?	?	?	1	?	?	?	-	-	0
D	1	0	=>	1	Skeletal derivatives of neural crest, present	1	1	1	1	1	?	?	?	1	1	1	?	1	0	?	?
D	4	0	=>	1	Tripartite brain, present	?	1	1	?	1	?	?	?	?	?	?	?	?	?	?	?
D	14	0	=>	1	Adenohypophysis, present	?	1	1	?	1	?	?	?	?	?	?	?	?	?	?	?
D	33	0	=>	1	Otic capsule, present	?	1	1	1	1	?	1	?	1	?	?	1	?	0	1	0
D	40	0	=>	1	Sensory lines, present	?	1	1	?	1	?	?	?	?	?	?	?	1	?	?	?
D	43	0	=>	1	Internal taste buds, present	?	?	2	?	1	?	?	?	?	?	?	?	?	?	?	?
D	72	0	=>	1	Multi-chambered heart, present	?	1	1	?	1	?	?	?	?	?	?	?	?	?	?	?
D	74	0	=>	1	Circulatory system, closed	?	1	1	?	1	?	?	?	?	?	?	?	?	?	?	?
D	78	0	=>	1	Lymphocytes, present	?	?	1	?	1	?	?	?	?	?	?	?	?	?	?	?
D	94	0	=>	1	Tail, hypochordal	?	1	1	?	1	?	1	1	?	1	?	1	1	?	?	?

O	#		Trans.		Synapomorphies	*Tethymyxine*	*Rubicundus*	*Eptatretus*	*Myxinikela*	*Petromyzon*	*Myxineidus*	*Gilpichthys*	Euconodonta	*Pipiscius*	*Euphanerops*	*Jamoytius*	*Cornovichthys*	*Birkenia*	*Haikouella*	*Haikouichthys*	*Metaspriggina*
D	153	0	=>	1	Braincase with lateral walls, present	?	0	0	0	1	?	?	?	?	1	?	?	?	-	-	0

Cyclostomi, total group

O	#		Trans.		Synapomorphies	*Tethymyxine*	*Rubicundus*	*Eptatretus*	*Myxinikela*	*Petromyzon*	*Myxineidus*	*Gilpichthys*	Euconodonta	*Pipiscius*	*Euphanerops*	*Jamoytius*	*Cornovichthys*	*Birkenia*	*Haikouella*	*Haikouichthys*	*Metaspriggina*
U	20	1	=>	0	Olfactory organs, single	?	0	0	0	0	?	?	?	?	?	?	?	?	?	1	1
U	81	0	=>	1	Body length, longer than five times width or height	2	2	2	1	2	2	1	2	1	1	1	1	0	0	1	0
U	101	0	=>	1	Cellular cartilages with hypertrophied chondrocytes	?	?	1	?	1	?	?	?	?	1	?	?	?	?	?	?
U	102	0	=>	1	Mature chondrocytes, remaining in pairs	?	?	1	?	1	?	?	?	?	1	?	?	?	?	?	?
A	6	0	=>	1	Profundal nerve ganglion, fused with trigeminal ganglion	?	?	1	?	1	?	?	?	?	?	?	?	?	?	?	?
A	8	0	=>	1	Spinal cord, flattened in cross section	?	1	1	?	1	?	?	?	?	?	?	?	?	?	?	?
A	13	0	=>	1	*Pineal opening, exposed	?	-	-	?	1	?	?	?	?	?	?	?	1	?	?	?
A	17	0	=>	1	Nasohypophyseal opening, dorsal	0	0	0	0	1	?	?	?	?	1	1	?	1	?	0	0
A	37	0	=>	1	Statolith, composed of calcium phosphate	?	?	1	?	1	?	?	?	?	?	?	?	?	?	?	?
A	55	0	=>	1	Branchial openings, closely packed	?	0	1	?	0	?	1	1	1	0	0	0	1	0	0	0
A	104	0	=>	1	*‡Spherical/globular dentine, present	-	-	-	-	-	-	-	1	-	-	?	-	1	-	-	-
A	107	0	=>	1	*Dentine, non-polarized connections of tubules/canaliculi	-	-	-	-	-	-	-	1	-	-	?	-	-	-	-	-
A	119	0	=>	1	*Scale, rod-shaped	-	-	-	-	-	-	-	-	-	-	-	-	1	-	-	-
A	121	0	=>	1	*‡Triradiate postbranchial spines, present	-	-	-	-	-	-	-	-	-	-	0	-	1	-	-	-
A	123	0	=>	1	*Median dorsal ridge scales, hooked	-	-	-	-	-	-	-	-	-	-	-	-	1	-	-	-
A	125	0	=>	1	*Scale, with visceral rib	-	-	-	-	-	-	-	-	-	-	-	-	1	-	-	-
A	130	0	=>	2	*Dermal head covering, multiple macromeric plates	-	-	-	-	-	-	-	-	-	-	-	-	2	-	-	-
A	140	0	=>	1	Tooth rows, with transverse bite	1	1	1	?	1	1	1	1	-	-	-	?	-	-	-	-
A	147	0	=>	1	Trematic ring elements, present	?	1	1	?	1	?	?	?	?	?	?	?	?	0	0	0
D	13	0	=>	1	*Pineal opening, exposed	?	-	-	?	1	?	?	?	?	?	?	?	1	?	?	?
D	104	0	=>	1	*‡Spherical/globular dentine, present	-	-	-	-	-	-	-	1	-	-	?	-	1	-	-	-

Cyclostomi (crown group) + *Pipiscius*

O	#		Trans.		Synapomorphies	*Tethymyxine*	*Rubicundus*	*Eptatretus*	*Myxinikela*	*Petromyzon*	*Myxineidus*	*Gilpichthys*	Euconodonta	*Pipiscius*	*Euphanerops*	*Jamoytius*	*Cornovichthys*	*Birkenia*	*Haikouella*	*Haikouichthys*	*Metaspriggina*
U	138	0	=>	1	*Perioral feeding elements, arranged discontinuously	-	-	-	-	1	-	-	-	1	-	-	-	0	-	-	-

O	#		Trans.		Synapomorphies	*Tethymyxine*	*Rubicundus*	*Eptatretus*	*Myxinikela*	*Petromyzon*	*Myxineidus*	*Gilpichthys*	Euconodonta	*Pipiscius*	*Euphanerops*	*Jamoytius*	*Cornovichthys*	*Birkenia*	*Haikouella*	*Haikouichthys*	*Metaspriggina*
A	96	1	=>	0	*Bone, absent	-	-	-	-	-	-	-	0	-	0	?	-	1	-	-	-
A	111	0	=>	1	*‡Mineralized endoskeleton, present	-	-	-	-	-	-	-	1	-	1	0	-	0	-	-	-
A	112	1	=>	0	*Mineralized exoskeleton, absent	-	-	-	-	-	-	-	0	-	0	1	-	1	-	-	-
A	127	0	=>	2	*‡Odontodes, present in pharynx	-	-	-	-	-	-	-	2	-	-	-	-	0	-	-	-
A	150	1	=>	0	Axial skeletons around notochord (centra), absent	?	0	0	?	0	?	?	?	?	1	?	?	?	?	1	?
D	55	0	=>	1	Branchial openings, closely packed	?	0	1	?	0	?	1	1	1	0	0	0	1	0	0	0
D	58	1	=>	0	Number of branchial arches, four or five	2	0	3	0	1	?	1	0	0	-	3	3	2	1	1	1

Cyclostomi (crown group) + Euconodonta

O	#		Trans.		Synapomorphies	*Tethymyxine*	*Rubicundus*	*Eptatretus*	*Myxinikela*	*Petromyzon*	*Myxineidus*	*Gilpichthys*	Euconodonta	*Pipiscius*	*Euphanerops*	*Jamoytius*	*Cornovichthys*	*Birkenia*	*Haikouella*	*Haikouichthys*	*Metaspriggina*
U	86	0	=>	1	Fin along dorsal midline, originating posterior to anal vent	1	1	1	1	1	?	?	1	2	2	?	2	2	?	0	?
U	139	2	=>	1	Denticulate/cuspidate elements, within buccal cavity	1	1	1	?	1	1	1	1	0	0	0	?	0	0	0	0
A	60	1	=>	0	Branchial openings at similar horizontal level	?	0	0	?	1	?	0	?	1	1	?	1	1	0	?	?
A	142	0	=>	1	A pulley-like feeding apparatus of protractors and retractors	1	1	1	?	1	?	?	?	0	?	?	?	?	?	0	0
D	140	0	=>	1	Tooth rows, with transverse bite	1	1	1	?	1	1	1	1	-	-	-	?	-	-	-	-

Cyclostomi, crown group

O	#		Trans.		Synapomorphies	*Tethymyxine*	*Rubicundus*	*Eptatretus*	*Myxinikela*	*Petromyzon*	*Myxineidus*	*Gilpichthys*	Euconodonta	*Pipiscius*	*Euphanerops*	*Jamoytius*	*Cornovichthys*	*Birkenia*	*Haikouella*	*Haikouichthys*	*Metaspriggina*
U	141	0	=>	1	Perioral/buccal feeding structure, keratinous	1	1	1	?	1	?	?	0	?	0	0	?	?	-	-	-
U	161	0	=>	1	Postotic myomeres, shifted anteriorly to periocular position	?	1	1	?	1	?	?	0	?	?	?	?	?	?	?	0
D	6	0	=>	1	Profundal nerve ganglion, fused with trigeminal ganglion	?	?	1	?	1	?	?	?	?	?	?	?	?	?	?	?
D	8	0	=>	1	Spinal cord, flattened in cross section	?	1	1	?	1	?	?	?	?	?	?	?	?	?	?	?
D	37	0	=>	1	Statolith, composed of calcium phosphate	?	?	1	?	1	?	?	?	?	?	?	?	?	?	?	?
D	142	0	=>	1	A pulley-like feeding apparatus of protractors and retractors	1	1	1	?	1	?	?	?	0	?	?	?	?	?	0	0
D	147	0	=>	1	Trematic ring elements, present	?	1	1	?	1	?	?	?	?	?	?	?	?	0	0	0
D	150	1	=>	0	Axial skeletons around notochord (centra), absent	?	0	0	?	0	?	?	?	?	1	?	?	?	?	1	?
D	160	0	=>	1	Male gametes, shed directly through coelom	?	1	1	?	1	?	?	?	?	?	?	?	?	?	?	?

O	#		Trans.		Synapomorphies	*Tethymyxine*	*Rubicundus*	*Eptatretus*	*Myxinikela*	*Petromyzon*	*Myxineidus*	*Gilpichthys*	Euconodonta	*Pipiscius*	*Euphanerops*	*Jamoytius*	*Cornovichthys*	*Birkenia*	*Haikouella*	*Haikouichthys*	*Metaspriggina*
**Myxinoidea, total group**																					
U	32	0	=>	1	Eyes, positioned dorsally	?	1	1	1	0	?	0	0	0	?	0	?	0	0	0	0
U	45	0	=>	2	Branchial apparatus, positioned posteriorly	2	2	2	2	0	0	0	-	1	1	1	0	2	-	0	0
U	153	1	=>	0	Braincase, lateral wall absent	?	0	0	0	1	?	?	?	?	1	?	?	?	-	-	0
U	157	0	=>	1	Barbels supported by cartilages, present	1	1	1	1	0	0	0	?	0	0	0	0	0	0	0	0
A	10	1	=>	0	Roots of spinal nerve, originating intersegmentally	?	0	0	?	1	?	?	?	?	?	?	?	?	?	?	?
A	11	1	=>	0	Mauthner fibers at rhombomere 4, absent	?	0	0	?	1	?	?	?	?	?	?	?	?	?	?	?
A	12	1	=>	0	Pineal organ, absent	?	0	0	?	1	?	?	?	?	?	?	?	?	?	?	?
A	17	1	=>	0	Nasohypophyseal opening, terminal	0	0	0	0	1	?	?	?	?	1	1	?	1	?	0	0
A	18	0	=>	1	Nasohypophyseal canal, opening into pharynx	1	1	1	?	0	?	?	?	?	0	?	?	?	?	0	0
A	28	1	=>	0	Extrinsic eye musculature, absent	?	0	0	?	1	?	?	?	?	?	?	?	?	?	?	?
A	35	0	=>	1	Ant. + post. semicircular canals, fused at half a loop	?	1	1	?	0	?	?	?	?	?	?	?	?	?	?	?
A	39	1	=>	0	Electroreceptive cells, absent	?	0	0	?	1	?	?	?	?	?	?	?	?	?	?	?
A	41	1	=>	0	Sensory lines, on head only	?	0	0	?	1	?	?	?	?	?	?	?	1	?	?	?
A	43	1	=>	2	Internal taste buds, replaced by Schreiner organs	?	?	2	?	1	?	?	?	?	?	?	?	?	?	?	?
A	64	0	=>	1	Mouth, subterminal	1	1	1	?	0	?	0	?	0	1	1	?	0	1	1	1
A	68	0	=>	1	Velar cartilages, extended posteriorly beyond hyoid arch	1	1	1	?	0	?	?	?	?	?	?	?	?	?	?	?
A	73	1	=>	0	Pericardium, not closed by skeleton	?	0	0	?	1	?	?	?	?	?	?	?	?	?	?	?
A	75	0	=>	1	Massive subcutaneous sinus, present	?	1	1	?	0	?	?	?	?	?	?	?	?	?	?	?
A	76	0	=>	1	Dorsal aortae, paired	?	1	1	?	0	?	?	?	?	?	?	?	?	?	?	?
A	149	1	=>	0	Axial skeleton around dorsal nerve cord, absent	?	0	0	?	1	?	?	?	?	1	?	?	?	?	?	?
A	158	0	=>	1	Forked subnasal cartilage, present	1	1	1	?	0	?	?	?	?	0	?	?	?	?	?	?
A	159	1	=>	0	Tectal cartilages, absent	0	0	0	?	1	?	?	?	?	1	?	?	?	?	?	?
A	163	1	=>	0	Digestive tract, following pharynx	0	0	0	?	1	?	?	?	?	1	?	?	?	0	?	0
**Myxinoidea, crown group**																					
U	27	0	=>	1	Eyes, covered by dermis	?	1	1	0	0	?	0	0	0	0	0	0	0	0	0	0

O	#		Trans.		Synapomorphies	*Tethymyxine*	*Rubicundus*	*Eptatretus*	*Myxinikela*	*Petromyzon*	*Myxineidus*	*Gilpichthys*	Euconodonta	*Pipiscius*	*Euphanerops*	*Jamoytius*	*Cornovichthys*	*Birkenia*	*Haikouella*	*Haikouichthys*	*Metaspriggina*
U	81	1	=>	2	Body length, greater than ten times width or height	2	2	2	1	2	2	1	2	1	1	1	1	0	0	1	0
U	90	0	=>	1	Preanal skin fold, present	1	1	1	0	0	?	0	0	0	1	1	1	1	1	1	0
U	165	0	=>	1	Globular slime glands, present	1	1	1	0	0	?	0	?	0	0	?	?	?	0	0	0
A	47	0	=>	1	Prebranchial length, a quarter of body length from snout	1	1	2	0	-	-	-	-	-	-	-	-	0	-	-	-
D	9	1	=>	0	Dorsal and ventral roots of spinal nerves, united	?	1	1	?	0	?	?	?	?	?	?	?	?	?	?	?
D	11	1	=>	0	Mauthner fibers at rhombomere 4, absent	?	0	0	?	1	?	?	?	?	?	?	?	?	?	?	?
D	12	1	=>	0	Pineal organ, absent	?	0	0	?	1	?	?	?	?	?	?	?	?	?	?	?
D	18	0	=>	1	Nasohypophyseal canal, opening into pharynx	1	1	1	?	0	?	?	?	?	0	?	?	?	?	0	0
D	28	1	=>	0	Extrinsic eye musculature, absent	?	0	0	?	1	?	?	?	?	?	?	?	?	?	?	?
D	35	0	=>	1	Ant. + post. semicircular canals, fused at half a loop	?	1	1	?	0	?	?	?	?	?	?	?	?	?	?	?
D	41	1	=>	0	Sensory lines, on head only	?	0	0	?	1	?	?	?	?	?	?	?	1	?	?	?
D	60	1	=>	0	Branchial openings at similar horizontal level	?	0	0	?	1	?	0	?	1	1	?	1	1	0	?	?
D	64	0	=>	1	Mouth, subterminal	1	1	1	?	0	?	0	?	0	1	1	?	0	1	1	1
D	68	0	=>	1	Velar cartilages, extended posteriorly beyond hyoid arch	1	1	1	?	0	?	?	?	?	?	?	?	?	?	?	?
D	73	1	=>	0	Pericardium, not closed by skeleton	?	0	0	?	1	?	?	?	?	?	?	?	?	?	?	?
D	75	0	=>	1	Massive subcutaneous sinus, present	?	1	1	?	0	?	?	?	?	?	?	?	?	?	?	?
D	76	0	=>	1	Dorsal aortae, paired	?	1	1	?	0	?	?	?	?	?	?	?	?	?	?	?
D	149	1	=>	0	Axial skeleton around dorsal nerve cord, absent	?	0	0	?	1	?	?	?	?	1	?	?	?	?	?	?
D	158	0	=>	1	Forked subnasal cartilage, present	1	1	1	?	0	?	?	?	?	0	?	?	?	?	?	?
D	159	1	=>	0	Tectal cartilages, absent	0	0	0	?	1	?	?	?	?	1	?	?	?	?	?	?
D	163	1	=>	0	Digestive tract, following pharynx	0	0	0	?	1	?	?	?	?	1	?	?	?	0	?	0

Tethymyxine* + *Rubicundus

U	21	0	=>	1	Nasohypophyseal canal, tapers anteriorly	1	1	0	0	-	-	-	-	-	-	-	-	-	-	-	-
U	23	0	=>	1	Nasohypophyseal barbels, originating behind aperture	1	1	0	0	-	-	-	-	-	-	-	-	-	-	-	-
U	55	1	=>	0	Branchial openings, spaced accordingly with pouches	?	0	1	?	0	?	1	1	1	0	0	0	1	0	0	0
A	22	0	=>	1	Nasohypophyseal canal, tubular extension	0	1	0	0	-	-	-	-	-	-	-	-	-	-	-	-

O	#	Trans.			Synapomorphies	*Tethymyxine*	*Rubicundus*	*Eptatretus*	*Myxinikela*	*Petromyzon*	*Myxineidus*	*Gilpichthys*	Euconodonta	*Pipiscius*	*Euphanerops*	*Jamoytius*	*Cornovichthys*	*Birkenia*	*Haikouella*	*Haikouichthys*	*Metaspriggina*
A	24	0	=>	1	Nasohypophyseal papillae, ventral element present	?	1	1	?	-	-	-	-	-	-	-	-	-	-	-	-
A	166	0	=>	1	Number of slime glands, substantially greater than 100	1	X	0	-	-	?	-	?	-	-	?	?	?	-	-	-
D	47	0	=>	1	Prebranchial length, a quarter of body length from snout	1	1	2	0	-	-	-	-	-	-	-	-	0	-	-	-

Tethymyxine

O	#	Trans.			Synapomorphies	*Tethymyxine*	*Rubicundus*	*Eptatretus*	*Myxinikela*	*Petromyzon*	*Myxineidus*	*Gilpichthys*	Euconodonta	*Pipiscius*	*Euphanerops*	*Jamoytius*	*Cornovichthys*	*Birkenia*	*Haikouella*	*Haikouichthys*	*Metaspriggina*
U	58	0	=>	2	Number of branchial pouches, eight to ten	2	0	3	0	1	?	1	0	0	-	3	3	2	1	1	1
A	22	1	=>	0	Nasohypophyseal canal, opening in terminal aperture	0	1	0	0	-	-	-	-	-	-	-	-	-	-	-	-
D	166	0	=>	1	Number of slime glands, substantially greater than 100	1	X	0	-	-	?	-	?	-	-	?	?	?	-	-	-

Eptatretus + Myxine

O	#	Trans.			Synapomorphies	*Tethymyxine*	*Rubicundus*	*Eptatretus*	*Myxinikela*	*Petromyzon*	*Myxineidus*	*Gilpichthys*	Euconodonta	*Pipiscius*	*Euphanerops*	*Jamoytius*	*Cornovichthys*	*Birkenia*	*Haikouella*	*Haikouichthys*	*Metaspriggina*
U	54	0	=>	1	Excurrent branchial ducts, extending posteriorly	?	0	1	?	0	?	?	?	?	0	0	0	1	?	?	?
U	58	0	=>	1	Number of branchial pouches, six or seven	2	0	3	0	1	?	1	0	0	-	3	3	2	1	1	1
A	47	1	=>	2	Prebranchial length, greater than a quarter body length	1	1	2	0	-	-	-	-	-	-	-	-	0	-	-	-
D	43	1	=>	2	Internal taste buds, replaced by Schreiner organs	?	?	2	?	1	?	?	?	?	?	?	?	?	?	?	?
D	47	0	=>	2	Prebranchial length, greater than a quarter body length	1	1	2	0	-	-	-	-	-	-	-	-	0	-	-	-

Myxine + Neomyxine

O	#	Trans.			Synapomorphies	*Tethymyxine*	*Rubicundus*	*Eptatretus*	*Myxinikela*	*Petromyzon*	*Myxineidus*	*Gilpichthys*	Euconodonta	*Pipiscius*	*Euphanerops*	*Jamoytius*	*Cornovichthys*	*Birkenia*	*Haikouella*	*Haikouichthys*	*Metaspriggina*
U	25	1	=>	0	Nasohypophyseal papillae, dorsal elements on midline	?	1	1	?	-	-	-	-	-	-	-	-	-	-	-	-
U	27	1	=>	2	Eyes, covered by trunk muscles	?	1	1	0	0	?	0	0	0	0	0	0	0	0	0	0
U	53	0	=>	1	Single confluent branchial opening, present	0	0	0	0	0	?	0	?	0	0	0	0	0	0	0	0
U	144	1	=>	0	Keratinous tooth plate, anterior element with two fused cusps	?	1	1	?	-	-	?	-	-	-	-	-	-	-	-	-

Petromyzontiformes, total group

O	#	Trans.			Synapomorphies	*Tethymyxine*	*Rubicundus*	*Eptatretus*	*Myxinikela*	*Petromyzon*	*Myxineidus*	*Gilpichthys*	Euconodonta	*Pipiscius*	*Euphanerops*	*Jamoytius*	*Cornovichthys*	*Birkenia*	*Haikouella*	*Haikouichthys*	*Metaspriggina*
U	44	0	=>	2	Preoptic head length, longer than branchial series	0	0	0	1	0	1	2	0	2	0	0	0	0	-	0	0
U	58	0	=>	1	Number of branchial pouches, six or seven	2	0	3	0	1	?	1	0	0	-	3	3	2	1	1	1
A	9	1	=>	0	Roots of spinal nerves, united	?	1	1	?	0	?	?	?	?	?	?	?	?	?	?	?
A	69	0	=>	1	Velar cartilage, functioning as a valve only in adult	0	0	0	?	1	?	?	?	?	?	?	?	?	?	?	?

O	#	Trans.			Synapomorphies	*Tethymyxine*	*Rubicundus*	*Eptatretus*	*Myxinikela*	*Petromyzon*	*Myxineidus*	*Gilpichthys*	Euconodonta	*Pipiscius*	*Euphanerops*	*Jamoytius*	*Cornovichthys*	*Birkenia*	*Haikouella*	*Haikouichthys*	*Metaspriggina*
A	151	1	=>	0	Axial skeletons around dorsal aorta, absent	?	1	1	?	0	?	?	?	?	1	?	?	?	?	?	?
					**Petromyzontiformes (crown group) + *Hardistiella/Mayomyzon***																
U	66	0	=>	1	Palatal shelf, forming dorsal roof of snout	0	0	0	0	1	1	0	0	?	1	?	?	1	?	0	0
U	155	0	=>	1	Annular cartilage, present	0	0	0	0	1	?	0	0	?	1	1	?	?	?	?	?
U	156	0	=>	1	Large oral disc, present	0	0	0	0	1	1	0	0	1	0	0	0	0	0	0	0
A	60	0	=>	1	Branchial openings, forming a posteroventrally inclined row	?	0	0	?	1	?	0	?	1	1	?	1	1	0	?	?
A	145	0	=>	1	Circumoral feeding structures, radially organized	0	0	0	0	1	?	0	0	0	0	0	?	0	0	0	0
D	17	0	=>	1	Nasohypophyseal opening, dorsal	0	0	0	0	1	?	?	?	?	1	1	?	1	?	0	0
					**Petromyzontiformes (crown group) + *Myxineidus***																
U	44	2	=>	1	Preoptic head length, approximately equal to branchial length	0	0	0	1	0	1	2	0	2	0	0	0	0	-	0	0
A	46	0	=>	1	Branchial apparatus, multiple branchial openings, preotic	-	-	-	-	-	-	-	-	0	0	0	-	-	-	-	-
A	55	1	=>	0	Branchial openings, spaced as branchial pouches	?	0	1	?	0	?	1	1	1	0	0	0	1	0	0	0
					**Petromyzontiformes (crown group) + *Priscomyzon***																
U	137	0	=>	1	Perioral denticulate/cuspidate feeding elements, present	0	0	0	0	1	0	0	0	1	0	0	0	1	0	0	0
D	55	1	=>	0	Branchial openings, spaced as branchial pouches	?	0	1	?	0	?	1	1	1	0	0	0	1	0	0	0
D	145	0	=>	1	Circumoral feeding structures, radially organized	0	0	0	0	1	?	0	0	0	0	0	?	0	0	0	0
					**Petromyzontiformes (crown group) + *Mesomyzon***																
U	84	0	=>	1	Distinct dorsal fin, present	0	0	0	0	1	?	0	0	0	0	0	0	0	0	1	0
					**Petromyzontiformes, crown group**																
U	44	1	=>	0	Preoptic head length, shorter than branchial length	0	0	0	1	0	1	2	0	2	0	0	0	0	-	0	0
D	10	0	=>	1	Roots of spinal nerve, originating intrasegmentally	?	0	0	?	1	?	?	?	?	?	?	?	?	?	?	?
D	69	0	=>	1	Velar cartilage, functioning as a valve only in adult	0	0	0	?	1	?	?	?	?	?	?	?	?	?	?	?

O	#		Trans.		Synapomorphies	*Tethymyxine*	*Rubicundus*	*Eptatretus*	*Myxinikela*	*Petromyzon*	*Myxineidus*	*Gilpichthys*	Euconodonta	*Pipiscius*	*Euphanerops*	*Jamoytius*	*Cornovichthys*	*Birkenia*	*Haikouella*	*Haikouichthys*	*Metaspriggina*
D	151	1	=>	0	Axial skeletons around dorsal aorta, absent	?	1	1	?	0	?	?	?	?	1	?	?	?	?	?	?

Anaspida

O	#		Trans.		Synapomorphies	*Tethymyxine*	*Rubicundus*	*Eptatretus*	*Myxinikela*	*Petromyzon*	*Myxineidus*	*Gilpichthys*	Euconodonta	*Pipiscius*	*Euphanerops*	*Jamoytius*	*Cornovichthys*	*Birkenia*	*Haikouella*	*Haikouichthys*	*Metaspriggina*
U	90	0	=>	1	Preanal skin fold, present	1	1	1	0	0	?	0	0	0	1	1	1	1	1	1	0
U	93	0	=>	1	Tail shape, ventral lobe larger than dorsal lobe	0	0	0	0	0	?	0	0	0	1	?	1	1	0	0	0
A	58	0	=>	3	Number of branchial pouches, greater than ten	2	0	3	0	1	?	1	0	0	-	3	3	2	1	1	1
A	66	0	=>	1	Palatal shelf, forming dorsal roof of snout	0	0	0	0	1	1	0	0	?	1	?	?	1	?	0	0
A	105	1	=>	0	*Tubular dentine, absent	-	-	-	-	-	-	-	1	-	-	?	-	0	-	-	-
A	155	0	=>	1	Annular cartilage, present	0	0	0	0	1	?	0	0	?	1	1	?	?	?	?	?
D	58	1	=>	3	Number of branchial pouches, greater than ten	2	0	3	0	1	?	1	0	0	-	3	3	2	1	1	1

Birkenia/Rhyncholepis + Euphanerops/Jamoytius

O	#		Trans.		Synapomorphies	*Tethymyxine*	*Rubicundus*	*Eptatretus*	*Myxinikela*	*Petromyzon*	*Myxineidus*	*Gilpichthys*	Euconodonta	*Pipiscius*	*Euphanerops*	*Jamoytius*	*Cornovichthys*	*Birkenia*	*Haikouella*	*Haikouichthys*	*Metaspriggina*
U	45	0	=>	2	Branchial apparatus, displaced posteriorly	2	2	2	2	0	0	0	-	1	1	1	0	2	-	0	0
U	95	0	=>	1	Mineralized skeletons, present	0	0	0	0	0	0	0	1	0	1	1	0	1	0	0	0
D	66	0	=>	1	Palatal shelf, forming dorsal roof of snout	0	0	0	0	1	1	0	0	?	1	?	?	1	?	0	0
D	91	0	=>	1	Preanal skin fold, paired	0	0	0	-	-	?	-	-	-	1	1	0	1	0	0	-
D	119	0	=>	1	Scale, rod-shaped	-	-	-	-	-	-	-	-	-	-	-	-	1	-	-	-
D	123	0	=>	1	Median dorsal ridge scales, hooked	-	-	-	-	-	-	-	-	-	-	-	-	1	-	-	-

Birkenia + Rhyncholepis

O	#		Trans.		Synapomorphies	*Tethymyxine*	*Rubicundus*	*Eptatretus*	*Myxinikela*	*Petromyzon*	*Myxineidus*	*Gilpichthys*	Euconodonta	*Pipiscius*	*Euphanerops*	*Jamoytius*	*Cornovichthys*	*Birkenia*	*Haikouella*	*Haikouichthys*	*Metaspriggina*
U	58	3	=>	2	Number of branchial pouches, eight to ten	2	0	3	0	1	?	1	0	0	-	3	3	2	1	1	1
U	81	1	=>	0	Body lengths, less than five times height or width	2	2	2	1	2	2	1	2	1	1	1	1	0	0	1	0
U	137	0	=>	1	Perioral feeding elements, present	0	0	0	0	1	0	0	0	1	0	0	0	1	0	0	0
D	54	0	=>	1	Branchial openings, posterior to branchial pouches	?	0	1	?	0	?	?	?	?	0	0	0	1	?	?	?
D	55	0	=>	1	Branchial openings, closely packed	?	0	1	?	0	?	1	1	1	0	0	0	1	0	0	0
D	105	1	=>	0	Tubular dentine, absent	-	-	-	-	-	-	-	1	-	-	?	-	0	-	-	-
D	121	0	=>	1	Triradiate postbranchial spines, present	-	-	-	-	-	-	-	-	-	-	0	-	1	-	-	-

O	#	Trans.			Synapomorphies	*Tethymyxine*	*Rubicundus*	*Eptatretus*	*Myxinikela*	*Petromyzon*	*Myxineidus*	*Gilpichthys*	*Euconodonta*	*Pipiscius*	*Euphanerops*	*Jamoytius*	*Cornovichthys*	*Birkenia*	*Haikouella*	*Haikouichthys*	*Metaspriggina*
D	125	0	=>	1	Scales, with visceral ribs	-	-	-	-	-	-	-	-	-	-	-	-	1	-	-	-
D	130	0	=>	2	Dermal head covering, multiple macromeric plates	-	-	-	-	-	-	-	-	-	-	-	-	2	-	-	-

Lasanius + Euphanerops/Jamoytius

O	#	Trans.			Synapomorphies	*Tethymyxine*	*Rubicundus*	*Eptatretus*	*Myxinikela*	*Petromyzon*	*Myxineidus*	*Gilpichthys*	*Euconodonta*	*Pipiscius*	*Euphanerops*	*Jamoytius*	*Cornovichthys*	*Birkenia*	*Haikouella*	*Haikouichthys*	*Metaspriggina*
U	109	1	=>	0	Enamel/oid, absent	-	-	-	-	-	-	-	1	-	-	0	-	1	-	-	-
U	113	0	=>	1	Mineralized integumentary skeleton, limited coverage	-	-	-	-	-	-	-	-	-	-	1	-	0	-	-	-
U	126	1	=>	0	Oral plates, absent	-	-	-	-	-	-	-	-	-	0	0	-	1	-	-	-
U	128	1	=>	0	Dermal head covering, absent	-	-	-	-	-	-	-	-	-	0	0	-	1	-	-	-
U	133	1	=>	0	Mineralized exoskeletal circumocular elements, absent	-	-	-	-	-	-	-	-	-	-	0	-	1	-	-	-
A	63	0	=>	5	Branchial opening, mineralized exoskeleton locally absent	-	-	-	-	-	-	-	-	-	-	5	-	3	-	-	-
A	103	1	=>	0	Tubular dentine, absent	-	-	-	-	-	-	-	1	-	0	?	-	1	-	-	-
D	63	0	=>	5	Branchial opening, mineralized exoskeleton locally absent	-	-	-	-	-	-	-	-	-	-	5	-	3	-	-	-
D	155	0	=>	1	Annular cartilage, present	0	0	0	0	1	?	0	0	?	1	1	?	?	?	?	?

Euphanerops + Jamoytius

O	#	Trans.			Synapomorphies	*Tethymyxine*	*Rubicundus*	*Eptatretus*	*Myxinikela*	*Petromyzon*	*Myxineidus*	*Gilpichthys*	*Euconodonta*	*Pipiscius*	*Euphanerops*	*Jamoytius*	*Cornovichthys*	*Birkenia*	*Haikouella*	*Haikouichthys*	*Metaspriggina*
U	45	2	=>	1	Branchial apparatus, anterior position (first pouch preoptic)	2	2	2	2	0	0	0	-	1	1	1	0	2	-	0	0
U	64	0	=>	1	Mouth, subterminal	1	1	1	?	0	?	0	?	0	1	1	?	0	1	1	1
A	54	1	=>	0	Branchial openings, lateral to branchial pouches	?	0	1	?	0	?	?	?	?	0	0	0	1	?	?	?
A	55	1	=>	0	Branchial openings, spaced as in branchial pouches	?	0	1	?	0	?	1	1	1	0	0	0	1	0	0	0
A	96	1	=>	0	Bone, absent	-	-	-	-	-	-	-	0	-	0	?	-	1	-	-	-
A	98	1	=>	0	Lamellar aspidin, absent	-	-	-	-	-	-	-	-	-	-	0	-	1	-	-	-
A	100	0	=>	1	Calcified cartilages, present	-	-	-	-	-	-	-	0	-	1	?	-	0	-	-	-
A	121	1	=>	0	Triradiate postbranchial spines, absent	-	-	-	-	-	-	-	-	-	-	0	-	1	-	-	-
A	122	1	=>	0	Median dorsal ridge scales, absent	-	-	-	-	-	-	-	-	-	-	0	-	1	-	-	-

Gnathostomata, total group

O	#	Trans.			Synapomorphies	*Tethymyxine*	*Rubicundus*	*Eptatretus*	*Myxinikela*	*Petromyzon*	*Myxineidus*	*Gilpichthys*	*Euconodonta*	*Pipiscius*	*Euphanerops*	*Jamoytius*	*Cornovichthys*	*Birkenia*	*Haikouella*	*Haikouichthys*	*Metaspriggina*
U	15	0	=>	1	Olfactory peduncles, present	?	0	0	?	0	?	?	?	?	?	?	?	?	?	0	0

O	#		Trans.		Synapomorphies	*Tethymyxine*	*Rubicundus*	*Eptatretus*	*Myxinikela*	*Petromyzon*	*Myxineidus*	*Gilpichthys*	Euconodonta	*Pipiscius*	*Euphanerops*	*Jamoytius*	*Cornovichthys*	*Birkenia*	*Haikouella*	*Haikouichthys*	*Metaspriggina*
U	93	0	=>	2	Tail shape, dorsal lobe larger than ventral lobe	0	0	0	0	0	?	0	0	0	1	?	1	1	0	0	0
U	95	0	=>	1	Mineralized skeletons, present	0	0	0	0	0	0	0	1	0	1	1	0	1	0	0	0
U	137	0	=>	1	Perioral feeding structures, present	0	0	0	0	1	0	0	0	1	0	0	0	1	0	0	0
A	5	0	=>	1	Cerebellum with corpus cerebelli, present	?	0	0	?	0	?	?	?	?	?	?	?	?	?	?	?
A	7	0	=>	1	Facial nerve, tripartite division	?	0	0	?	0	?	?	?	?	?	?	?	?	?	?	?
A	29	0	=>	1	Extrinsic ocular muscles innervated by CN III, four	?	-	-	?	0	?	?	?	?	?	?	?	?	?	?	?
A	34	0	=>	1	Vertical semicircular canals forming loops, present	?	0	0	?	0	?	?	?	?	?	?	?	?	-	?	-
A	58	0	=>	2	Number of branchial pouches, eight to ten	2	0	3	0	1	?	1	0	0	-	3	3	2	1	1	1
A	63	0	=>	1	Branchial openings, demarcated by single element dorsally	-	-	-	-	-	-	-	-	-	-	5	-	3	-	-	-
A	77	0	=>	1	Lateral head vein, continuous with anterior cardinal vein	?	0	0	?	0	?	?	?	?	?	?	?	?	?	?	?
A	79	0	=>	1	Lymphocytes antigen receptors, T and B	?	0	0	?	0	?	?	?	?	?	?	?	?	?	?	?
A	80	0	=>	1	Subaponeurotic vascular plexus, present	?	0	0	?	0	?	?	?	?	?	?	?	?	?	?	?
A	115	0	=>	1	Exoskeleton, superficial layer of tubular organization	-	-	-	-	-	-	-	-	-	-	?	-	0	-	-	-
A	154	0	=>	1	Occiput enclosing vagus and glossopharyngeal nerves, present	?	0	0	0	0	?	?	?	?	?	?	?	?	0	0	0
A	160	1	=>	0	Male gametes, transmitted through duct	?	1	1	?	1	?	?	?	?	?	?	?	?	?	?	?
D	5	0	=>	1	Cerebellum with corpus cerebelli, present	?	0	0	?	0	?	?	?	?	?	?	?	?	?	?	?
D	34	0	=>	1	Vertical semicircular canals forming loops, present	?	0	0	?	0	?	?	?	?	?	?	?	?	-	?	-
D	58	1	=>	2	Number of branchial pouches, eight to ten	2	0	3	0	1	?	1	0	0	-	3	3	2	1	1	1
D	80	0	=>	1	Subaponeurotic vascular plexus, present	?	0	0	?	0	?	?	?	?	?	?	?	?	?	?	?
D	115	0	=>	1	Exoskeleton, superficial layer of tubular organization	-	-	-	-	-	-	-	-	-	-	?	-	0	-	-	-

Table S4. Comparison of character support for cyclostomes in morphological cladistic datasets. Left column lists unambiguous, ACCTRAN-, or DELTRAN-optimized synapomorphies of total- and crown-group cyclostomes under maximum parsimony. Bold, italic typeface indicates synapomorphies supported in the analysis presented in this paper. Each remaining column represents a dataset. All datasets except our own were given a topological constraint to produce the cyclostome clade.

Key insights provided by this comparison are: (***a***) much of the character support for cyclostomes as recovered in our analysis exists in previous datasets; and (***b***) this support — hence the clade — is recovered in our analysis after treatment of non-independent character coding. Our analysis did not find congruence for many character transformations optimized as cyclostome synapomorphies in the constrained analyses of previous datasets. Most of these transformations fall into one of the following categories: symplesiomorphic to cyclostomes in our analysis (§); optimized to internal nodes within cyclostomes in our analysis (°); optimized to a reverse character polarity in our analysis (**); not included in our analysis due to uninformative nature (‡). These categories are consequences of increased sampling and increased inapplicable coding (to accommodate character non-independence) (see Part D for detailed discussion).

Methods: Tree constraint was used to enforce the cyclostome topology. For each dataset, a heuristic search was run (per original analysis) to obtain strict consensus of most parsimonious trees. From this, tree constraint designated: (***i***) hagfish and lampreys to form a clade; (***ii***) any taxon resolved on the hagfish or lamprey stem in the unconstrained strict consensus tree to fall within the cyclostome crown; (***iii***) any taxon resolved on the cyclostome stem in the unconstrained strict consensus tree to be unconstrained topologically. A heuristic search was run again under this constraint, and most parsimonious character changes were mapped onto strict consensus of the topologically constrained shortest trees under both ACCTRAN and DELTRAN. Character coding or taxonomic composition was not altered. All analyses were conducted in PAUP.

Table annotations:
+=Transformation is optimized as a synapomorphy of the crown or total group Cyclostomi.
++= Transformation is optimized as a synapomorphy of the crown or total group Cyclostomi, but is coded by more than one character in the original source.
±=Transformation is optimized as a synapomorphy of the crown or total group Cyclostomi, but the character is a composite in that analysis (coded by more than one character in our analysis).
*=Character is included in our new dataset, but a specific transformation is optimized to a different node.
**=Character is included in our new dataset, and a specific transformation is optimized to a different node in reverse direction due to correction of non-independent coding. Example: Cerebellum with corpus cerebelli is absent in hagfish and lampreys and present in crown-group gnathostomes (the character was coded as inapplicable to non-vertebrate outgroups). Most parsimonious transformation is from absence to presence on the gnathostome stem, not from presence to absence in the cyclostome stem.
°=Character transformation is optimized to an internal node within the cyclostome crown (or subject to artifacts of non-independent coding; see the legend for Table S3)
§=Symplesiomorphies to cyclostomes in our analysis (a specific transformation is optimized somewhere between the root of the tree and the crown vertebrate node).
†=Character is modified in our dataset.
‡=Character is not included in our dataset.
^{N}=New character in our analysis.

**Synapomorphies**	**This study**	**Keating & Donoghue, 2016**	**Gabbott et al., 2016**	**McCoy et al., 2016**	**Conway Morris & Caron, 2014**	**Heimberg et al., 2010**	**Sansom et al., 2010**	**Turner et al., 2010**	**Khonsari et al., 2009**	**Gess et al., 2006**	**Donoghue & Smith, 2001**	**Donoghue et al., 2000**	**Janvier, 1996**
**Nervous system and sensory capsules**													
Olfactory organs, single	+												++
Profundal nerve ganglion, fused with trigeminal ganglion	+					+			+				
Spinal cord, flattened in cross section	+	+	+	+	+	+	+		+	+	+	+	
Nasohypophyseal opening, dorsal	+					+							
Statolith, composed of calcium phosphate	+					+							
**Cerebellum with corpus cerebelli, absent													+
*§Pineal organ, uncovered												+	
*§Adenohypophysis (or nasohypophyseal canal), present				+	+								
*§Eyes with retinal epithelium				+	+								
*§Sensory lines, present				+	+								
*§Sensory lines, absent													+
*Electroreceptors, absent									+				
†§Semicircular canals, present				+	+								
†°Semicircular canals, no distinction between two loops													+
‡Olfactory tract, absent													+
‡Oligodendrocytes, absent						+			+				
‡Optic tectum, present				+	+								
‡Single glossopharyngeal ganglion, present						+							
‡Saccus vasculosus, absent						+							

Synapomorphies	This study	Keating & Donoghue, 2016	Gabbott et al., 2016	McCoy et al., 2016	Conway Morris & Caron, 2014	Heimberg et al., 2010	Sansom et al., 2010	Turner et al., 2010	Khonsari et al., 2009	Gess et al., 2006	Donoghue & Smith, 2001	Donoghue et al., 2000	Janvier, 1996
‡Nasohypophyseal canal, present									+				
**Pharyngeal system**													
[N]***Branchial openings, closely packed***	+												
*Branchial series, posterior to otic capsule		+		+									
*§Branchial lamellae, medial		+	+		+	+	+			+	+	+	
*§Velum, present					+	+	+			+	+	+	
†§Branchial pouches, present					+	+							+
†°Branchial series, fewer than ten arches							+			+			
**Circulatory system**													
*§Circulatory system, closed				+									
*§Lymphocytes, present				+	+								
‡Blood pressure, high				+									
**Fins**													
[N]***Body length, longer than five times width or height***	+												
Fin along dorsal midline, originating posterior to anal vent	+												
*Separate dorsal fin, present								+					
*Tail, no distinct lobes developed			+										

**Synapomorphies**	**This study**	**Keating & Donoghue, 2016**	**Gabbott et al., 2016**	**McCoy et al., 2016**	**Conway Morris & Caron, 2014**	**Heimberg et al., 2010**	**Sansom et al., 2010**	**Turner et al., 2010**	**Khonsari et al., 2009**	**Gess et al., 2006**	**Donoghue & Smith, 2001**	**Donoghue et al., 2000**	**Janvier, 1996**
*Tail, hypochordal				+		+							
*Fin rays, absent								+					
**Skeletons**													
Cellular cartilages with hypertrophied chondrocytes	+		±		±		±			±			
Mature chondrocytes, remaining in pairs	+		±		±		±			±			
Tooth rows, with transverse bite	+	+	+	+	+	+	+			+			+
Perioral/buccal feeding structure, keratinous	+	+	+	+	+	+	+			+	+	+	+
A pulley-like feeding apparatus of protractors and retractors	+				++	+	+			++	+	+	+
Trematic ring elements, present	+	+	+	+	+	+	+				+	+	
[N]***Axial skeletons around notochord (centra), absent***	+												
**Braincase, enclosing glossopharyngeal and vagal ganglia													+
*§Pharyngeal skeleton, fused to neurocranium				+	+	+		+		+			
†§Synthesis of creatine phosphatase, present					+								
†§Braincase, dorsally not closed													+
†°Acellular dermal bone, absent													+
†°Circumoral teeth (or oral plates), present				+		+							+
‡Skeleton of mandibular arch, present				+	+								
‡Skeleton of mandibular arch, differentiated					+								

**Synapomorphies**	**This study**	**Keating & Donoghue, 2016**	**Gabbott et al., 2016**	**McCoy et al., 2016**	**Conway Morris & Caron, 2014**	**Heimberg et al., 2010**	**Sansom et al., 2010**	**Turner et al., 2010**	**Khonsari et al., 2009**	**Gess et al., 2006**	**Donoghue & Smith, 2001**	**Donoghue et al., 2000**	**Janvier, 1996**
**Miscellaneous**													
Postotic myomeres, shifted anteriorly to periocular position	+	+	+		+	+	+			+	+	+	
Male gametes, shed directly through coelom	+			+	+	+	+			+	+	+	+
‡Endocrine and exocrine pancreas, separate						+							
‡Larval phase, present													+

References for Supplementary Appendix

1. Sansom RS, Gabbott SE, Purnell MA (2011) Decay of vertebrate characters in hagfish and lamprey (Cyclostomata) and the implications for the vertebrate fossil record. *Proc R Soc Lond B Biol Sci* 278(1709):1150–1157.

2. Sansom RS, Gabbott SE, Purnell MA (2013) Atlas of vertebrate decay: A visual and taphonomic guide to fossil interpretation. *Palaeontology* 56(3):457–474.

3. Chang M, Zhang J, Miao D (2006) A lamprey from the Cretaceous Jehol biota of China. *Nature* 441(7096):972–974.

4. Chang M, Wu F, Miao D, Zhang J (2014) Discovery of fossil lamprey larva from the Lower Cretaceous reveals its three-phased life cycle. *Proc Natl Acad Sci* 111(43):15486–15490.

5. Hückel U (1970) Die Fischschiefer von Haqel und Hjoula in der Oberkreide des Libanon. *Neues Jahrb Für Geol Paläontol Abh* 135(2):113–149.

6. Forey PL, Yi L, Patterson C, Davies CE (2003) Fossil fishes from the Cenomanian (Upper Cretaceous) of Namoura, Lebanon. *J Syst Palaeontol* 1(4):227–330.

7. Parry LA, et al. (2018) Soft-bodied fossils are not simply rotten carcasses – Toward a holistic understanding of exceptional fossil preservation. *BioEssays* 40(1):1700167.

8. Lüning S, et al. (2004) Integrated depositional model for the Cenomanian–Turonian organic-rich strata in North Africa. *Earth-Sci Rev* 64(1):51–117.

9. Martini FH (1998) The ecology of hagfishes. *The Biology of Hagfishes*, eds Jørgensen JM, Lomholt JP, Weber RE, Malte H (Chapman, London), pp 57–77.

10. Feldmann RM, Grande L, Birkhimer CP, Hannibal JT, McCoy DL (1981) Decapod fauna of the Green River Formation (Eocene) of Wyoming. *J Paleontol* 55(4):788–799.

11. Lim J-D, Martin L (2002) A new fossil mustelid from the Miocene of South Dakota, USA. *Naturwissenschaften* 89(6):270–274.

12. Bergmann U, Manning PL, Wogelius RA (2012) Chemical mapping of paleontological and archeological artifacts with synchrotron X-rays. *Annu Rev Anal Chem* 5(1):361–389.

13. Schneider CA, Rasband WS, Eliceiri KW (2012) NIH Image to ImageJ: 25 years of image analysis. *Nat Methods* 9:671–675.

14. Solé VA, Papillon E, Cotte M, Walter P, Susini J (2007) A multiplatform code for the analysis of energy-dispersive X-ray fluorescence spectra. *Spectrochim Acta Part B At Spectrosc* 62(1):63–68.

15. Ravel B, Newville M (2005) ATHENA, ARTEMIS, HEPHAESTUS: data analysis for X-ray absorption spectroscopy using IFEFFIT. *J Synchrotron Radiat* 12(4):537–541.

16. Chiu K-H, Mok H-K (2011) Study on the accumulation of heavy metals in shallow-water and deep-sea hagfishes. *Arch Environ Contam Toxicol* 60(4):643–653.

17. Glover CN, Blewett TA, Wood CM (2015) Novel route of toxicant exposure in an ancient extant vertebrate: Nickel uptake by hagfish skin and the modifying effects of slime. *Environ Sci Technol* 49(3):1896–1902.

18. Kennedy JF (1986) *Carbohydrate Chemistry: Macrmolecules* (Royal Society of Chemistry, London).

19. Løvtrup S (1977) *The Phylogeny of the Vertebrata* (Wiley, New York).

20. Janvier P (1981) The phylogeny of the Craniata, with particular reference to the significance of fossil "agnathans." *J Vert Paleontol* 1(2):121–159.

21. Janvier P (1996) The dawn of vertebrates: Characters versus common ascent in the rise of current vertebrate phylogenies. *Palaeontology* 39:259–287.

22. Janvier P (2007) Homologies and evolutionary transitions in early vertebrate history. *Major Transitions in Vertebrate Evolution*, eds Anderson JS, Sues H-D (Indiana University Press, Bloomington), pp 57–121.

23. Donoghue PCJ, Forey PL, Aldridge RJ (2000) Conodont affinity and chordate phylogeny. *Biol Rev* 75(02):191–251.

24. Maisey JG (1986) Heads and tails: A chordate phylogeny. *Cladistics* 2(4):201–256.

25. Gagnier P-Y (1993) *Sacabambaspis janvieri*, vertébré ordovicien de Bolivie. 2. Analyse phylogénétique. *Ann Paléontol Vertébrés* 79:119–166.

26. Gess RW, Coates MI, Rubidge BS (2006) A lamprey from the Devonian period of South Africa. *Nature* 443(7114):981–984.

27. Turner S, et al. (2010) False teeth: Conodont-vertebrate phylogenetic relationships revisited. *Geodiversitas* 32(4):545–594.

28. Sansom RS, Freedman K, Gabbott SE, Aldridge RJ, Purnell MA (2010) Taphonomy and affinity of an enigmatic Silurian vertebrate, *Jamoytius kerwoodi* White. *Palaeontology* 53(6):1393–1409.

29. Khonsari RH, Li B, Vernier P, Northcutt RG, Janvier P (2009) Agnathan brain anatomy and craniate phylogeny. *Acta Zool* 90:52–68.

30. Forey PL (1984) Yet more reflections on agnathan-gnathostome relationships. *J Vert Paleontol* 4(3):330–343.

31. Hardisty MW (1982) Lampreys and hagfishes: Analysis of cyclostome relationships. *The Biology of Lampreys*, eds Hardisty MW, Potter IC (Academic Press, New York), pp 165–259.

32. Gabbott SE, et al. (2016) Pigmented anatomy in Carboniferous cyclostomes and the evolution of the vertebrate eye. *Proc R Soc B* 283(1836):20161151.

33. Keating JN, Donoghue PCJ (2016) Histology and affinity of anaspids, and the early evolution of the vertebrate dermal skeleton. *Proc R Soc B* 283(1826):20152917.

34. Heimberg AM, Cowper-Sal·lari R, Sémon M, Donoghue PCJ, Peterson KJ (2010) microRNAs reveal the interrelationships of hagfish, lampreys, and gnathostomes and the nature of the ancestral vertebrate. *Proc Natl Acad Sci* 107(45):19379–19383.

35. Conway Morris S, Caron J-B (2014) A primitive fish from the Cambrian of North America. *Nature* 512(7515):419–422.

36. McCoy VE, et al. (2016) The 'Tully monster' is a vertebrate. *Nature* 532(7600):496–499.

37. Kuratani S, Oisi Y, Ota KG (2016) Evolution of the vertebrate cranium: Viewed from hagfish developmental studies. *Zool Sci* 33(3):229–238.

38. Yalden DW (1985) Feeding mechanisms as evidence for cyclostome monophyly. *Zool J Linn Soc* 84(3):291–300.

39. Oisi Y, Ota KG, Kuraku S, Fujimoto S, Kuratani S (2013) Craniofacial development of hagfishes and the evolution of vertebrates. *Nature* 493(7431):175–180.

40. Ota KG, Kuraku S, Kuratani S (2007) Hagfish embryology with reference to the evolution of the neural crest. *Nature* 446(7136):672–675.

41. Near TJ (2009) Conflict and resolution between phylogenies inferred from molecular and phenotypic data sets for hagfish, lampreys, and gnathostomes. *J Exp Zool B Mol Dev Evol* 312B(7):749–761.

42. Thomson RC, Plachetzki DC, Mahler DL, Moore BR (2014) A critical appraisal of the use of microRNA data in phylogenetics. *Proc Natl Acad Sci* 111(35):E3659–E3668.

43. Kuraku S, Kuratani S (2006) Time scale for cyclostome evolution inferred with a phylogenetic diagnosis of hagfish and lamprey cDNA sequences. *Zool Sci* 23(12):1053–1064.

44. Kuraku S, Hoshiyama D, Katoh K, Suga H, Miyata T (1999) Monophyly of lampreys and hagfishes supported by nuclear DNA–coded genes. *J Mol Evol* 49(6):729–735.

45. Delarbre C, Gallut C, Barriel V, Janvier P, Gachelin G (2002) Complete mitochondrial DNA of the hagfish, *Eptatretus burgeri*: The comparative analysis of mitochondrial DNA sequences strongly supports the cyclostome monophyly. *Mol Phylogenet Evol* 22(2):184–192.

46. Delarbre C, et al. (2000) The complete nucleotide sequence of the mitochondrial DNA of the agnathan *Lampetra fluviatilis*: Bearings on the phylogeny of cyclostomes. *Mol Biol Evol* 17(4):519–529.

47. Stock DW, Whitt GS (1992) Evidence from 18S ribosomal RNA sequences that lampreys and hagfishes form a natural group. *Science* 257(5071):787–789.

48. Mallatt J, Winchell CJ (2007) Ribosomal RNA genes and deuterostome phylogeny revisited: More cyclostomes, elasmobranchs, reptiles, and a brittle star. *Mol Phylogenet Evol* 43(3):1005–1022.

49. Mallatt J, Sullivan J (1998) 28S and 18S rDNA sequences support the monophyly of lampreys and hagfishes. *Mol Biol Evol* 15(12):1706–1718.

50. Takezaki N, Figueroa F, Zaleska-Rutczynska Z, Klein J (2003) Molecular phylogeny of early vertebrates: Monophyly of the agnathans as revealed by sequences of 35 genes. *Mol Biol Evol* 20(2):287–292.

51. Furlong RF, Holland PWH (2002) Bayesian phylogenetic analysis supports monophyly of Ambulacraria and of cyclostomes. *Zool Sci* 19(5):593–599.

52. Winchell CJ, Sullivan J, Cameron CB, Swalla BJ, Mallatt J (2002) Evaluating hypotheses of deuterostome phylogeny and chordate evolution with new LSU and SSU ribosomal DNA data. *Mol Biol Evol* 19(5):762–776.

53. Miyashita T, Coates MI (2016) The embryology of hagfishes and the evolution and development of vertebrates. *Hagfish Biology*, eds Edwards SL, Goss GG (CRC Press, Boca Raton), pp 95–127.

54. Nascimento FF, Reis M dos, Yang Z (2017) A biologist's guide to Bayesian phylogenetic analysis. *Nat Ecol Evol* 1(10):1446–1454.

55. Puttick MN, et al. (2017) Uncertain-tree: Discriminating among competing approaches to the phylogenetic analysis of phenotype data. *Proc R Soc B* 284(1846):20162290.

56. Spencer MR, Wilberg EW (2013) Efficacy or convenience? Model-based approaches to phylogeny estimation using morphological data. *Cladistics* 29(6):663–671.

57. Guillerme T, Cooper N (2016) Effects of missing data on topological inference using a Total Evidence approach. *Mol Phylogenet Evol* 94(Part A):146–158.

58. O'Reilly JE, et al. (2016) Bayesian methods outperform parsimony but at the expense of precision in the estimation of phylogeny from discrete morphological data. *Biol Lett* 12(4):20160081.

59. O'Reilly JE, Puttick MN, Pisani D, Donoghue PCJ (2018) Probabilistic methods surpass parsimony when assessing clade support in phylogenetic analyses of discrete morphological data. *Palaeontology* 61(1):105–118.

60. O'Reilly JE, Puttick MN, Pisani D, Donoghue PCJ (2018) Empirical realism of simulated data is more important than the model used to generate it: a reply to Goloboff et al. *Palaeontology* 61(4):631–635.

61. Puttick MN, Thomas GH, Benton MJ (2016) Dating placentalia: Morphological clocks fail to close the molecular fossil gap. *Evolution* 70(4):873–886.

62. Goloboff PA, Torres A, Arias JS (2018) Weighted parsimony outperforms other methods of phylogenetic inference under models appropriate for morphology. *Cladistics* 34(4):407–437.

63. Goloboff PA, Galvis AT, Arias JS (2018) Parsimony and model-based phylogenetic methods for morphological data: comments on O'Reilly et al. *Palaeontology* 61(4):625–630.

64. Hunt G, Slater G (2016) Integrating paleontological and phylogenetic approaches to macroevolution. *Annu Rev Ecol Evol Syst* 47(1):189–213.

65. Harrison LB, Larsson HCE (2015) Among-character rate variation distributions in phylogenetic analysis of discrete morphological characters. *Syst Biol* 64(2):307–324.

66. Brazeau MD (2011) Problematic character coding methods in morphology and their effects. *Biol J Linn Soc* 104(3):489–498.

67. Swofford DL (2017) *PAUP** (Sinauer Associates).

68. Huelsenbeck J, Ronquist F, Teslenko M (2015) *MrBayes: Bayesian Inference of Phylogeny* Available at: http://mrbayes.sourceforge.net/index.php.

69. Bouckaert R, et al. (2014) BEAST 2: A software platform for Bayesian evolutionary analysis. *PLOS Comput Biol* 10(4):e1003537.

70. Fernholm B, et al. (2013) Hagfish phylogeny and taxonomy, with description of the new genus *Rubicundus* (Craniata, Myxinidae). *J Zool Syst Evol Res* 51(4):296–307.

71. Bardack D (1991) First fossil hagfish (Myxinoidea): A record from the Pennsylvanian of Illinois. *Science* 254(5032):701–703.

72. Gill HS, et al. (2003) Phylogeny of living parasitic lampreys (Petromyzontiformes) based on morphological data. *Copeia* 2003(4):687–703.

73. Renaud CB, Gill HS, Potter IC (2009) Relationships between the diets and characteristics of the dentition, buccal glands and velar tentacles of the adults of the parasitic species of lamprey. *J Zool* 278(3):231–242.

74. Hubbs CL, Potter IC (1971) Distribution, phylogeny and taxonomy. *The Biology of Lampreys. Volume 1.*, eds Hardisty MW, Potter IC (Academic Press, New York), pp 1–65.

75. Potter IC, Gill HS, Renaud CB, Haoucher D (2015) The taxonomy, phylogeny, and distribution of lampreys. *Lampreys: Biology, Conservation and Control*, Fish & Fisheries Series., ed Docker MF (Springer Netherlands), pp 35–73.

76. Renaud CB (2011) *Lampreys of the World. An annotated and illustrated catalogue of lamprey species known to date.* (Food and Agriculture Organization of the United Nations, Rome).

77. Janvier P, Arsenault M (2007) The anatomy of *Euphanerops longaevus* Woodward, 1900, an anaspid-like jawless vertebrate from the Upper Devonian of Miguasha, Quebec, Canada. *Geodiversitas* 29(1):143–216.

78. Sansom RS, Gabbott SE, Purnell MA (2010) Non-random decay of chordate characters causes bias in fossil interpretation. *Nature* 463(7282):797–800.

79. Carlström D (1963) A crystallographic study of vertebrate otoliths. *Biol Bull* 125(3):441–463.

80. Langille RM, Hall BK (1993) Calcification of cartilage from the lamprey *Petromyzon marinus* (L.) in vitro. *Acta Zool* 74(1):31–41.

81. Briggs DEG, Clarkson ENK, Aldridge RJ (1983) The conodont animal. *Lethaia* 16(1):1–14.

82. Krejsa RJ, Bringas P, Slavkin HC (1990) A neontological interpretation of conodont elements based on agnathan cyclostome tooth structure, function, and development. *Lethaia* 23(4):359–378.

83. Aldridge RJ, Donoghue PCJ (1998) Conodonts: A sister group to hagfishes? *The Biology of Hagfishes*, eds Jørgensen JM, Lonholt JP, Weber RE, Malte H (Chapman, London), pp 15–31.

84. Terrill DF, Henderson CM, Anderson JS (2018) New applications of spectroscopy techniques reveal phylogenetically significant soft tissue residue in Paleozoic conodonts. *J Anal At Spectrom* 33(6):992–1002.

85. Sweet WC (1988) *The Conodonta: Morphology, taxonomy, paleoecology, and evolutionary history of a long-extinct animal phylum* (Clarendon Press).

86. Goudemand N, Orchard MJ, Urdy S, Bucher H, Tafforeau P (2011) Synchrotron-aided reconstruction of the conodont feeding apparatus and implications for the mouth of the first vertebrates. *Proc Natl Acad Sci* 108(21):8720–8724.

87. Bardack D, Richardson ES (1977) New agnathous fishes from the Pennsylvanian of Illinois. *Fieldiana Geol* 33(26):489–510.

88. Janvier P (1996) *Early Vertebrates* (Clarendon Press, Oxford).

89. Janvier P (2008) Early jawless vertebrates and cyclostome origins. *Zool Sci* 25(10):1045–1056.

90. Janvier P (2015) Facts and fancies about early fossil chordates and vertebrates. *Nature* 520(7548):483–489.

91. Shu D, et al. (1999) A pipiscid-like fossil from the Lower Cambrian of south China. *Nature* 400(6746):746–749.

92. van der Brugghen G (2017) Taphonomy versus taxonomy and the synonyms of *Euphanerops longaevus* Woodward, 1900 (Agnatha) occurring at the Middle Devonian Achanarras Quarry of Caithness, Scotland. *Neues Jahrb Für Geol Paläontol - Abh* 286(3):329–347.

93. Jandzik D, et al. (2015) Evolution of the new vertebrate head by co-option of an ancient chordate skeletal tissue. *Nature* 518(7540):534–537.

94. Mallatt J, Chen J (2003) Fossil sister group of craniates: Predicted and found. *J Morphol* 258(1):1–31.

95. Hirasawa T, Oisi Y, Kuratani S (2016) *Palaeospondylus* as a primitive hagfish. *Zool Lett* 2:20.

96. Joss J, Johanson Z (2007) Is *Palaeospondylus gunni* a fossil larval lungfish? Insights from Neoceratodus forsteri development. *J Exp Zool B Mol Dev Evol* 308B(2):163–171.

97. Johanson Z, Kearsley A, den Blaauwen J, Newman M, Smith MM (2010) No bones about it: An enigmatic Devonian fossil reveals a new skeletal framework—A potential role of loss of gene regulation. *Semin Cell Dev Biol* 21(4):414–423.

98. Thomson KS (1992) The puzzle of "*Palaeospondylus*." *Am Sci* 80(3):216–219.

99. Johanson Z, Kearsley A, den Blaauwen J, Newman M, Smith MM (2012) Ontogenetic development of an exceptionally preserved Devonian cartilaginous skeleton. *J Exp Zool B Mol Dev Evol* 318B(1):50–58.

100. Miyashita T, Green SA, Bronner ME (in press) Comparative development of cyclostomes. *Evolution and Development of Fishes*, eds Johanson Z, Richter M, Underwood CJ (University of Cambridge Press, Cambridge).

101. Johanson Z, et al. (2017) Questioning hagfish affinities of the enigmatic Devonian vertebrate Palaeospondylus. *R Soc Open Sci* 4(7):170214.

102. Clements T, et al. (2016) The eyes of *Tullimonstrum* reveal a vertebrate affinity. *Nature* 532(7600):500–503.

103. Sallan L, et al. (2017) The 'Tully Monster' is not a vertebrate: Characters, convergence and taphonomy in Palaeozoic problematic animals. *Palaeontology* 60(2):149–157.

104. Poplin C, Sotty D, Janvier P (2001) Un Myxinoïde (Craniata, Hyperotreti) dans le Konservat-Lagerstätte Carbonifère supérieur de Montceau-les-Mines (Allier, France). *Comptes Rendus Académie Sci - Ser IIA - Earth Planet Sci* 332(5):345–350.

105. Maisey JG (1988) Phylogeny of early vertebrate skeletal induction and ossification patterns. *Evolutionary Biology*, Evolutionary Biology., eds Hecht MK, Wallace B, Prance GT (Springer US), pp 1–36.

106. Janvier P (2010) microRNAs revive old views about jawless vertebrate divergence and evolution. *Proc Natl Acad Sci U S A* 107(45):19137–19138.

107. Shimeld SM, Donoghue PCJ (2012) Evolutionary crossroads in developmental biology: cyclostomes (lamprey and hagfish). *Development* 139(12):2091–2099.

108. Kuratani S, Ota KG (2008) Primitive versus derived traits in the developmental program of the vertebrate head: Views from cyclostome developmental studies. *J Exp Zool B Mol Dev Evol* 310B(4):294–314.

109. Sansom RS, Wills MA (2013) Fossilization causes organisms to appear erroneously primitive by distorting evolutionary trees. *Sci Rep* 3. doi:10.1038/srep02545.

110. Sansom RS (2015) Bias and sensitivity in the placement of fossil taxa resulting from interpretations of missing data. *Syst Biol* 64(2):256–266.

111. Miyashita T (2012) *Comparative Analysis of the Anatomy of the Myxinoidea and the Ancestry of Early Vertebrate Lineages*. Unpublished M.Sc. thesis (University of Alberta, Edmonton).

112. Paris M, et al. (2008) Amphioxus postembryonic development reveals the homology of chordate metamorphosis. *Curr Biol* 18(11):825–830.

113. Braun CB, Northcutt RG (1997) The lateral line system of hagfishes (Craniata: Myxinoidea). *Acta Zool* 78(3):247–268.

114. Wicht H, Northcutt RG (1995) Ontogeny of the head of the Pacific hagfish (*Eptatretus stouti*, Myxinoidea): Development of the lateral line system. *Phil Trans R Soc Lond B Biol Sci* 349:119–134.

115. Miyashita T (2016) Fishing for jaws in early vertebrate evolution: A novel hypothesis of mandibular confinement. *Biol Rev* 91(3):611–657.

116. Shu D, et al. (2003) A new species of *Yunnanozoan* with implications for deuterostome evolution. *Science* 299(5611):1380–1384.

117. Chen J-Y, Huang D-Y, Li C-W (1999) An early Cambrian craniate-like chordate. *Nature* 402(6761):518–522.

118. Mallatt J, Holland N (2013) *Pikaia gracilens* Walcott: Stem chordate, or already specialized in the Cambrian? *J Exp Zool B Mol Dev Evol* 320(4):247–271.

119. Gans C (1993) Evolutionary origin of the vertebrate skull. *The Skull. Volume 2. Patterns of Structural and Systematic Diversity*, eds Hanken J, Hall BK (The University of Chicago Press, Chicago), pp 1–35.

120. Boorman CJ, Shimeld SM (2002) *Pitx* homeobox genes in *Ciona* and amphioxus show left–right asymmetry is a conserved chordate character and define the ascidian adenohypophysis. *Evol Dev* 4(5):354–365.

121. Boorman CJ, Shimeld SM (2002) The evolution of left–right asymmetry in chordates. *BioEssays* 24(11):1004–1011.

122. Patthey C, Schlosser G, Shimeld SM (2014) The evolutionary history of vertebrate cranial placodes – I: Cell type evolution. *Dev Biol* 389:82–97.

123. Wicht H, Lacalli TC (2005) The nervous system of amphioxus: Structure, development, and evolutionary significance. *Can J Zool* 83:122–150.

124. Ruppert EE (2005) Key characters uniting hemichordates and chordates: Homologies or homoplasies? *Can J Zool* 83:8–23.

125. Manni L, Agnoletto A, Zaniolo G, Burighel P (2005) Stomodeal and neurohypophysial placodes in *Ciona intestinalis*: Insights into the origin of the pituitary gland. *J Exp Zool B Mol Dev Evol* 304B(4):324–339.

126. Mazet F, et al. (2005) Molecular evidence from *Ciona intestinalis* for the evolutionary origin of vertebrate sensory placodes. *Dev Biol* 282(2):494–508.

127. Abitua PB, et al. (2015) The pre-vertebrate origins of neurogenic placodes. *Nature* 524(7566):462–465.

128. Schlosser G, Patthey C, Shimeld SM (2014) The evolutionary history of vertebrate cranial placodes II. Evolution of ectodermal patterning. *Dev Biol* 389(1):98–119.

129. Northcutt RG (2004) Taste buds: Development and evolution. *Brain Behav Evol* 64(3):198–206.

130. Kirino M, Parnes J, Hansen A, Kiyohara S, Finger TE (2013) Evolutionary origins of taste buds: Phylogenetic analysis of purinergic neurotransmission in epithelial chemosensors. *Open Biol* 3(3):130015.

131. Braun CB (1998) Schreiner organs: A new craniate chemosensory modality in hagfishes. *J Comp Neurol* 392(2):135–163.

132. Hall BK (2009) *The Neural Crest and Neural Crest Cells in Vertebrate Development and Evolution* (Springer, New York).

133. Couly GF, Coltey PM, Douarin NML (1993) The triple origin of skull in higher vertebrates: A study in quail-chick chimeras. *Development* 117(2):409–429.

134. Sire J-Y, Donoghue PCJ, Vickaryous MK (2009) Origin and evolution of the integumentary skeleton in non-tetrapod vertebrates. *J Anat* 214(4):409–440.

135. Smith MM, Hall BK (1990) Development and evolutionary origins of vertebrate skeletogenic and odontogenic tissues. *Biol Rev* 65(3):277–373.

136. Gillis JA, Alsema EC, Criswell KE (2017) Trunk neural crest origin of dermal denticles in a cartilaginous fish. *Proc Natl Acad Sci* 114(50):13200–13205.

137. Lee RTH, Thiery JP, Carney TJ (2013) Dermal fin rays and scales derive from mesoderm, not neural crest. *Curr Biol* 23(9):R336–R337.

138. Lee RTH, Knapik EW, Thiery JP, Carney TJ (2013) An exclusively mesodermal origin of fin mesenchyme demonstrates that zebrafish trunk neural crest does not generate ectomesenchyme. *Development* 140(14):2923–2932.

139. Mongera A, Nüsslein-Volhard C (2013) Scales of fish arise from mesoderm. *Curr Biol* 23(9):R338–R339.

140. Green SA, Simoes-Costa M, Bronner ME (2015) Evolution of vertebrates as viewed from the crest. *Nature* 520(7548):474–482.

141. Stolfi A, Ryan K, Meinertzhagen IA, Christiaen L (2015) Migratory neuronal progenitors arise from the neural plate borders in tunicates. *Nature* 527(7578):371–374.

142. Abitua PB, Wagner E, Navarrete IA, Levine M (2012) Identification of a rudimentary neural crest in a non-vertebrate chordate. *Nature* 492(7427):104–107.

143. Kourakis MJ, Newman-Smith E, Smith WC (2010) Key steps in the morphogenesis of a cranial placode in an invertebrate chordate, the tunicate *Ciona savignyi*. *Dev Biol* 340(1):134–144.

144. Gans C, Northcutt RG (1983) Neural crest and the origin of vertebrates: A new head. *Science* 220(4594):268–273.

145. Northcutt RG, Gans C (1983) The genesis of neural crest and epidermal placodes: A reinterpretation of vertebrate origins. *Q Rev Biol* 58(1):1–28.

146. Gagnier P-Y (1993) *Sacabambaspis janvieri*, vertébré ordovicien de Bolivie. 1. Analyse morphologique. *Ann Paléontol Vertébrés* 79:19–69.

147. Janvier P (1993) Patterns of diversity in the skull of jawed fishes. *The Skull. Volume II. Patterns of Structural and Systematic Diversity*, eds Hanken J, Hall BK (University of Chicago Press, Chicago).

148. Halstead LB (1973) The heterostracan fishes. *Biol Rev* 48(3):279–332.

149. Janvier P (1974) The structure of the naso-hypophysial complex and the mouth in fossil and extant cyclostomes, with remarks on amphiaspiforms. *Zool Scr* 3(4):193–200.

150. Sugahara F, et al. (2016) Evidence from cyclostomes for complex regionalization of the ancestral vertebrate brain. *Nature* 531(7592):97–100.

151. Nieuwenhuys R, Ten Donkelaar HJ, Nicholson C (1998) *The Central Nervous System of Vertebrates* (Springer, Berlin).

152. Gai Z, Donoghue PCJ, Zhu M, Janvier P, Stampanoni M (2011) Fossil jawless fish from China foreshadows early jawed vertebrate anatomy. *Nature* 476(7360):324–327.

153. Janvier P (1981) *Norselaspis glacialis* n.g., n.sp. et les relations phylogénétiques entre les Kiaeraspidiens (Osteostraci) du Dévonien inféerieur du Spitsberg. *Palaeovertebrata* 11:19–131.

154. Janvier P (1985) *Les Céphalaspides du Spitsberg. Anatomie, phylogénie et systématique des Ostéostracés siluro-dévoniens. Révision des Ostéostracés de la Formation de Wood Bay (Dévonien inférieur du Spitsberg).* (Centre national de la Recherche scientifique, Paris).

155. Goujet D (1984) *Les poissons placodermes du Spitsberg. Arthrodires Dolichothoraci de la Formation de Wood Bay (Devonien Inferieur)* (Centre national de la Recherche scientifique, Paris).

156. Dupret V, Sanchez S, Goujet D, Tafforeau P, Ahlberg PE (2014) A primitive placoderm sheds light on the origin of the jawed vertebrate face. *Nature* 507(7493):500–503.

157. Young GC (1980) A new Early Devonian placoderm from New South Wales, Australia, with a discussion of placoderm phylogeny. *Palaeontogr Abt A*:10–76.

158. Dupret V, Sanchez S, Goujet D, Ahlberg PE (2017) The internal cranial anatomy of *Romundina stellina* Ørvig, 1975 (Vertebrata, Placodermi, Acanthothoraci) and the origin of jawed vertebrates—Anatomical atlas of a primitive gnathostome. *PLoS ONE* 12(2):e0171241.

159. Brazeau MD, Friedman M (2014) The characters of Palaeozoic jawed vertebrates. *Zool J Linn Soc* 170(4):779–821.

160. Kuratani S, Ueki T, Aizawa S, Hirano S (1997) Peripheral development of cranial nerves in a cyclostome, *Lampetra japonica*: Morphological distribution of nerve branches and the vertebrate body plan. *J Comp Neurol* 384(4):483–500.

161. Lindström T (1949) On the cranial nerves of the cyclostomes with special reference to n. trigeminus. *Acta Zool* 30:315–458.

162. Goodrich ES (1930) *Studies on the Structure and Development of Vertebrates* (Dover Publishing, London).

163. Shu D-G, et al. (2003) Head and backbone of the Early Cambrian vertebrate *Haikouichthys*. *Nature* 421(6922):526–529.

164. Kuratani S, Nobusada Y, Horigome N, Shigetani Y (2001) Embryology of the lamprey and evolution of the vertebrate jaw: Insights from molecular and developmental perspectives. *Phil Trans R Soc Lond Ser B* 356(1414):1615–1632.

165. Shigetani Y, et al. (2002) Heterotopic shift of epithelial-mesenchymal interactions in vertebrate jaw evolution. *Science* 296(5571):1316–1319.

166. Kuratani S (2012) Evolution of the vertebrate jaw from developmental perspectives. *Evol Dev* 14(1):76–92.

167. Fernholm B, Quattrini AM (2008) A new species of hagfish (Myxinidae: *Eptatretus*) associated with deep-sea coral habitat in the western North Atlantic. *Copeia* 2008(1):126–132.

168. Fernholm B (1991) *Eptatretus eos*: A new species of hagfish (Myxinidae) from the Tasman Sea. *Jpn J Ichthyol* 38:115–118.

169. Kuo C-H, Lee SC, Mok H-K (2010) A new species of hagfish *Eptatretus rubicundus* (Myxinidae: Myxiniformes) from Taiwan, with reference to its phylogenetic position based on its mitochondrial DNA sequence. *Zool Stud* 49(6):855–864.

170. Mok H-K (2001) Nasal-sinus papillae of hagfishes and their taxonomic implications. *Zool Stud* 40:355–364.

171. Lamb TD, Collin SP, Pugh EN (2007) Evolution of the vertebrate eye: opsins, photoreceptors, retina and eye cup. *Nat Rev Neurosci* 8(12):960–976.

172. Gabbott SE, Aldridge RJ, Theron JN (1995) A giant conodont with preserved muscle tissue from the Upper Ordovician of South Africa. *Nature* 374(6525):800–803.

173. Wright GM, Youson JH (1982) Ultrastructure of mucocartilage in the larval anadromous sea lamprey, *Petromyzon marinus* L. *Am J Anat* 165(1):39–51.

174. Wright GM, Youson JH (1983) Ultrastructure of cartilage from young adult sea lamprey, *Petromyzon marinus* L: A new type of vertebrate cartilage. *Am J Anat* 167(1):59–70.

175. Armstrong LA, Wright GM, Youson JH (1987) Transformation of mucocartilage to a definitive cartilage during metamorphosis in the sea lamprey, *Petromyzon marinus*. *J Morphol* 194(1):1–21.

176. Robson P, Wright GM, Youson JH, Keeley FW (1997) A family of non–collagen-based cartilages in the skeleton of the sea lamprey, *Petromyzon marinus*. *Comp Biochem Physiol B Biochem Mol Biol* 118(1):71–78.

177. Martin WM, Bumm LA, McCauley DW (2009) Development of the viscerocranial skeleton during embryogenesis of the sea lamprey, *Petromyzon marinus*. *Dev Dyn* 238(12):3126–3138.

178. Young GC (2008) Number and arrangement of extraocular muscles in primitive gnathostomes: evidence from extinct placoderm fishes. *Biol Lett* 4(1):110–114.

179. Janvier P (1975) Les yeux des Cyclostomes fossiles et le problème de l'origine des Myxinoïdes. *Acta Zool* 56(1):1–9.

180. Janvier P, Arsenault M (2002) Palaeobiology: Calcification of early vertebrate cartilage. *Nature* 417(6889):609–609.

181. van der Brugghen G (2010) New observations on the Silurian anaspid *Lasanius problematicus* Traquair. *Foss Quarry Artic* 1:1–10.

182. Shu D-G, et al. (1999) Lower Cambrian vertebrates from south China. *Nature* 402(6757):42–46.

183. Conway Morris S, Caron J-B (2012) *Pikaia gracilens* Walcott, a stem-group chordate from the Middle Cambrian of British Columbia. *Biol Rev* 87(2):480–512.

184. Jørgensen JM, Shichiri M, Geneser FA (1998) Morphology of the hagfish inner ear. *Acta Zool* 79(3):251–256.

185. Janvier P, Lund R (1983) *Hardistiella montanensis* n. gen. et sp. (Petromyzontida) from the Lower Carboniferous of Montana, with remarks on the affinities of the lampreys. *J Vert Paleontol* 2(4):407–413.

186. Halstead LB (1971) The presence of a spiracle in the Heterostraci (Agnatha). *Zool J Linn Soc* 50(2):195–197.

187. Young GC (1991) The first armoured agnathan vertebrates from the Devonian of Australia. *Early Vertebrates and Related Problems of Evolutionary Biology*, eds Chang MM, Liu YH, Zhang GR (Science Press, Beijing), pp 67–85.

188. Lundgren M, Blom H (2013) Phylogenetic relationships of the cyathaspidids (Heterostraci). *GFF* 135(1):74–84.

189. Randle E, Sansom RS (2017) Phylogenetic relationships of the 'higher heterostracans' (Heterostraci: Pteraspidiformes and Cyathaspididae), extinct jawless vertebrates. *Zool J Linn Soc*. 181(4):910–926.

190. Randle E, Sansom RS (2017) Exploring phylogenetic relationships of Pteraspidiformes heterostracans (stem-gnathostomes) using continuous and discrete characters. *J Syst Palaeontol* 15(7):583–599.

191. Novitskaya L (1971) *Les Amphiaspides (Heterostraci) du Débonien de la Sibérie* (Éditions du Centre national de la Recherche Scientifique, Paris).

192. Novitskaya LI (2008) Evolution of taxonomic diversity in amphiaspids (Agnatha, Heterostraci: Amphiaspidiformes) and the causes of extinction in ecologically favorable conditions. *Paleontol J* 42(2):181–191.

193. Jefferies RPS (1987) *The Ancestry of the Vertebrates* (Cambridge University Press, Cambridge ; New York, NY, USA).

194. Kuo C-H, Huang S, Lee S-C (2003) Phylogeny of hagfish based on the mitochondrial 16S rRNA gene. *Mol Phylogenet Evol* 28(3):448–457.

195. Aldridge RJ, Briggs DEG, Smith MP, Clarkson ENK, Clark NDL (1993) The anatomy of conodonts. *Phil Trans R Soc B Biol Sci* 340(1294):405–421.

196. Janvier P (2004) Early specializations in the branchial apparatus of jawless vertebrates: A consideration of gill number and size. *Recent Advances in the Origin and Early Radiation of Vertebrates*, eds Arratia G, Wilson MVH, Cloutier R (Verlag Dr. Friedrich Pfeil, Munich), pp 29–52.

197. Fernholm B (1998) Hagfish systematics. *The Biology of Hagfishes*, eds Jørgensen JM, Lomholt JP, Weber RE, Malte H (Chapman, London), pp 33–44.

198. Blom H, Märss T, Miller CG (2001) Silurian and earliest Devonian birkeniid anaspids from the Northern Hemisphere. *Earth Environ Sci Trans R Soc Edinb* 92(3):263–323.

199. Newman MJ (2002) A new naked jawless vertebrate from the Middle Devonian of Scotland. *Palaeontology* 45(5):933–941.

200. Newman MJ, Trewin NH (2001) A new jawless vertebrate from the Middle Devonian of Scotland. *Palaeontology* 44(1):43–51.

201. Lund R, Janvier P (1986) A second lamprey from the Lower Carboniferous (Namurian) of Bear Gulch, Montana (U.S.A.). *Geobios* 19(5):647–652.

202. Kuratani S, Adachi N, Wada N, Oisi Y, Sugahara F (2013) Developmental and evolutionary significance of the mandibular arch and prechordal/premandibular cranium in vertebrates: Revising the heterotopy scenario of gnathostome jaw evolution. *J Anat* 222(1):41–55.

203. Janvier P (1985) Les Thyestidiens (Ostéostraci) du Silurien de Saaremaa (Estonie). Première partie: morphologie et anatomie. *Ann Paléontol* 71:83–147.

204. Davison W (2015) The hagfish heart. *Hagfish Biology*, Marine Biology. (CRC Press), pp 149–160.

205. Glover CN, Bucking C, Wood CM (2011) Adaptations to in situ feeding: Novel nutrient acquisition pathways in an ancient vertebrate. *Proc R Soc Lond B Biol Sci* 278(1721):3096–3101.

206. Forster ME (1997) The blood sinus system of hagfish: Its significance in a low-pressure circulation. *Comp Biochem Physiol A Physiol* 116(3):239–244.

207. Glover CN, Bucking C, Wood CM (2013) The skin of fish as a transport epithelium: A review. *J Comp Physiol B* 183(7):877–891.

208. Cori CI (1906) Das Blutgefäßsystem des jungen Ammocoetes. *Arb Aus Den Zool Instituten Univ Wien* 16:217–312.

209. Cole FJ (1926) A monograph on the general morphology of the myxinoid fishes, based on a study of *Myxine*. Part VI. The morphology of the vascular system. *Trans R Soc Edinb* 54:683–757.

210. Janvier P, Percy LR, Potter IC (1991) The arrangement of the heart chambers and associated blood vessels in the Devonian osteostracan *Norselaspis glacialis*. A reinterpretation based on recent studies of the circulatory system in lampreys. *J Zool* 223(4):567–576.

211. Ayers H, Jackson CM (1901) Morphology of the Myxinoidei. I. Skeleton and musculature. *J Morphol* 17:185–225.

212. Cole FJ (1905) A monograph on the general morphology of the myxinoid fishes, based on a study of *Myxine*. Part I. The anatomy of the skeleton. *Trans R Soc Edinb* 41:749–791.

213. Stensiö EA (1939) A new anaspid from the Upper Devonian of Scaumenac Bay in Canada with remakrs on the other anaspids. *K Sven Vent Handl* 18:1–25.

214. Sansom RS, Gabbott SE, Purnell MA (2013) Unusual anal fin in a Devonian jawless vertebrate reveals complex origins of paired appendages. *Biol Lett* 9(3):20130002.

215. Janvier P, Desbiens S, Willett JA, Arsenault M (2006) Lamprey-like gills in a gnathostome-related Devonian jawless vertebrate. *Nature* 440(7088):1183–1185.

216. Vladykov VD (1973) A female sea lamprey (*Petromyzon marinus*) with a true anal fin, and the question of the presence of an anal fin in Petromyzonidae. *Can J Zool* 51(2):221–224.

217. Märss T, Turner S, Karatajūtė-Talimaa V (2007) *Handbook of Paleoichthyology / "Agnatha" II. Thelodonti.* (Verlag Dr. Friedrich Pfeil, München).

218. Wilson MVH, Hanke GF, Märss T (2007) Paired fins of jawless vertebrates and their homologies across the "agnathan"-gnathostome transition. *Major Transitions in Vertebrate Evolution*, eds Anderson JS, Sues H-D (Indiana University Press, Bloomington), pp 122–149.

219. Kuratani S (2008) Evolutionary developmental studies of cyclostomes and the origin of the vertebrate neck. *Dev Growth Differ* 50:S189–S194.

220. Janvier P, Arsenault M, Desbiens S (2004) Calcified cartilage in the paired fins of the osteostracan *Escuminaspis laticeps* (Traquair 1880), from the Late Devonian of Miguasha (Québec, Canada), with a consideration of the early evolution of the pectoral fin endoskeleton in vertebrates. *J Vert Paleontol* 24(4):773–779.

221. Johanson Z (2002) Vascularization of the osteostracan and antiarch (Placodermi) pectoral fin: Similarities, and implications for placoderm relationships. *Lethaia* 35(2):169–186.

222. Bertin M, et al. (2007) Origin of the genes for the isoforms of creatine kinase. *Gene* 392(1–2):273–282.

223. Suzuki T, et al. (2004) Evolution and divergence of the genes for cytoplasmic, mitochondrial, and flagellar creatine kinases. *J Mol Evol* 59(2):218–226.

224. Ellington WR, Suzuki T (2007) Early evolution of the creatine kinase gene family and the capacity for creatine biosynthesis and membrane transport. *Creatine and Creatine Kinase in Health and Disease*, Subcellular Biochemistry., eds Salomons GS, Wyss M (Springer Netherlands), pp 17–26.

225. DeLigio JT, Ellington WR (2006) The capacity for the de novo biosynthesis of creatine is present in the tunicate *Ciona intestinalis* and is likely widespread in other protochordate and invertebrate groups. *Comp Biochem Physiol Part D Genom Proteom* 1(2):167–178.

226. Cameron CB, Bishop CD (2012) Biomineral ultrastructure, elemental constitution and genomic analysis of biomineralization-related proteins in hemichordates. *Proc R Soc Lond B Biol Sci* 279(1740):3041–3048.

227. Avallone B, et al. (2007) Morphogenesis of otoliths during larval development in brook lamprey, *Lampetra planeri*. *Ital J Zool* 74(3):247–258.

228. Burrow CJ, Valiukevičius J (2005) Diversity of tissues in acanthodians with *Nostolepis*-type histological structure. *Acta Palaeontol Pol* 50(3):635–649.

229. Valiukevičius J (1995) Acanthodian histology: Some significant aspects in taxonomical and phylogenetical research. *Geobios* 28:157–159.

230. Gillis JA, Donoghue PCJ (2007) The homology and phylogeny of chondrichthyan tooth enameloid. *J Morphol* 268(1):33–49.

231. Hall BK (2015) *Bones and Cartilage: Developmental and Evolutionary Skeletal Biology. 2nd edition.* (Academic Press, London).

232. Witten PE, Huysseune A, Hall BK (2010) A practical approach for the identification of the many cartilaginous tissues in teleost fish. *J Appl Ichthyol* 26(2):257–262.

233. Sire J-Y, Huysseune A (2003) Formation of dermal skeletal and dental tissues in fish: a comparative and evolutionary approach. *Biol Rev* 78(2):219–249.

234. Donoghue PCJ, Sansom IJ (2002) Origin and early evolution of vertebrate skeletonization. *Microsc Res Tech* 59(5):352–372.

235. Donoghue PCJ, Sansom IJ, Downs JP (2006) Early evolution of vertebrate skeletal tissues and cellular interactions, and the canalization of skeletal development. *J Exp Zool B Mol Dev Evol* 306B(3):278–294.

236. Wright GM, Armstrong LA, Jacques AM, Youson JH (1988) Trabecular, nasal, branchial, and pericardial cartilages in the sea lamprey, *Petromyzon marinus*: Fine structure and immunohistochemical detection of elastin. *Am J Anat* 182(1):1–15.

237. Wang N-Z, Donoghue PCJ, Smith MM, Sansom IJ (2005) Histology of the galeaspid dermoskeleton and endoskeleton, and the origin and early evolution of the vertebrate cranial endoskeleton. *J Vert Paleontol* 25(4):745–756.

238. Zhu M, Janvier P (1998) The histological structure of the endoskeleton in galeaspids (Galeaspida, Vertebrata). *J Vert Paleontol* 18(3):650–654.

239. Giles S, Rücklin M, Donoghue PCJ (2013) Histology of “placoderm” dermal skeletons: Implications for the nature of the ancestral gnathostome. *J Morphol* 274(6):627–644.

240. Downs JP, Donoghue PCJ (2009) Skeletal histology of *Bothriolepis canadensis* (Placodermi, Antiarchi) and evolution of the skeleton at the origin of jawed vertebrates. *J Morphol* 270(11):1364–1380.

241. Rücklin M, et al. (2012) Development of teeth and jaws in the earliest jawed vertebrates. *Nature* 491(7426):748–751.

242. Sansom IJ, Smith MP, Smith MM (1994) Dentine in conodonts. *Nature* 368(6472):591–591.

243. Murdock DJE, et al. (2013) The origin of conodonts and of vertebrate mineralized skeletons. *Nature* 502(7472):546–549.

244. Sansom IJ, Donoghue PCJ, Albanesi G (2005) Histology and affinity of the earliest armoured vertebrate. *Biol Lett* 1(4):446–449.

245. Donoghue PCJ (1998) Growth and patterning in the conodont skeleton. *Phil Trans R Soc Lond B Biol Sci* 353(1368):633–666.

246. Ørvig T (1989) Histologic studies of ostracoderms, placoderms and fossil elasmobranchs. 6. Hard tissues of Ordovician vertebrates. *Zool Scr* 18(3):427–446.

247. Sansom IJ, Smith MP, Smith MM, Turner P (1997) *Astraspis*: The anatomy and histology of an Ordovician fish. *Palaeontology* 40(3):625–643.

248. Keating JN, Marquart CL, Donoghue PCJ (2015) Histology of the heterostracan dermal skeleton: Insight into the origin of the vertebrate mineralised skeleton. *J Morphol* 276(6):657–680.

249. Qu Q, Blom H, Sanchez S, Ahlberg P (2015) Three-dimensional virtual histology of Silurian osteostracan scales revealed by synchrotron radiation microtomography. *J Morphol* 276(8):873–888.

250. Sansom RS (2008) The origin and early evolution of the Osteostraci (Vertebrata): A phylogeny for the Thyestiida. *J Syst Palaeontol* 6(3):317–332.

251. Sansom RS (2009) Phylogeny, classification and character polarity of the Osteostraci (Vertebrata). *J Syst Palaeontol* 7(1):95–115.

252. Qu Q, Haitina T, Zhu M, Ahlberg PE (2015) New genomic and fossil data illuminate the origin of enamel. *Nature* 526(7571):108–111.

253. Hall BK, Miyake T (2000) All for one and one for all: Condensations and the initiation of skeletal development. *BioEssays* 22(2):138–147.

254. Witten PE, Huysseune A (2009) A comparative view on mechanisms and functions of skeletal remodelling in teleost fish, with special emphasis on osteoclasts and their function. *Biol Rev* 84(2):315–346.

255. Ørvig T (1980) Histologic studies of ostracoderms, placoderms and fossil elasmobranchs. *Zool Scr* 9(1–4):219–239.

256. Purnell MA, von Bitter PH (1992) Blade-shaped conodont elements functioned as cutting teeth. *Nature* 359(6396):629–631.

257. Young GC (1984) Reconstruction of the jaws and braincase in the Devonian placoderm fish *Bothriolepis*. *Palaeontology* 27(3):635–661.

258. Coates MI, Sequeira SEK (1998) The braincase of a primitive shark. *Earth Environ Sci Trans R Soc Edinb* 89(2):63–85.

259. Coates MI, Gess RW, Finarelli JA, Criswell KE, Tietjen K (2017) A symmoriiform chondrichthyan braincase and the origin of chimaeroid fishes. *Nature* 541(7636):208–211.

260. Zhu M, Yu X, Ahlberg PE (2001) A primitive sarcopterygian fish with an eyestalk. *Nature* 410(6824):81–84.

261. Basden AM, Young GC, Coates MI, Ritchie A (2000) The most primitive osteichthyan braincase? *Nature* 403(6766):185–188.

262. Oisi Y, Ota KG, Fujimoto S, Kuratani S (2013) Development of the chondrocranium in hagfishes, with special reference to the early evolution of vertebrates. *Zool Sci* 30(11):944–961.

263. Bardack D (1998) Relationships of living and fossil hagfishes. *The Biology of Hagfishes*, eds Jørgensen JM, Lomholt JP, Weber RE, Malte H (Chapman, London), pp 3–14.

264. Germain D, Sanchez S, Janvier P, Tafforeau P (2014) The presumed hagfish *Myxineidus gononorum* from the Upper Carboniferous of Montceau-les-Mines (Saône-et-Loire, France): New data obtained by means of Propagation Phase Contrast X-ray Synchrotron Microtomography. *Ann Paléontol* 100(2):131–135.

265. Dawson JA (1963) The oral cavity, the 'jaws' and the horny teeth of *Myxine glutinosa*. *The Biology of Myxine*, eds Brodal A, Fänge R (Universtetsforlaget, Oslo), pp 231–255.

266. Marinelli W, Strenger A (1956) *Vergleichende Anatomie und Morphologie der Wirbeltiere. II Lieferung. Myxine glutinosa (L).*

267. Marinelli W, Strenger A (1954) *Vergleichende Anatomie und Morphologie der Wirbeltiere. I Lieferung. Petromyzon marinus (L).* (1-80).

268. Criswell KE, Coates MI, Gillis JA (2017) Embryonic origin of the gnathostome vertebral skeleton. *Proc R Soc B* 284(1867):20172121.

269. Ota KG, Fujimoto S, Oisi Y, Kuratani S (2011) Identification of vertebra-like elements and their possible differentiation from sclerotomes in the hagfish. *Nat Commun* 2:373.

270. Janvier P, Blieck A (1979) New data on the internal anatomy of the Heterostraci (Agnatha), with general remarks on the phylogeny of the Craniata. *Zool Scr* 8(1–4):287–296.

271. Janvier P, Sansom RS (2016) Fossil hagfishes, fossil cyclostomes, and the lost world of "ostracoderms." *Hagfish Biology*, eds Edwards SL, Goss GG (CRC Press, Boca Raton), pp 73–93.

272. Wilson MVH, Caldwell MW (1993) New Silurian and Devonian fork-tailed "thelodonts" are jawless vertebrates with stomachs and deep bodies. *Nature* 361(6411):442–444.

273. Wilson MVH, Caldwell MW (1998) The Furcacaudiformes: A new order of jawless vertebrates with thelodont scales, based on articulated Silurian and Devonian fossils from northern Canada. *J Vert Paleontol* 18(1):10–29.

274. Donoghue PCJ, Smith MP (2001) The anatomy of *Turinia pagei* (Powrie), and the phylogenetic status of the Thelodonti. *Earth Environ Sci Trans R Soc Edinb* 92(1):15–37.

275. Chen J-Y, Dzik J, Edgecombe GD, Ramsköld L, Zhou G-Q (1995) A possible Early Cambrian chordate. *Nature* 377(6551):720–722.

276. Ferry JD (1941) A fibrous protein from the slime of the hagfish. *J Biol Chem* 138(1):263–268.

277. Fudge DS, Gosline JM (2004) Molecular design of the α–keratin composite: Insights from a matrix–free model, hagfish slime threads. *Proc R Soc Lond B Biol Sci* 271(1536):291–299.

278. Fudge DS, Gardner KH, Forsyth VT, Riekel C, Gosline JM (2003) The mechanical properties of hydrated intermediate filaments: Insights from hagfish slime threads. *Biophys J* 85(3):2015–2027.

279. Fudge DS, Levy N, Chiu S, Gosline JM (2005) Composition, morphology and mechanics of hagfish slime. *J Exp Biol* 208(24):4613–4625.

280. Fudge DS, Hillis S, Levy N, Gosline JM (2010) Hagfish slime threads as a biomimetic model for high performance protein fibres. *Bioinspir Biomim* 5(3):035002.

281. Koch EA, Spitzer RH, Pithawalla RB (1991) Structural forms and possible roles of aligned cytoskeletal biopolymers in hagfish (slime eel) mucus. *J Struct Biol* 106(3):205–210.

282. Winegard T, et al. (2014) Coiling and maturation of a high-performance fibre in hagfish slime gland thread cells. *Nat Commun* 5:3534.

283. Saitta ET, et al. (2017) Low fossilization potential of keratin protein revealed by experimental taphonomy. *Palaeontology* 60(4):547–556.
